# Supplementary material for: Genome-wide association meta-analysis identifies 29 new acne susceptibility loci
Source: Nat Commun. 2022 Feb 7;13:702. doi: 10.1038/s41467-022-28252-5 (PMC8821634; doi:10.1038/s41467-022-28252-5)
Supplement: Supplementary file 1 — Supplementary Information [file 41467_2022_28252_MOESM1_ESM.pdf]

Supplementary Material for
Genome-wide association meta-analysis identifies 29 new acne susceptibility loci

Brittany L. Mitchell[1,2*], Jake R. Saklatvala[3*], Nick Dand[3,4], Fiona A. Hagenbeek[5,6], Xin Li[7,8], Josine L. Min[9,10], Laurent Thomas[11,12,13], Meike Bartels[5,6], Jouke Jan Hottenga[5,6], Michelle K. Lupton[1], Dorret I. Boomsma[5,6], Xianjun Dong[14,15], Kristian Hveem[16,17,18], Mari Løset[16,19], Nicholas G. Martin[1], Jonathan Barker[20], Jiali Han[7,8], Catherine Smith[20], Miguel E. Rentería[1,2†] & Michael A. Simpson[3†]

1 Department of Genetics and Computational Biology, QIMR Berghofer Medical Research Institute, Brisbane, Australia
2 School of Biomedical Sciences, Faculty of Health, Queensland University of Technology (QUT), Brisbane, Australia
3 Department of Medical and Molecular Genetics, King's College London, London, United Kingdom
4 Health Data Research UK, London, United Kingdom
5 Department of Biological Psychology, Vrije Universiteit Amsterdam, Amsterdam, the Netherlands
6 Amsterdam Public Health research institute, Amsterdam, the Netherlands
7 Department of Epidemiology, Indiana University Richard M. Fairbanks School of Public Health, Indianapolis, US
8 Indiana University Melvin and Bren Simon Comprehensive Cancer Center, Indianapolis, US
9 MRC Integrative Epidemiology Unit, University of Bristol, Bristol, UK
10 Population Health Sciences, Bristol Medical School, University of Bristol, Bristol, UK
11 Department of Clinical and Molecular Medicine, Norwegian University of Science and Technology, Trondheim, Norway
12 K. G. Jebsen Center for Genetic Epidemiology, Department of Public Health and Nursing, Faculty of Medicine and Health, Norwegian University of Science and Technology, Trondheim, Norway
13 BioCore - Bioinformatics Core Facility, Norwegian University of Science and Technology, Trondheim. Norway
14 Genomics and Bioinformatics Hub, Brigham and Women's Hospital, Boston, MA, USA
15 Department of Neurology, Brigham and Women's Hospital, Boston, MA, USA
16 K.G. Jebsen Center for Genetic Epidemiology, Department of Public Health and Nursing, NTNU, Norwegian University of Science and Technology, Trondheim, Norway
17 HUNT Research Centre, Department of Public Health and Nursing, Norwegian University of Science and Technology, Levanger, Norway
18 Levanger Hospital, Nord-Trøndelag Hospital Trust, Levanger, Norway
19 Department of Dermatology, Clinic of Orthopaedy, Rheumatology and Dermatology, St. Olavs Hospital, Trondheim University Hospital, Trondheim, Norway
20 St John's Institute of Dermatology, Faculty of Life Sciences & Medicine, King's College London, London, United Kingdom

* Authors contributed equally
† Authors contributed equally
Correspondence: Michael A. Simpson (michael.simpson@kcl.ac.uk) and Miguel E. Rentería (miguel.renteria@qimrberghofer.edu.au)

1. SUPPLEMENTARY NOTE: ACNE COHORT DETAILS

1. ALSPAC

ALSPAC (The Avon Longitudinal Study of Parents and Children) is a transgenerational prospective observational study investigating influences on health and development across the life course. Pregnant women resident in Avon, UK with expected dates of delivery 1st April 1991 to 31st December were invited to take part in the study. Of the 14,541 enrolled pregnancies, there were a total of 14,676 foetuses, resulting in 14,062 live births and 13,988 children who were alive at 1 year of age [1,2]. When the oldest children were approximately 7 years of age, an additional 913 pregnancies were enrolled resulting in a total sample size of 15,589 foetuses. Of these 14,901 were alive at 1 year of age. Participants attended clinics at the University of Bristol and filled out follow-up questionnaires at various intervals. Please note that the study website contains details of all the data that is available through a fully searchable data dictionary and variable search tool" and reference the following webpage: http://www.bristol.ac.uk/alspac/researchers/our-data/

Ethical approval for the study was obtained from the ALSPAC Ethics and Law Committee and the Local Research Ethics Committees. Consent for biological samples has been collected in accordance with the Human Tissue Act (2004). Informed consent for the use of data collected via questionnaires and clinics was obtained from participants following the recommendations of the ALSPAC Ethics and Law Committee at the time.

Children were genotyped using the Illumina HumanHap550 quad genome-wide SNP genotyping platform (Illumina Inc., San Diego, CA, USA) by the Wellcome Trust Sanger Institute (WTSI; Cambridge, UK) and the Laboratory Corporation of America (LCA, Burlington, NC, USA). Individuals were excluded on the basis of incorrect gender assignment, abnormal heterozygosity (<0.320 or >0.345 for WTSI data; <0.310 or >0.330 for LCA data), high missingness (>3 %), cryptic relatedness (>10 % identity by descent) and non-European ancestry (detected by MDS analysis). Following QC, the final directly genotyped dataset contained 500,527 SNP loci. Mothers were genotyped using the Illumina Human660W-quad genome-wide SNP genotyping

platform (Illumina Inc., San Diego, CA, USA) at the Centre National de Génotypage (CNG; Paris, France). Individuals were excluded based on non-European ancestry, missingness, relatedness, gender mismatches and heterozygosity. PLINK (v1.07) was used to carry out QC measures on an initial set of 10,015 subjects and 557,124 directly genotyped SNPs. Following QC, the final directly genotyped dataset contained 526,688 SNP loci. The ALSPAC genotype data generated using the Illumina HumanHap550 quad (children) and Illumina human660W quad (mothers) has been jointly phased using SHAPEIT v2 (which uses relationship information to improve phasing accuracy) and imputed to the HRC panel (HRC.r1.1) [3] using the Michigan imputation server [4].

Acne data was collected via clinical assessment at various time-points. Here, the 13.5 years old time-point was selected as a compromise between the number of individuals with data at the time-point (at each time-point, there was attrition of patients) and having an older age to allow for the maximum number of acne cases that manifest during puberty. If any acne variants were present on any of the three main sites (face, chest, back) the severity was recorded according to the "Acne Grading Guide" [5] as trivial, mild, moderate or severe. There were 5,069 participants with GWA data and acne data at age 13.5 years. Acne cases were defined as individuals with "severe" and "moderate" acne (193) and controls as "trivial" and "no acne" (3,772). GWAS was run in SNPTESTv2.5.2 using age, sex and 20 ancestry principal components as covariates.

2. FINNGEN

FinnGen data consists of 135,638 Finnish individuals from FinnGen Data Freeze 3, which includes prospective epidemiological and disease-based cohorts as well as hospital biobank samples (listed at https://finngen.gitbook.io/documentation/). The data were linked by the unique national personal identification numbers to national hospital discharge, death, and medication reimbursement registries. The Ethical Review Board of the Hospital District of Helsinki and Uusimaa approved the FinnGen study protocol (HUS/990/2017). Participation in FinnGen utilizing biobank samples is always voluntary and sample donors are free to cancel, change or restrict their consent to biobank research at any time.

Chip genotype data processing and QC Samples were genotyped with Illumina (Illumina Inc., San Diego, CA, USA) and Affymetrix arrays (Thermo Fisher Scientific, Santa Clara, CA, USA). Genotype calls were made with GenCall and zCall algorithms for Illumina and AxiomGT1 algorithm for Affymetrix data. Chip genotyping data produced with previous chip platforms and reference genome builds were lifted over to build version 38 (GRCh38/hg38) following the protocol described here: dx.doi.org/10.17504/protocols.io.nqtddwn. In sample-wise quality control, individuals with ambiguous gender, high genotype missingness (>5%), excess heterozygosity (+-4SD) and non-Finnish ancestry were excluded. In variant-wise quality control variants with high missingness (>2%), low HWE P-value (<1e-6) and minor allele count, MAC<3 were excluded. Prior to imputation, chip genotyped samples were pre-phased with Eagle 2.3.5 (https://data.broadinstitute.org/alkesgroup/Eagle/) with the default parameters, except the number of conditioning haplotypes was set to 20,000. Genotype imputation was done with the population-specific SISu v3 reference panel. A variant call set was produced with the GATK HaplotypeCaller algorithm by following GATK best practices for variant calling. Genotype-, sample- and variant-wise QC was applied in an iterative manner by using the Hail framework v0.1, and the resulting high-quality WGS data for 3,775 individuals were phased with Eagle 2.3.5 as described in the previous section. Genotype imputation was carried out by using the population-specific SISu v3 imputation reference panel with Beagle 4.1 (version 08Jun17.d8b). Post-imputation quality-control involved checking expected conformity of the imputation INFO-value distribution, MAF differences between the target dataset and the imputation reference panel and checking chromosomal continuity of the imputed genotype calls.

Data from various nationwide registries (listed at https://finngen.gitbook.io/documentation/methods/endpoints) were used to define disease endpoints, harmonising over ICD revisions 8, 9 and 10. These registries spanning decades were electronically linked to the cohort baseline data using the unique national personal identification numbers assigned to all Finnish citizens and residents. We selected the acne endpoint (ICD-10: L70), which had 665 cases. This endpoint excluded any individual with ICD-10 codes L60-75 (Disorders of skin appendages) from controls, leaving 131,216 individuals.

SAIGE (r3 release) software was used for running the R3 GWAS. The model included sex, age, 10 PCs and genotyping batch as covariates. Downloaded FinnGen summary statistics were lifted over from build hg38 to hg19 using the UCSC liftover tool (downloaded 11 June 2020; http://genome.ucsc.edu). 16,868,483 variants were successfully lifted over, and 79,728 were unable to be mapped.

3. HARVARD

Acne data were collected through questionnaire across three cohorts; the Nurses' Health Study (NHS; female only[6]) starting in 1976, NHS2 (female only[6]) starting in 1989, and the Health Professionals Follow-up Study (HPFS; male-only) in 1986 [7]. All participants were asked "Have you ever had any of the following clinician-diagnoses illnesses?" with severe teenage acne as a check box option. Informed consent was obtained after ethical approval of the protocol of the study was approved by the Institutional Review Board of Brigham and Women's Hospital and the Harvard T.H. Chan School of Public Health. Participants were genotyped across four chips: OncoArray (442 cases, 4,763 controls), OmniExpress (189 cases, 2,943 controls), IlluminaHumanHap (107 cases, 1,144 controls) and HuCore2 (745 cases, 6,332 controls) and imputed using Michigan Imputation Server and the 1000 Genomes mixed population Project Phase 3 Integrated Release Version 5 (2010–11 data freeze, 2012-03-14 haplotypes) as reference panels. GWAS were run using the Rvtests software [8] on individuals of European ancestry while controlling for age, cohort (sex) and 10 PCs. All 4 datasets were filtered for MAF<0.01 and imputation accuracy>0.7 before meta-analysis.

4. HUNT

The Trøndelag Health Study (HUNT) is a population-based cohort study carried out at four time points over approximately 40 years (HUNT1 [1984-1986], HUNT2 [1995-1997] and HUNT3 [2006-2008] and HUNT4 [2017-2019])[9]. All inhabitants aged 20 years and over residing in Trøndelag County in Norway were invited to participate. The surveys included clinical measurements, blood sampling and questionnaires on general health measures and a broad range of self-reported diseases and symptoms. Participants were also linked to regional- and national health registries through a

unique national identification number, which enabled acne cases to be identified by searching for acne ICD codes. Participation in the HUNT Study is based on informed consent, and the study has been approved by the Data Inspectorate and the Regional Ethics Committee for Medical Research in Norway (REK 2014/144 and 2015/586).

Participants from HUNT2 and HUNT3 were genotyped using one of three different Illumina HumanCoreExome arrays (HumanCoreExome12 v1.0, HumanCoreExome12 v1.1 and UM HUNT Biobank v1.0). Genotype calling was performed with GenTrain v.2.0 in GenomeStudio v.2011.1 (Illumina). Samples with <99% genotype calls, with large chromosomal copy number variants, contamination >2.5% as estimated with BAF Regress[10], with genotypic and phenotypic sex discordance, and not of European ancestry were excluded, leaving 69,422 genotyped subjects. Genetic variants out of Hardy-Weinberg equilibrium (p-value <0.0001) were excluded.

Imputation was performed on samples of recent European ancestry using Minimac4 (v1.0.2, https://genome.sph.umich.edu/wiki/Minimac4) from a merged reference panel constructed from the Haplotype Reference Consortium (HRC) panel (v1.1) and a local reference panel based on 2,202 whole-genome sequenced HUNT participants, resulting in 24.9 million SNPs (R^2>=0.3). Imputation on the 1000 genomes project was done using Minimac4 (v1.0.2), resulting in 15.1 million SNPs (R^2>=0.3).

The following ICD-10 codes were used to define acne cases: L70.0 (acne vulgaris), L70.1 (acne conglobata), L70.8 (other acne), L70.9 (acne, unspecified). The ICD-9 code 706.1 was also used as it was equivalent to L70.0, L70.1 and L70.8. There were 456 individuals in HUNT with at least one of the specified codes, with the remaining 68,590 individuals defined as controls. GWAS was run in SAIGE v0.29 [11], using sex, birth year, genotyping batch and 4 ancestry principal components as covariates. Variants with MAF >1% were included in the analyses, and dosages were used for imputed variants.

5. King’s College London (KCL)

The KCL dataset is a case-control study consisting of 5,602 severe acne cases recruited through a network of hospital-based dermatologists within the United Kingdom and 21,120 unselected population controls. The study was designed in accordance with the Declarations of Helsinki, and ethical approval was obtained from

the NRES Committee London-Westminster (reference CLRN 05/Q0702/114). It is a meta-analysis of two rounds of recruitment, the first round consisting of 1,779 acne cases and 4,976 controls from the UK population[12] and the second round consisting of 3,823 cases and 16,144 unselected population controls. Each participant provided informed consent.

The diagnostic criteria remained the same for both rounds of recruitment, with one or more of the following criteria required for diagnosis: (a) nodulocystic disease; (b) ≥5 points in any body region assessed by the validated Leeds clinical acne score that uses a colour photographic acne grading scheme to evaluate the severity of involvement of body regions (face 0–12, chest 0–8 and back 0–8); (c) requiring treatment with isotretinoin; and (d) presence of rare and severe forms of acne.

Genotyping, QC and imputation is described in Navarini et al. and Petridis et al. for the respective studies. For both studies association testing was performed with a logistic Wald association test (EPACTS), including the first four ancestry principal components, sex and QC/imputation batch as covariates. Following this, a standard error-weighted meta-analysis was performed across the two datasets.

6. MASS GENERAL BRIGHAM BIOBANK (PARTNERS BIOBANK)

Samples, genomic data, and health information were obtained from the Mass General Brigham Biobank, a samples biorepository of consented patients at Mass General Brigham (parent organization of Massachusetts General Hospital and Brigham and Women's Hospital). Data for over 35,000 volunteers is available (with informed consent)[13]. The Partners Human Research Committee (the Institutional Review Board) approved that all procedures meet ethical standards for human subject research. Participants have been genotyped using the Illumina Multi-Ethnic Global Array (MEGA) (Illumina, Inc., San Diego, CA) GWAS array. Genetic variants with a high missingness or extreme allele frequencies were removed prior to imputation using the HRCr1.1 reference panel on the Michigan Imputation server. Imputed genotype data in dosage format was used for the analysis. Acne cases were defined based on a diagnosis available on electronic health records (ICD 10 code: L70). Controls were participants not showing an acne ICD code. Participants were excluded if deemed ancestry outliers from the European population or showed high rates of missingness.

GWAS were performed in PLINK v2.00 using a logistic regression adjusting for age, sex, ten genetic principal components and genotype batch as covariates.

7. NETHERLANDS TWIN REGISTER

The Netherlands Twin Register (NTR) is a population-based cohort that includes twins / multiples and their family members from the Netherlands[14]. Phenotype data on acne were collected in multiple surveys. Buccal cells or blood sample for DNA isolation were collected in multiple NTR projects[15,16]. See also: http://www.tweelingenregister.vu.nl/ for more information. All participants gave written informed consent. Studies were approved by the Central Ethics Committee on Research Involving Human Subjects of the VU University Medical Centre, Amsterdam, an Institutional Review Board certified by the U.S. Office of Human Research Protections (IRB number IRB00002991 under Federal-wide Assurance- FWA00017598; IRB/institute codes, NTR 03-180).

7.1 Teenage cohort

The adolescent twins and their family members included in this study were asked to report on their acne when the twins were 14, 16 and 18 years of age. Acne was ascertained by the question “Do you have pimples?” with three answer categories (“no”, “yes, a little”, and “yes, a lot”). Self-reported acne and genotypes were available for 1,819 adolescents and family members at age 14, 2,021 participants at age 16, and 688 participants at age 18. Here, participants were included if self-reported acne and genotyping were available at age 16. Otherwise, the acne measurement of age 14 or 18, depending on availability, was included. Overall, this cross-age dataset included self-reported acne and genotypes of 3,017 individuals from 1,530 families (age range: 10.5 – 35.3 years, mean age = 17.0, SD age = 1.7, 57.2% female). In total, the cross-age dataset included the acne measurements of 2021 individuals (67.0%) as measured when the twins were 16 years of age, of 493 individuals (16.3%) when the twins were 14 years of age, and of 503 individuals (16.7%) when the twins were 18 years of age. The 3-point acne scale was dichotomized by combining the “no” and “yes, a little” answer categories).

7.2 Adult cohort

The adult twins and their family members included in this study completed self-report questionnaires in 2000. Here, acne was ascertained by the question "Do you have pimples?" with two answer categories ("no" [i.e., 'control'], and "yes" [i.e., 'case'])[17]. Self-reported acne and genotypes were available for 3,091 individuals from 1,730 families (age range: 12.0 – 79.0 years, mean age = 30.9, SD age = 11.4, 67.7% females).

7.3 Genotyping and Imputation

For all participants, genotyping was done on several platforms, namely Perlegen Affymetrix, Affymetrix 6.0, Affymetrix Axiom, Illumina Human Quad Bead 660, Illumina Omni 1M, Illumina GSA. Part of the sample was sequenced in the Genome of the Netherlands (GoNL) project[18]. Genotyping of each platform was performed according to the manufacturer's protocols with the calling software as was appropriate at the time. The Single Nucleotide Polymorphisms (SNPs) of the Perlegen-Affymetrix, Illumina Human Quad Bead 660 and the Illumina Omni 1M arrays were typed on older genome builds. Therefore, these were lifted to Build 37 HG19 based on their RSid location in DBSNP 142. Within each platform and on the combined dataset, samples and SNP quality control (QC) was performed. Samples were removed if sex did not match the expected, heterozygosity F statistic was < -0.10 or > 0.10, call rate was < 0.90. SNPs were removed if minor allele frequency (MAF) was <0.01, Hardy-Weinberg Equilibrium (HWE) P-value < 1×10^{-05}, call rate < 0.95, number of Mendel errors < 20[19]. Furthermore, palindromic AT/GC SNPs with MAF between 0.4 and 0.5 were removed to avoid wrong strand alignment to the reference panel. The data of each platform was aligned to the GoNL reference set (V4), and SNPs were removed if they had a difference in allele frequency > 0.10, mismatching alleles, location or strand.

The data of the six platforms were combined into one dataset retaining all QCed SNPs of each platform. For individuals measured on multiple platforms, one platform was chosen in the following order: Affymetrix Axiom, Affymetrix 6.0, Illumina Omni 1M, Illumina Human Quad Bead 660, Illumina GSA, Perlegen-Affymetrix. For the SNPs included in this combined platform, using the PLINK and KING programs, DNA Identity By Descent (IBD) state was estimated for all individual pairs. The IBD estimates were compared to the expected familial relations, and samples were removed if these failed to fit. A similar approach was used for DNA zygosity mismatches. Prior to imputation,

duplicate monozygotic twins (N=3,032), triplets (N=7) and NTR samples from the GONL data (N=364) were removed from the data. Then data were cross-platform imputed using MaCH-Admix against GoNL reference set to predict the missing SNPs in each platform based on haplotypes estimated from Dutch reference panel[20]. After imputation, the GONL samples were re-added to the data, and the second (and third) MZ twin was re-added by duplicating the data of the MZ co-twin included in the imputation. SNPs were removed from the cross-platform imputed dataset if the HWE *P*-value was $< 1 \times 10^{-05}$, Mendel error rate was more than mean + 3SD, R^2 imputation quality metric was < 0.90, if *P*-value for association with a platform was $< 1 \times 10^{-05}$.

Ancestry outliers (non-Dutch ancestry) were defined based on Principal Components Analysis (PCA) by projecting 10 Principal Components (PCs) from 1000G reference set populations on NTR cross-platform imputed data using SMARTPCA. Individuals with PC values located outside of the range of European and/or British populations were defined as outliers. Upon exclusion of outliers, 10 PCs were recomputed for NTR cross-platform imputed data to capture the variation within the Netherlands[21].

Genotype imputation to the HRC 1.1 reference panel was done on the Michigan Imputation server on the cross-platform imputed data[4]. The data were aligned using the PERL based "HRC Imputation preparation and checking" tool (v4.2.5; https://www.well.ox.ac.uk/~wrayner/tools/). The remaining SNPs (1,307,940) were phased with EAGLE for the autosomes and SHAPEIT for chromosome X before imputation using Minimach 3 following the standard imputation procedures of the server[22].

7.4 GWAS

Genome-wide association (GWA) studies were performed with Scalable and Accurate Implementation of Generalized mixed models (SAIGE) v 0.39 to control for unbalanced case-control ratios and account for sample relatedness by the inclusion of a genetic relatedness matrix (GRM;[11]). The NTR cross-platform imputed dataset was used for the calculation of the GRM, and the HRC imputed data (autosomes only) was used for the single-variant association tests. The single-variant association tests employed saddle point approximation to account for case-control imbalance. In the single-variant

association test, the HRC-imputed genotype data was filtered on MAF > 0.0001, Minor Allele Count (MAC) > 1 and R^2 imputation quality metric > 0.60. Covariates included age, sex, genotyping platform and 10 ancestry PCs.

8. QIMR BERGHOFER

8.1 Self Report Cohort

Phenotypic data for acne prevalence was obtained through self-reported questionnaires in the Australian genetics of depression study (AGDS) which comprises ~20,000 participants, of which more than 18,000 have been genotyped[23]. Briefly, the item to ascertain acne was: "*Many people suffer from acne during their lives. How much acne did you have when you were a teenager?*" Participants that responded *Never* were coded as controls, while participants that responded *Moderate* or *Severe* were coded as cases (mild cases excluded). Genotyping was conducted using the Illumina Infinium Global Screening Array platform. Prior to imputation, a common set of high QC markers between the different genotyping batches was obtained. Marker exclusion criteria included: unknown or ambiguous map position and strand alignment in a BLAST search, missingness >5%, p(HWE test)< 10^-6), MAF<1%, GenTrain score <0.6. The Michigan imputation server was used to impute the genotypes using the HRCr1.1 as a reference panel. Individuals were excluded based on high missingness (missing rate > 3%), inconsistent (and unresolvable) sex, or if deemed ancestry outliers from the European population (6 sd deviations from the first two genetic principal components from 1000 Genomes). Imputed genotype dosages were used for the analyses. GWAS was carried out in SAIGE (v0.36.3.3) in R 3.6.1 using a generalised linear mixed model to account for population stratification, cryptic relatedness and unobserved genetic confounding. The GWAS was further adjusted for age, sex, genotyping batch and the first ten principal components.

8.2 Clinical Cohort

Data for this cohort was collected through nurse-rated of acne for 3,817 twins and 674 siblings. The severity of acne was rated by a nurse using a 4-point scale (1 = absent; 2 = mild; 3 = moderate; 4 = severe) on back, face, and chest. Participants were part of the Brisbane Longitudinal Twin Study (BLTS) conducted at the QIMR Berghofer

Medical Research Institute. Within the BLTS study, data is collected on a wide range of biological, psychological, and social traits, as well as environmental exposures. Further details of the recruitment process, data collection, and determination of zygosity can be found in Wright et al.[24]. The measures were taken longitudinally at ages 12 and 14 years and from the face only at age 16. For this analysis, we chose the score that corresponds to the most severe across all sites at all ages to minimize missing data and to allow for individual differences in the time onset of acne; the mean age of the twins was 14.6 (range 12–23, SD 1.4). The individuals in this study were genotyped on the HumanCoreExome-12v1-0_C or IlluminaHuman610WQuad bead chip. Genotypes were imputed to the HRCr1.1 reference panel. Cases were defined as having moderate or severe acne, and controls were defined as having no acne on either the back, face and chest. Mild acne cases were excluded from the analysis. GWAS was carried out in SAIGE as described above. The GWAS was further adjusted for age, sex, genotyping batch and the first ten principal components.

8.3 Independent Sample for PRS Prediction

Self-reported acne diagnosis from the Prospective Imaging of Aging Study (PISA) at QIMR Berghofer was used as our target sample for PRS prediction. PISA is a prospective cohort of healthy Australians at mid to late adulthood and currently consists of approximately 3,000 genotyped individuals who have completed extensive behavioural, psychological and medical questionnaires and cognitive testing and brain imaging[26]. Participants were asked, *"How much acne did you have when you were a teenager?"* to which they could answer *'None', 'Mild', 'Moderate' or 'Severe'.* Only individuals that answered 'Moderate' or 'Severe' were included as cases in our analysis (N=527), and individuals that reported not having acne were considered controls (N=645). Genotyping was previously performed within the recruitment pool as detailed[25,26].

All participants in all QIMR cohorts gave informed written consent; ethical approval was obtained from the QIMR Berghofer Research Ethics Committee.

9. UK BIOBANK

This project used data from the UK Biobank under approved project number 15147. UK Biobank is a prospective study with over 500,000 participants aged 40–69 years when recruited in 2006–2010[27]. The study has collected and continues to collect extensive phenotypic and genotypic detail about its participants, including data from questionnaires, physical measures, sample assays, accelerometry, multimodal imaging, genome-wide genotyping and longitudinal follow-up for a wide range of health-related outcomes. The UK Biobank study was approved by the National Health Service National Research Ethics Service (ref. 11/NW/0382), and all participants provided written informed consent to participate in the UK Biobank study. Information about ethics oversight in the UK Biobank can be found at https://www.ukbiobank.ac.uk/ethics/.

The UK Biobank central team performed genotype calling and imputation. Genotyping was performed using the Affymetrix UK BiLEVE Axiom array on an initial 50,000 participants; the remaining 450,000 participants were genotyped using the Affymetrix UK Biobank Axiom® array that genotyped ~850,000 variants[28].

Based on QC metrics provided by UK Biobank, samples were removed if they were non-European, exhibited gender mismatch, excess relatedness, heterozygosity or missingness > 5%, and individuals that were part of the UKB unrelated White British ancestry subset were extracted, reducing the number of samples from 488,377 to 337,545. We then removed additional individuals with low call rates (<98%) in well-called (>90%) markers, further reducing the number of samples to 336,814.

Genotypes were imputed by the UK Biobank central team using IMPUTE2 software, using UK10K haplotype reference panel merged together with the 1000 Genomes Phase 3 reference panel. Imputed variants included in the analysis were those with imputation R2>0.7 and MAF>0.5%.

Linked primary care diagnosis data were obtained on 8th October 2019. Individuals with acne were identified by searching these records for V2, and CTV3 read codes. All V2 and CTV3 read codes with “acne” in the description were reviewed; codes determined by a dermatologist (C.H.S.) to correspond to a diagnosis of acne vulgaris are listed in the table below. Participants with the selected codes were defined as

cases (N=4,119 individuals in the final genetic dataset), and individuals with any other codes that included the phrase “acne” (e.g. acne rosacea) were excluded from the analysis along with withdrawals (N=5,543). The remaining individuals in the final genetic dataset were defined as controls (N=327,152). GWAS was run using Plink2, using the genotyping array, sex and the first 20 ancestry principal components as covariates.

**Selected V2 read codes**	**Selected CTV3 read codes**
M2610 | Acne vulgaris	M2610 | Acne vulgaris | (Acne vulgaris) or (blackhead) or (comedo)
M261. | Other acne	M261. | Other acne
M261X | Acne, unspecified	
M261A | Pustular acne	M261A | Pustular acne
M261z | Other acne NOS	M261z | Other acne NOS
M2616 | Cystic acne	M2616 | Cystic acne vulgaris | Cystic acne
Myu6F | Acne, unspecified	Myu6F | Acne, unspecified
Myu68 | Other acne	Myu68 | Other acne
2FG5. | Acne scar	X75uJ | Acne scar | Post-acne scarring
	XE1BO | Acne vulgaris
	X50AA | Acne excoriee | Acne excoriee des jeunes filles | Excoriated acne
	X50A4 | Acne
	X50A6 | Comedonal acne
	X50A7 | Nodulocystic acne
	XE1Cy | Acne vulgaris: [comedo] | Acne vulgaris | Pustular acne | Vulgaris - acne
	m7... | Acne - oral preparations

2. COHORT ACKNOWLEDGMENTS AND FUNDING

a. ALSPAC

We are extremely grateful to all the families who took part in this study, the midwives for their help in recruiting them, and the whole ALSPAC team, which includes interviewers, computer and laboratory technicians, clerical workers, research scientists, volunteers, managers, receptionists and nurses.

The UK Medical Research Council and Wellcome (Grant ref: 217065/Z/19/Z) and the University of Bristol provide core support for ALSPAC. GWAS data was generated by Sample Logistics and Genotyping Facilities at Wellcome Sanger Institute and LabCorp (Laboratory Corporation of America) using support from 23andMe. This publication is the work of the authors and Josine Min will serve as guarantors for the contents of this paper.

b. FINNGEN

We want to acknowledge the participants and investigators of the FinnGen study.

c. HARVARD

We are indebted to the participants in the NHS, NHS2, and HPFS for their dedication to this research. We thank the Channing Division of Network Medicine in Brigham and Women’s Hospital for their help. This work is supported by National Institutes of Health grants UM1 CA186107, U01 CA176726, and U01 CA167552. The content is solely the responsibility of the authors and does not necessarily represent the official views of the National Institutes of Health.

d. HUNT

The Trøndelag Health Study (The HUNT Study) is a collaboration between HUNT Research Centre (Faculty of Medicine and Health Sciences, NTNU, Norwegian University of Science and Technology), Trøndelag County Council, Central Norway

Regional Health Authority, and the Norwegian Institute of Public Health. LT, KH and ML work in a research unit funded by Stiftelsen Kristian Gerhard Jebsen; Faculty of Medicine and Health Sciences, NTNU, Norwegian University of Science and Technology; The Liaison Committee for education, research and innovation in Central Norway; the Joint Research Committee between St. Olavs Hospital (Trondheim, Norway) and the Faculty of Medicine and Health Sciences, NTNU, Norwegian University of Science and Technology. ML is supported by grants from the Liaison Committee for education, research and innovation in Central Norway.

e. King's College London (KCL)

We thank the funders and contributors to the previous two KCL acne GWAS studies, Petridis et al. (2018): Christos Petridis, Alexander A. Navarini, David Baudry, Michael Duckworth, Michael H. Allen, Charles J. Curtis, Sang Hyuck Lee, A. David A. Burden, Alison Layton, Veronique Bataille, Andrew E. Pink, The Acne Genetic Study Group, Isabelle Carlavan, Johannes J. Voegel, Timothy D. Spector, Richard C. Trembath and John A. McGrath; and Navarini et al. (2014): Michael Weale, Jo Knight, Pascale Reiniche, Robert Pleass, Daniel Creamer, John English, Stephanie Munn, Shernaz Walton, Carolyn Willis and Sophie Déret. We thank Alka Saxena; Ulrike Blume-Peytavi; Leaca Crawford; Jana Estafan; Darren Geoghegan; Dan Glass; Alison Gosh; Naomi Hare; Helen Holmes; Karen Markwell; Eleanor Mallon; Philippe Martel; Carine Marty; Corinne Ménigot; Efterpi Papouli; Anne Thompson; Kate Thornberry; Bianca Tobin, and the participating patients and supporting staff in all the study centres.

Support was provided by the National Institute for Health Research (NIHR), through the Dermatology Clinical Research Network; the NIHR Biomedical Research Centre based at Guy's and St Thomas' NHS Foundation Trust and King's College London; Galderma, Bruno Bloch, Promedica Foundation and HSM-2 canton of Zurich, British Skin Foundation; Medical Research Council/British Association of Dermatologists/British Skin Foundation Clinical Research Training Fellowship; the NIHR Maudsley Biomedical Research Centre at South London and Maudsley NHS Foundation Trust and King's College London; capital equipment funding from the Maudsley Charity (Grant Ref. 980) and Guy's and St Thomas' Charity (Grant Ref. STR130505).

f. MASS GENERAL BRIGHAM BIOBANK (PARTNERS BIOBANK)

We want to acknowledge Partners HealthCare Biobank for providing samples, genomic data, and health information data. XD is supported by American Parkinson's Disease Association, ASAP, and NIH U01.

g. Netherlands Twin Register

We thank all twins and family members for their participation. Analyses were supported by the Netherlands Organization for Scientific Research: Netherlands Twin Registry Repository: researching the interplay between genome and environment (480-15-001/674); the European Union Seventh Framework Program (FP7/2007-2013): ACTION Consortium (Aggression in Children: Unravelling gene-environment interplay to inform Treatment and InterventiON strategies; grant number 602768). Genotyping was made possible by grants from NWO/SPI 56-464-14192, Genetic Association Information Network (GAIN) of the Foundation for the National Institutes of Health, Rutgers University Cell and DNA Repository (NIMH U24 MH068457-06), the Avera Institute, Sioux Falls (USA) and the National Institutes of Health (NIH R01 HD042157-01A1, MH081802, Grand Opportunity grants 1RC2 MH089951 and 1RC2 MH089995) and European Research Council (ERC-230374). DIB acknowledges her KNAW Academy Professor Award (PAH/6635). MB is supported by an ERC consolidator grant (WELL-BEING 771057 PI Bartels).

h. QIMR Berghofer

We are indebted to all participants for giving their time to contribute to these studies. Additionally, we wish to thank all the people who helped in the conception, implementation, media campaign and data cleaning. In particular, we would like to thank our research nurses - including Ann Eldridge and Marleen Grace, who were responsible for collecting the acne data in our young twin cohort under the supervision of Dr. Margie Wright. We would like to thank Scott Gordon who curated all the genotyping data that was used in this project. Data collection for the Australian

Genetics of Depression Study (self-report cohort) was possible, thanks to funding from the National Health and Medical Research Council (NHMRC) of Australia grant 1086683. This work was further supported by NHMRC grants 1145645, 1078901 and 1087889. PISA is funded by a NHMRC Boosting Dementia Research Initiative grant, APP1095227, to NGM and others. MKL is supported by a Boosting Dementia Leadership Fellowship (APP1140441). MER thanks support of the NHMRC and Australian Research Council (GNT1102821). BLM is grateful for support from Queensland University of Technology through a QUT Postgraduate Research Scholarship.

i. UK BIOBANK

We would like to acknowledge all UK Biobank participants for dedicating their free time to participate. UK Biobank is generously supported by its founding funders the Wellcome Trust and UK Medical Research Council, as well as the Department of Health, Scottish Government, the Northwest Regional Development Agency, British Heart Foundation and Cancer Research UK. The organisation has over 150 dedicated members of staff, based in multiple locations across the UK.

This research has been conducted using the UK Biobank Resource (approved project 15147). N.D. is funded by Health Data Research UK (MR/S003126/1), which is funded by the UK Medical Research Council, Engineering and Physical Sciences Research Council; Economic and Social Research Council; Department of Health & Social Care (England); Chief Scientist Office of the Scottish Government Health and Social Care Directorates; Health and Social Care Research and Development Division (Welsh Government); Public Health Agency (Northern Ireland); British Heart Foundation; and Wellcome.

3. SUPPLEMENTARY FIGURES

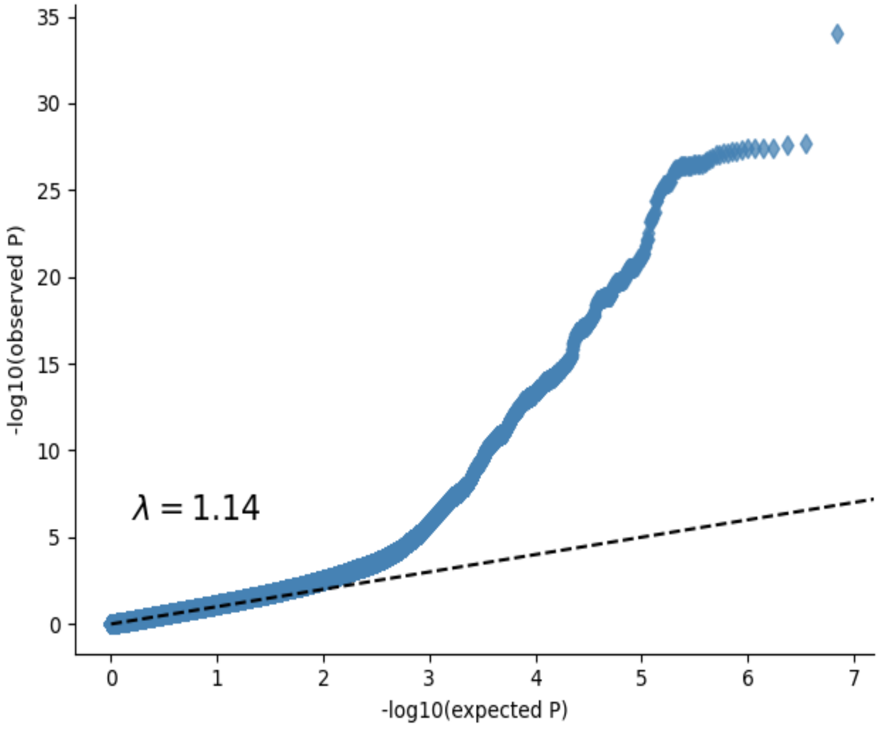

Supplementary Figure 1: Quantile-quantile (QQ) plot of -log10(*P*-values) from the fixed-effects meta-analysis. Experimentally observed *P*-values (two-tailed *Z*-test, not adjusted for multiple comparison) for 7,072,770 autosomal SNPs are plotted on the y-axis (blue) compared to the expected *P*-values of a null distribution (x-axis). Deviation from the expected *P*-values under the null hypothesis is presented using the standard genomic inflation factor lambda (λ).

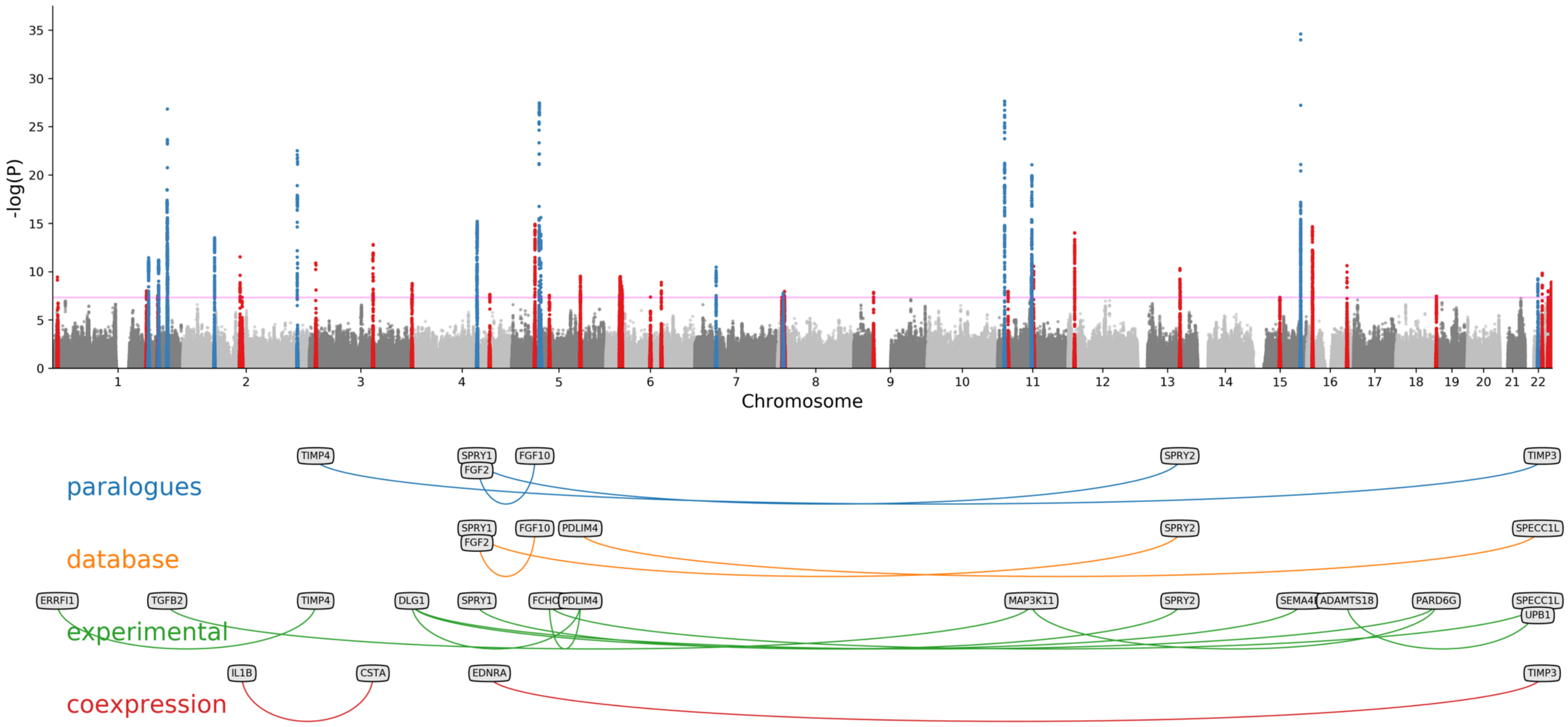

Supplementary Figure 2: Manhattan plot from Figure 1 annotated with links between genes from Table 1 to indicate functional similarities. Links were drawn between genes within the same Ensembl paralogue family, or if they were associated within STRINGdb due to co-expression, experimentally determined protein-protein interactions or co-existence in curated pathways.

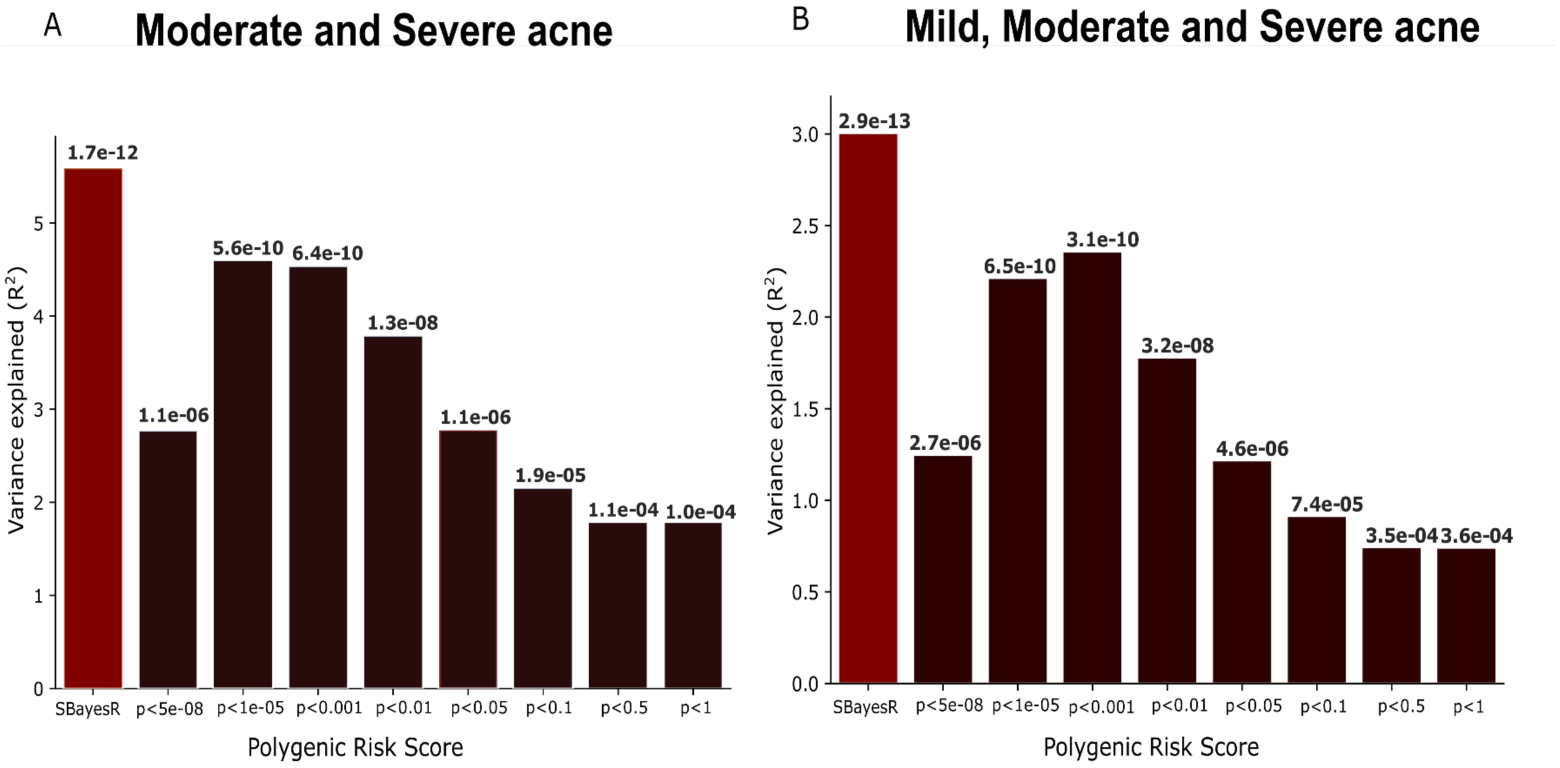

Supplementary Figure 3: A. Polygenic Risk Scores for acne explain up to 5.6% of variance in moderate to severe acne reported in an independent sample. B. Acne PRS explain up to 3% of variance in mild, moderate or severe acne cases. A partial R^2 (y-axis) was calculated using a mixed model regression with the acne PRS (x-axis) as a predictor variable while accounting for sex, age and the first ten genetic principal components. Significance values were calculated using a two-tailed Student's *t*-test. Multiple testing correction was applied using an FDR<5% threshold.

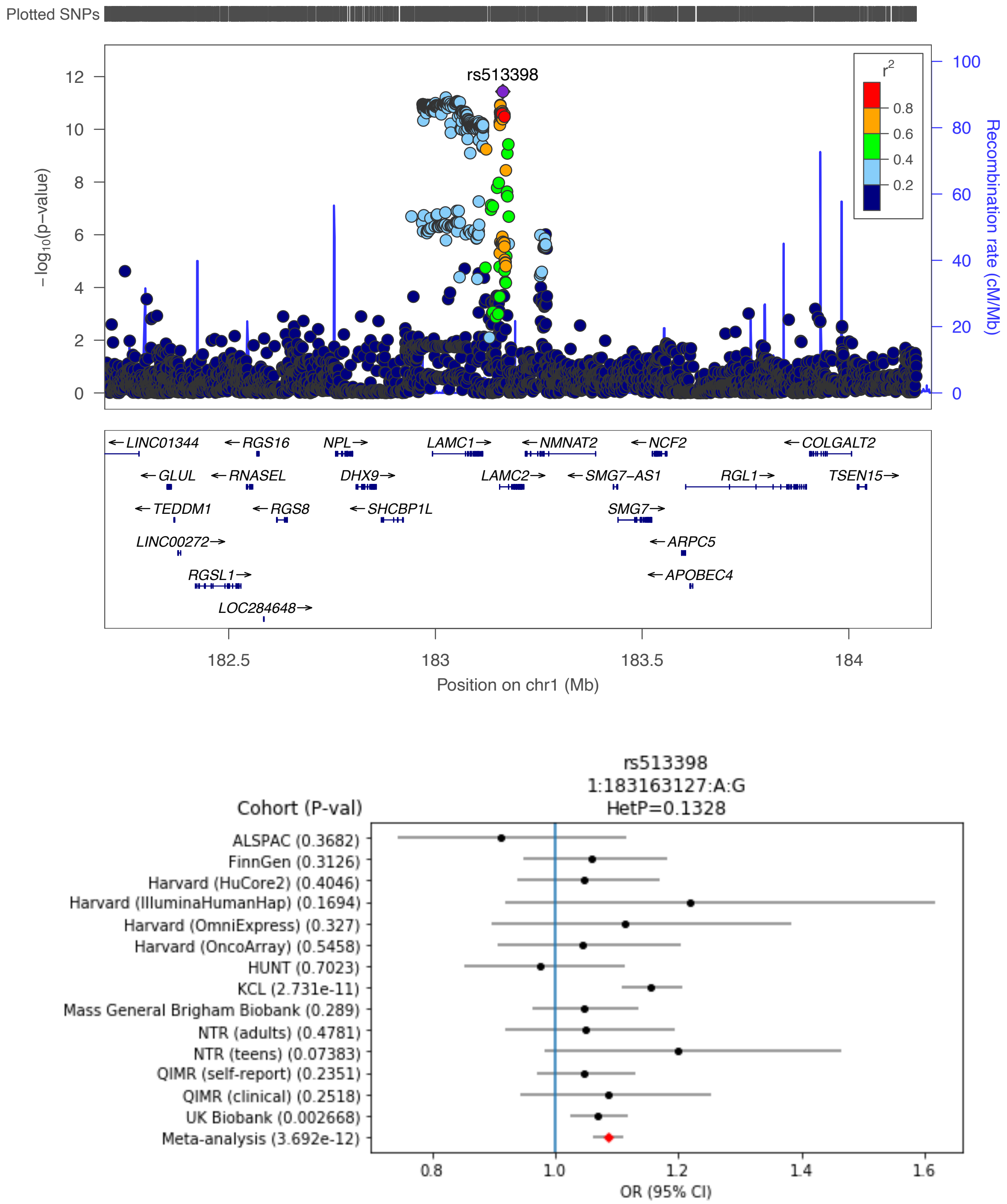

Supplementary Figure 4(i): Upper: meta-analysis locuszoom plot for established 1q25.3 locus. Lower: forest plot for established 1q25.3 locus, presented as odds ratio +/- 95% confidence intervals. Association *P*-values annotated for meta-analysis (two-sided *Z*-test, not adjusted for multiple testing) and each cohort (calculated as detailed in Supplementary Note).

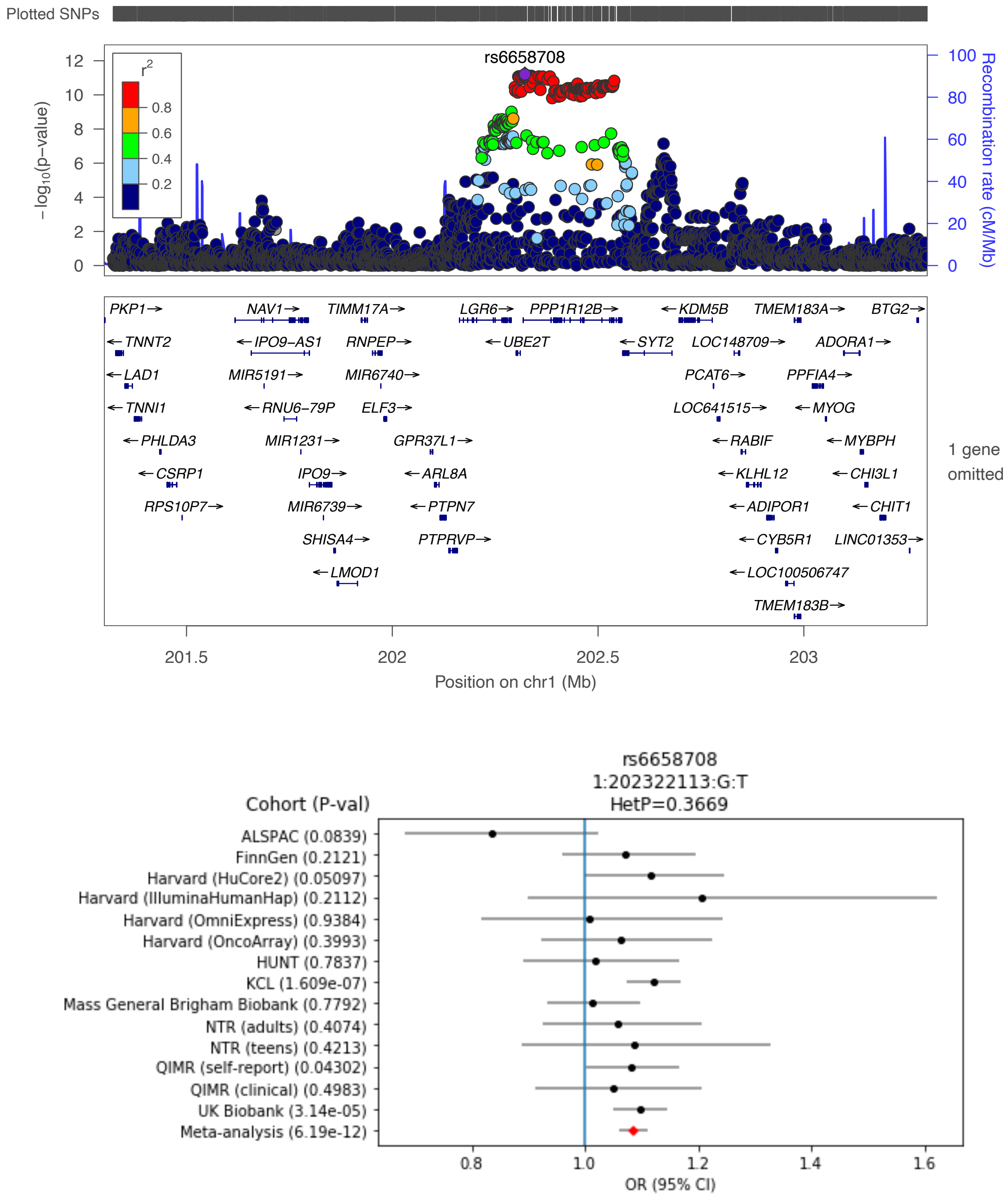

Supplementary Figure 4(ii): Upper: meta-analysis locuszoom plot for established 1q32.1 locus. Lower: forest plot for established 1q32.1 locus, presented as odds ratio +/- 95% confidence intervals. Association *P*-values annotated for meta-analysis (two-sided *Z*-test, not adjusted for multiple testing) and each cohort (calculated as detailed in Supplementary Note).

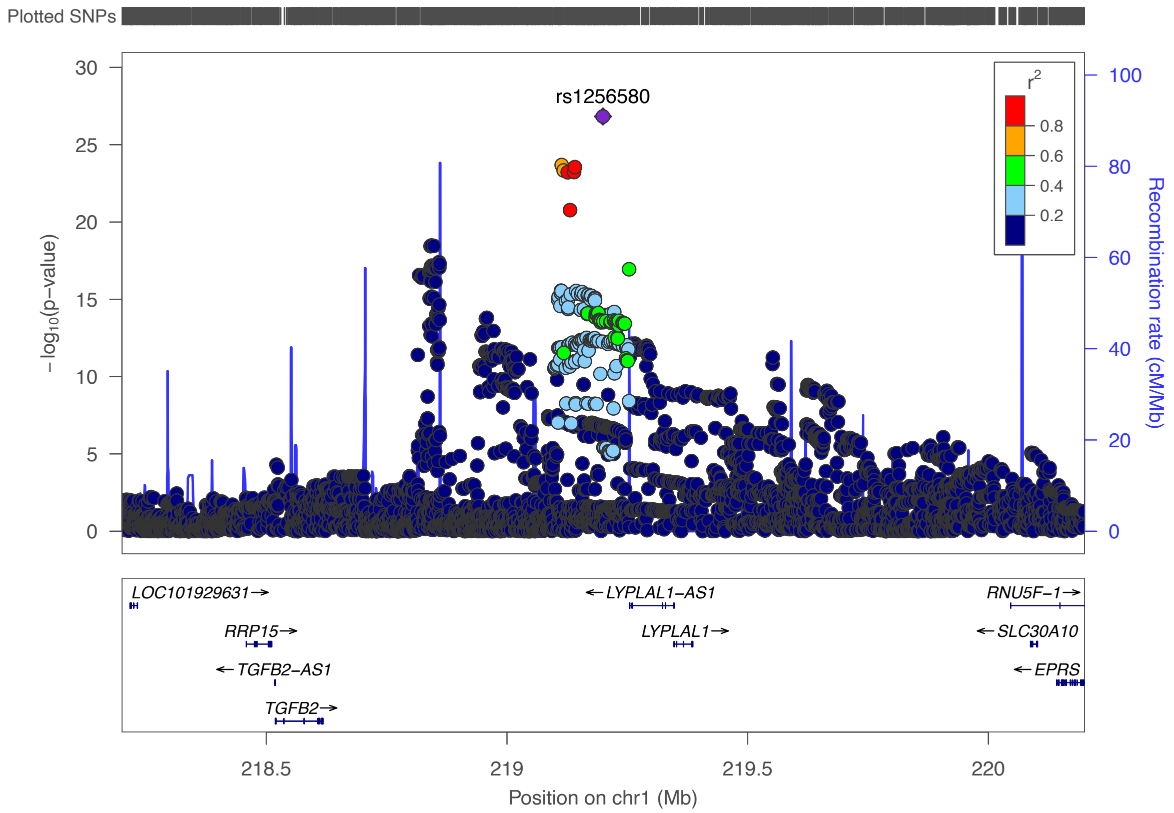

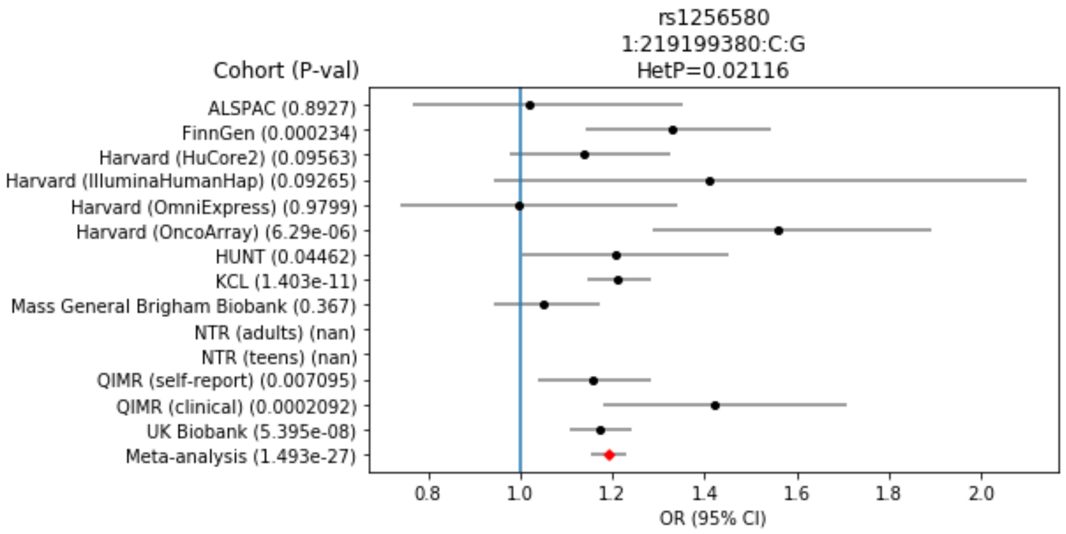

Supplementary Figure 4(iii): Upper: meta-analysis locuszoom plot for established 1q41 locus. Lower: forest plot for established 1q41 locus, presented as odds ratio +/- 95% confidence intervals. Association *P*-values annotated for meta-analysis (two-sided *Z*-test, not adjusted for multiple testing) and each cohort (calculated as detailed in Supplementary Note).

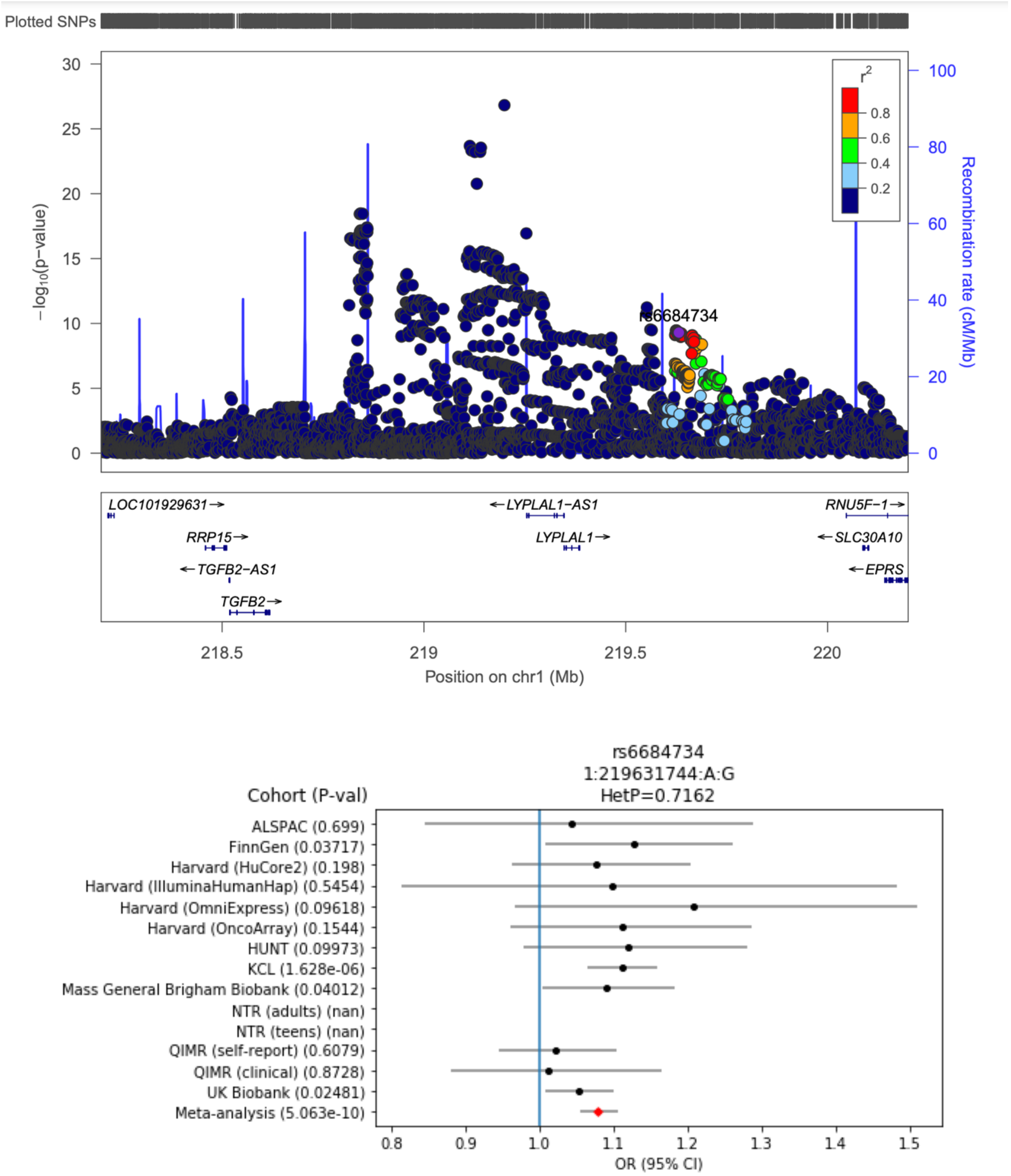

Supplementary Figure 4(iv): Upper: meta-analysis locuszoom plot for second independent signal at established 1q41 locus. Lower: forest plot for second independent signal at established 1q41 locus, presented as odds ratio +/- 95% confidence intervals. Association *P*-values annotated for meta-analysis (two-sided *Z*-test, not adjusted for multiple testing) and each cohort (calculated as detailed in Supplementary Note).

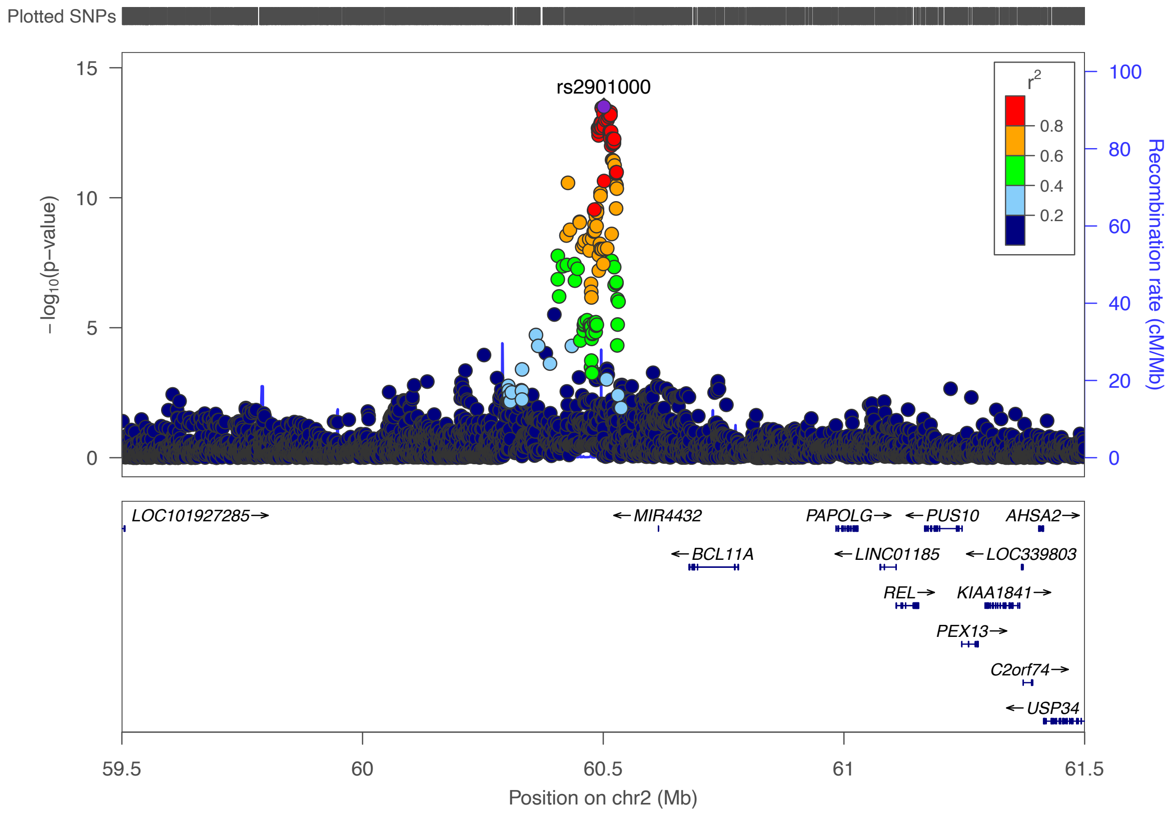

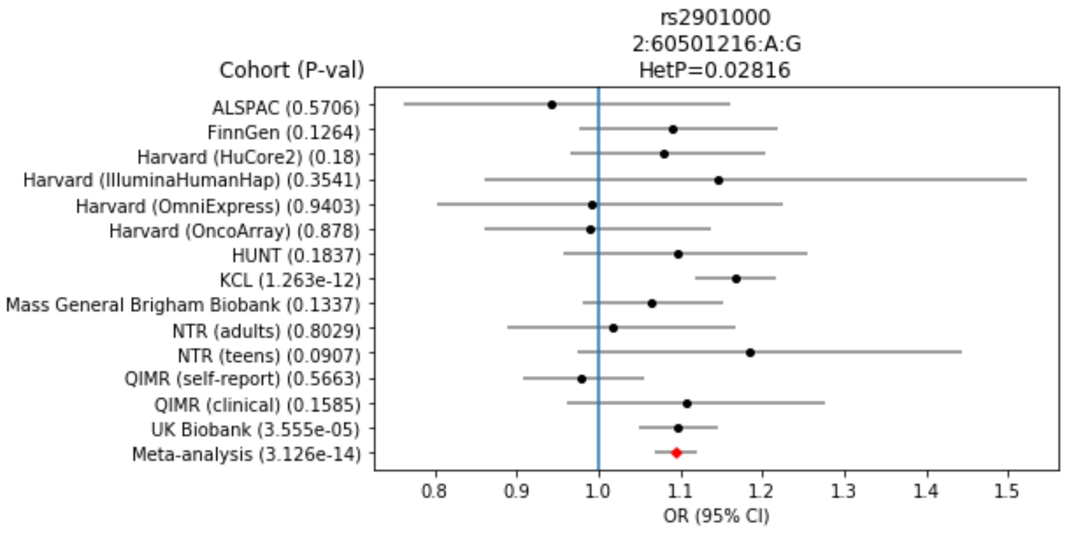

Supplementary Figure 4(v): Upper: meta-analysis locuszoom plot for established 2p16.1 locus. Lower: forest plot for established 2p16.1 locus, presented as odds ratio +/- 95% confidence intervals. Association *P*-values annotated for meta-analysis (two-sided *Z*-test, not adjusted for multiple testing) and each cohort (calculated as detailed in Supplementary Note).

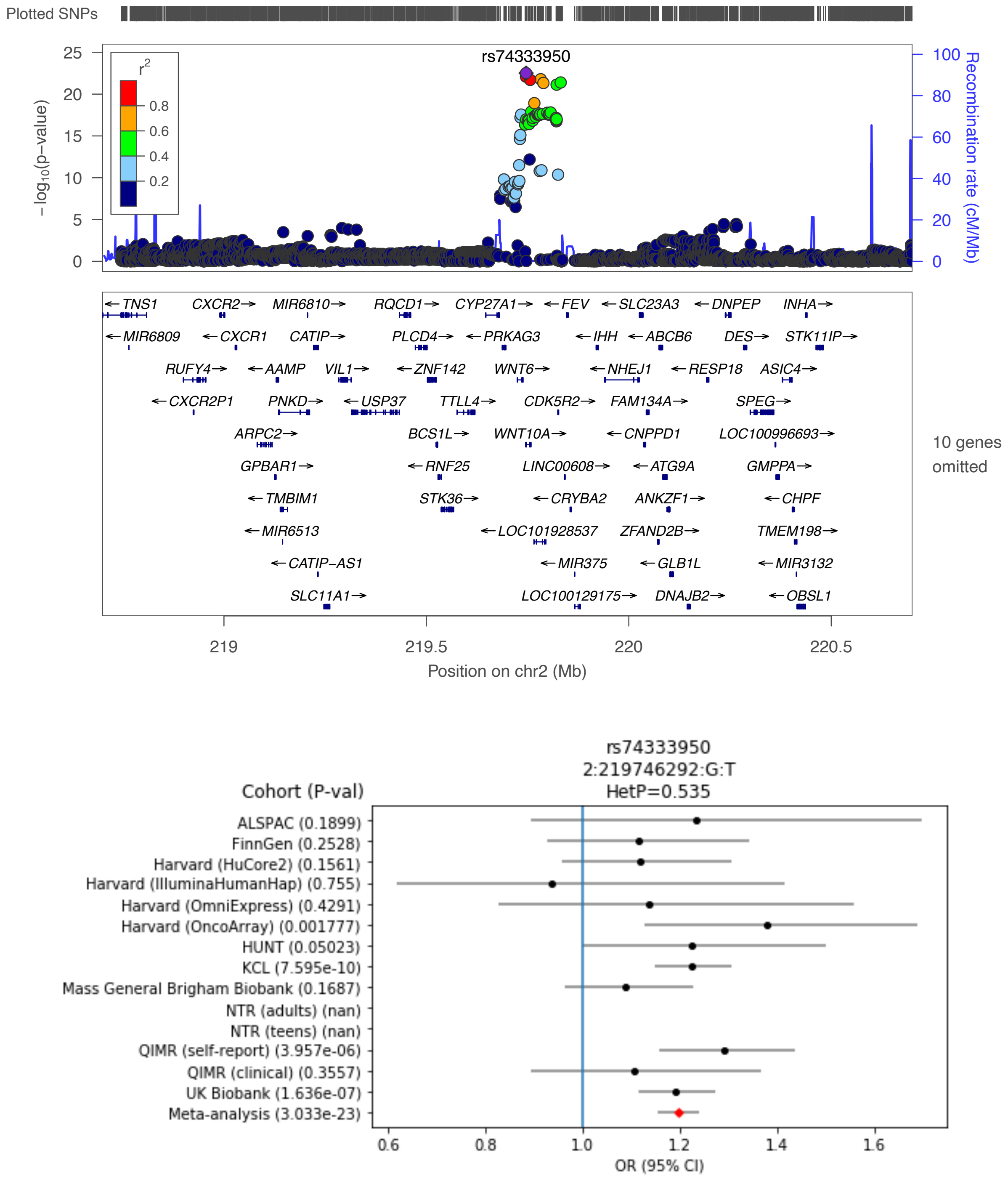

Supplementary Figure 4(vi): Upper: meta-analysis locuszoom plot for established 2q35 locus. Lower: forest plot for established 2q35 locus, presented as odds ratio +/- 95% confidence intervals. Association *P*-values annotated for meta-analysis (two-sided *Z*-test, not adjusted for multiple testing) and each cohort (calculated as detailed in Supplementary Note).

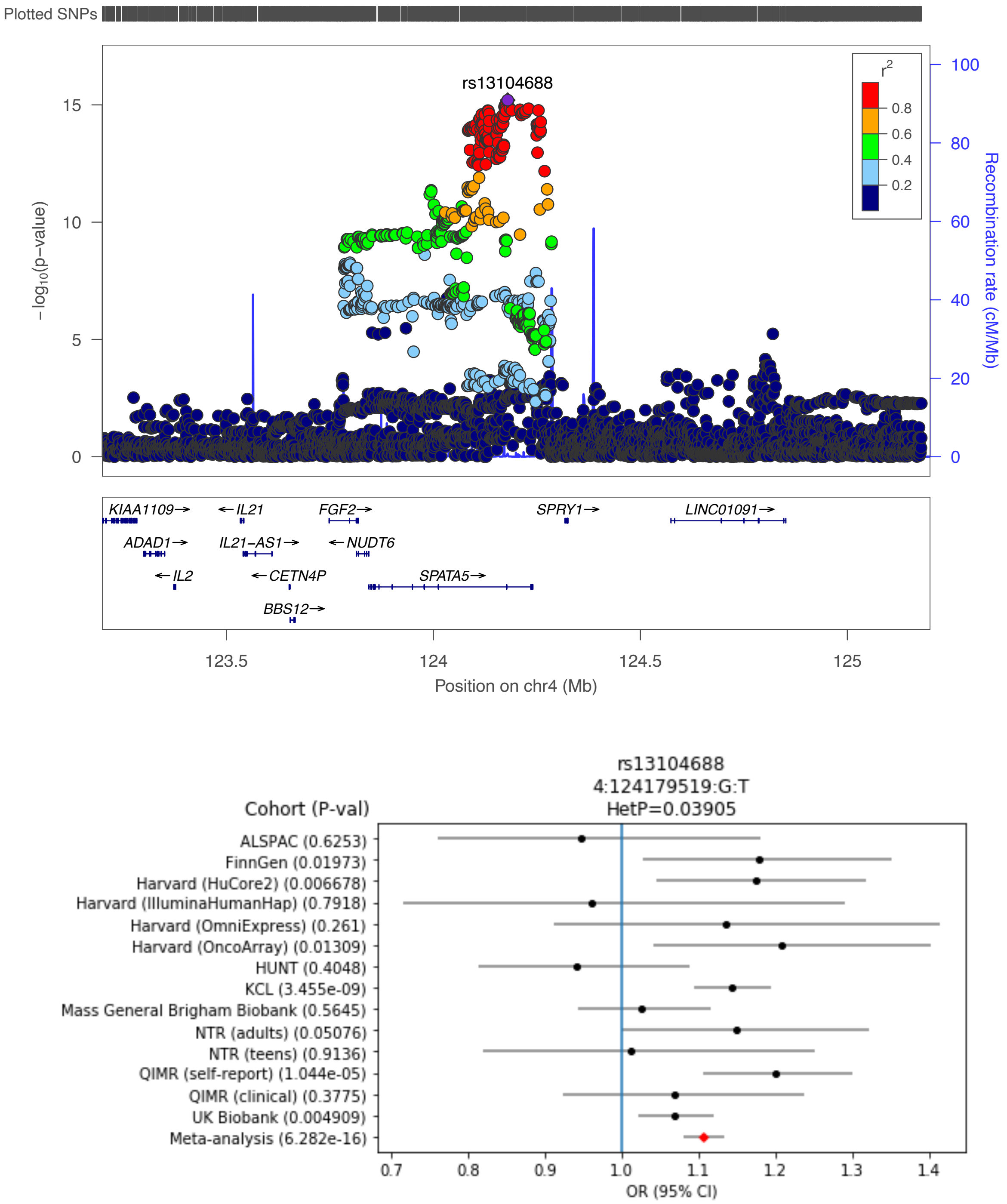

Supplementary Figure 4(vii): Upper: meta-analysis locuszoom plot for established 4q28.1 locus. Lower: forest plot for established 4q28.1 locus, presented as odds ratio +/- 95% confidence intervals. Association *P*-values annotated for meta-analysis (two-sided *Z*-test, not adjusted for multiple testing) and each cohort (calculated as detailed in Supplementary Note).

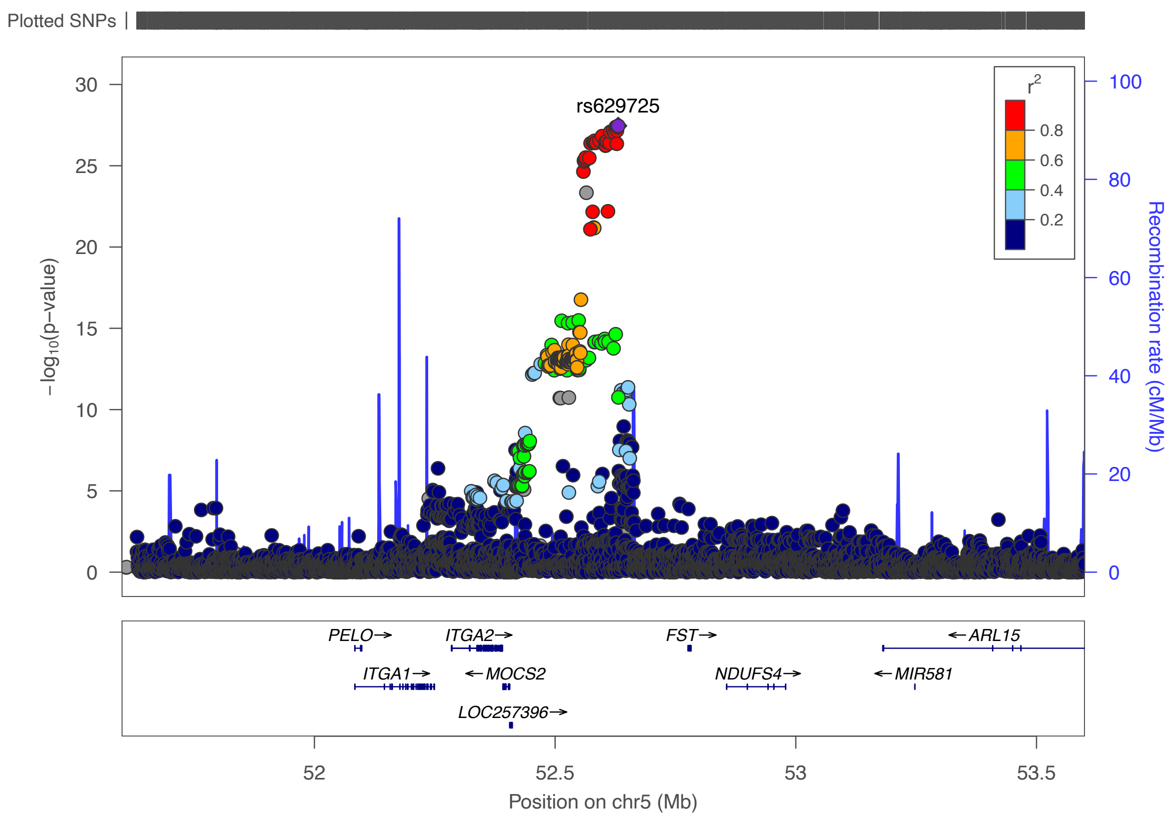

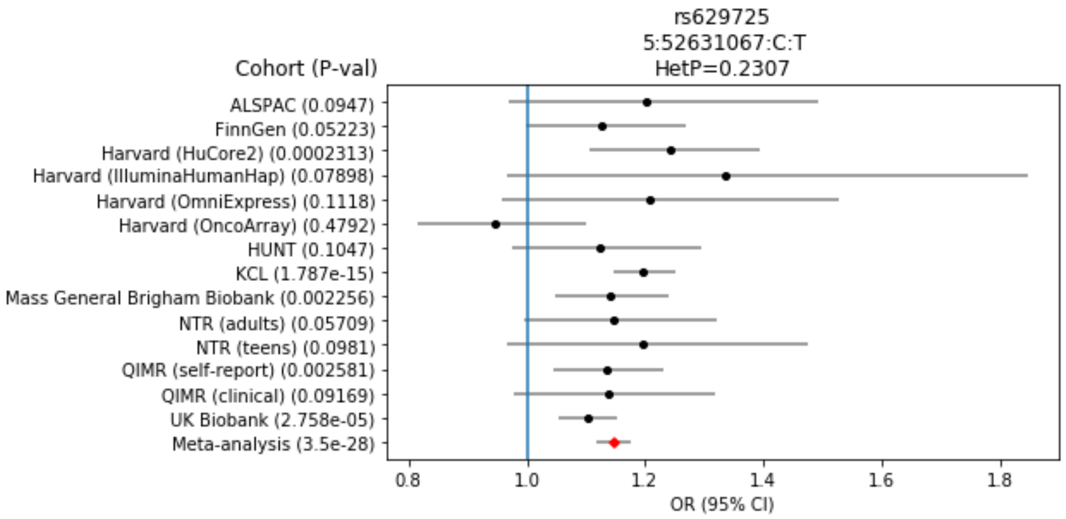

Supplementary Figure 4(viii): Upper: meta-analysis locuszoom plot for established 5q11.2 locus. Lower: forest plot for established 5q11.2 locus, presented as odds ratio +/- 95% confidence intervals. Association *P*-values annotated for meta-analysis (two-sided *Z*-test, not adjusted for multiple testing) and each cohort (calculated as detailed in Supplementary Note).

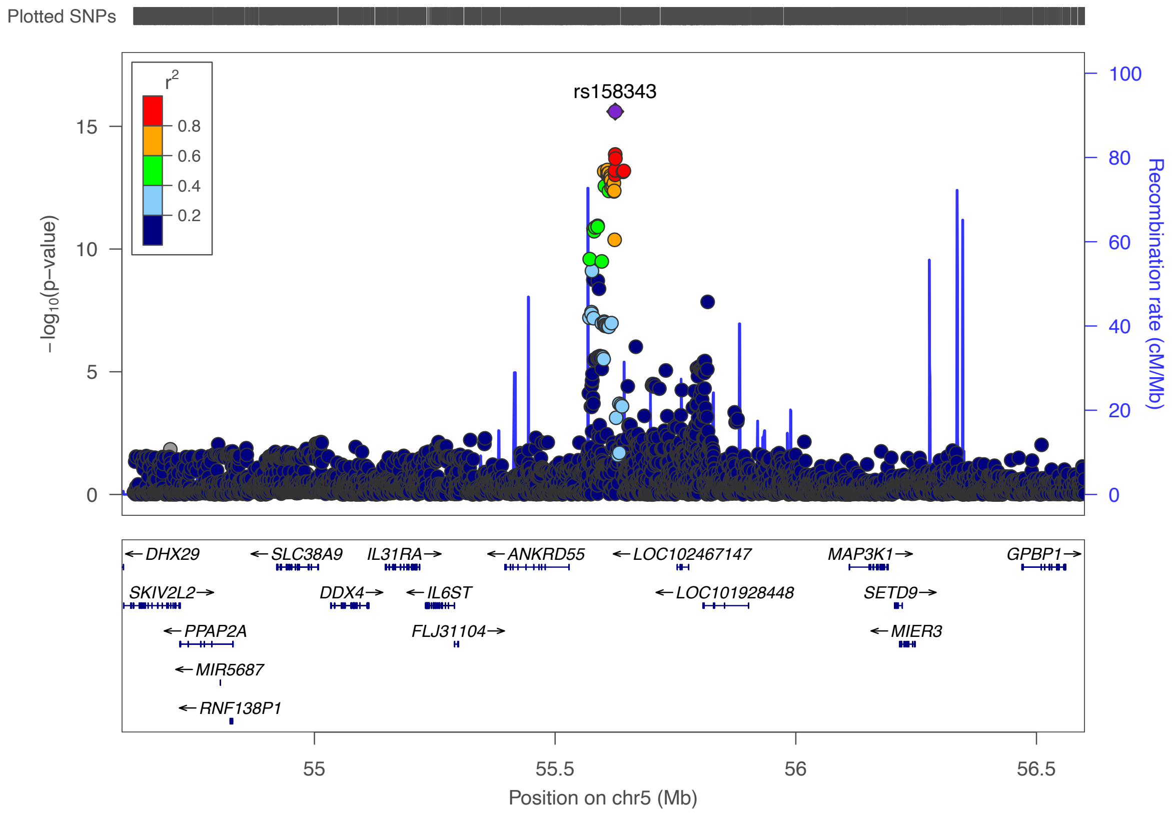

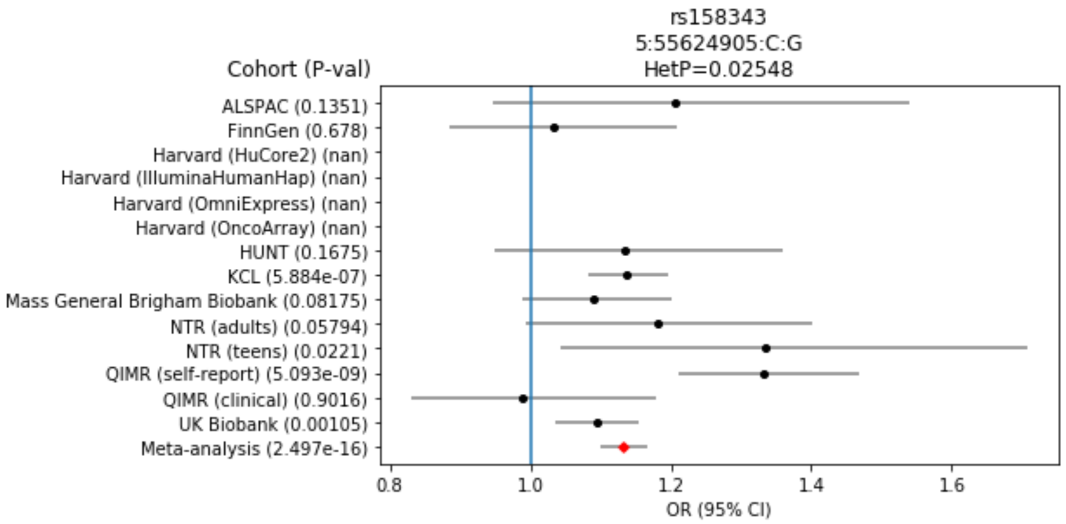

Supplementary Figure 4(ix): Upper: meta-analysis locuszoom plot for established 5q11.2 locus. Lower: forest plot for established 5q11.2 locus, presented as odds ratio +/- 95% confidence intervals. Association *P*-values annotated for meta-analysis (two-sided *Z*-test, not adjusted for multiple testing) and each cohort (calculated as detailed in Supplementary Note).

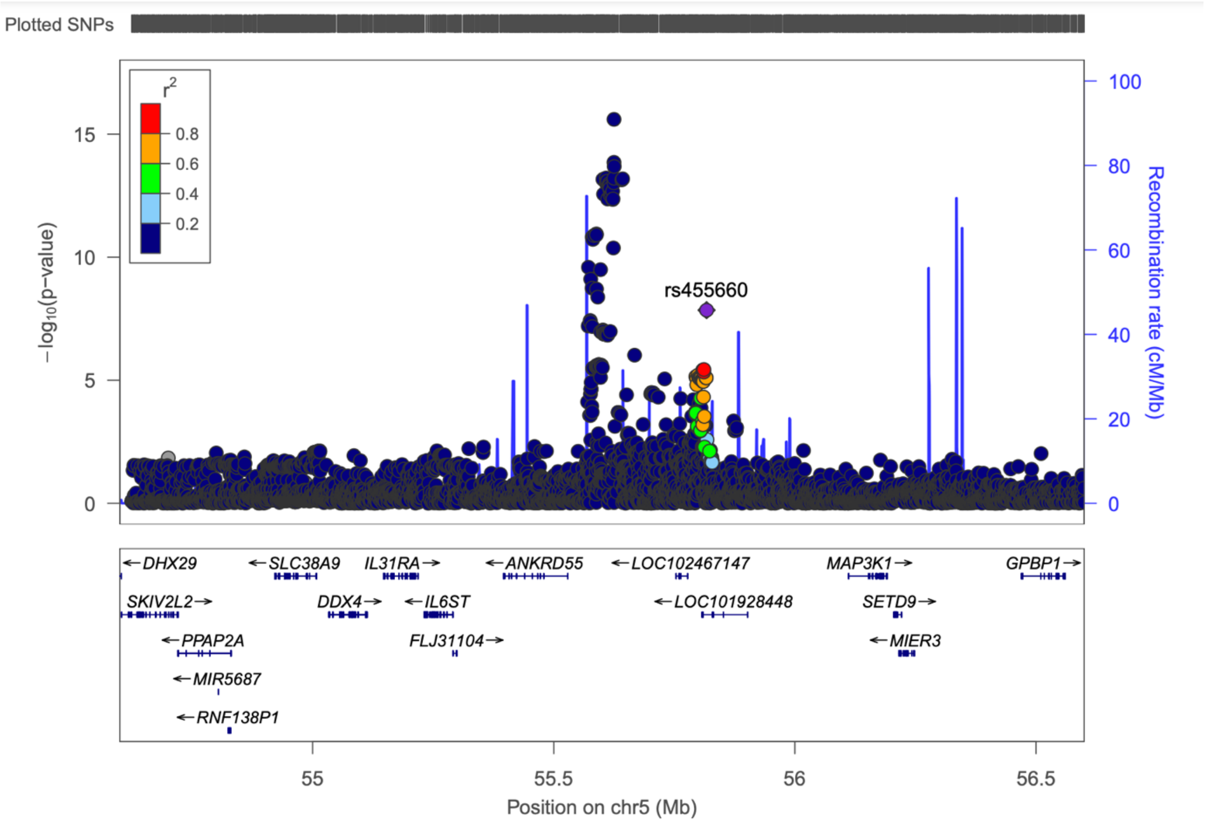

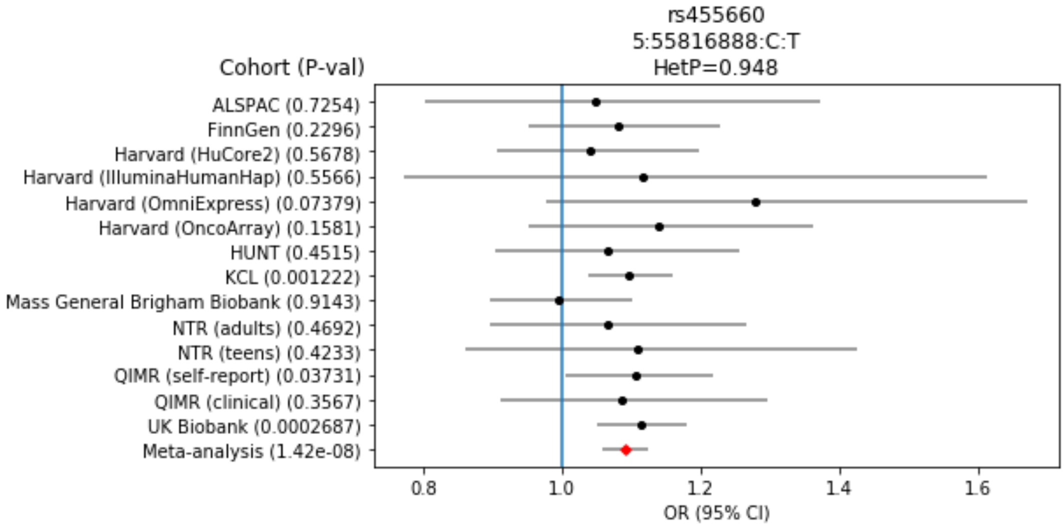

Supplementary Figure 4(x): Upper: meta-analysis locuszoom plot for second independent signal at established 5q11.2 locus. Lower: forest plot for second independent signal at established 5q11.2 locus, presented as odds ratio +/- 95% confidence intervals. Association *P*-values annotated for meta-analysis (two-sided *Z*-test, not adjusted for multiple testing) and each cohort (calculated as detailed in Supplementary Note).

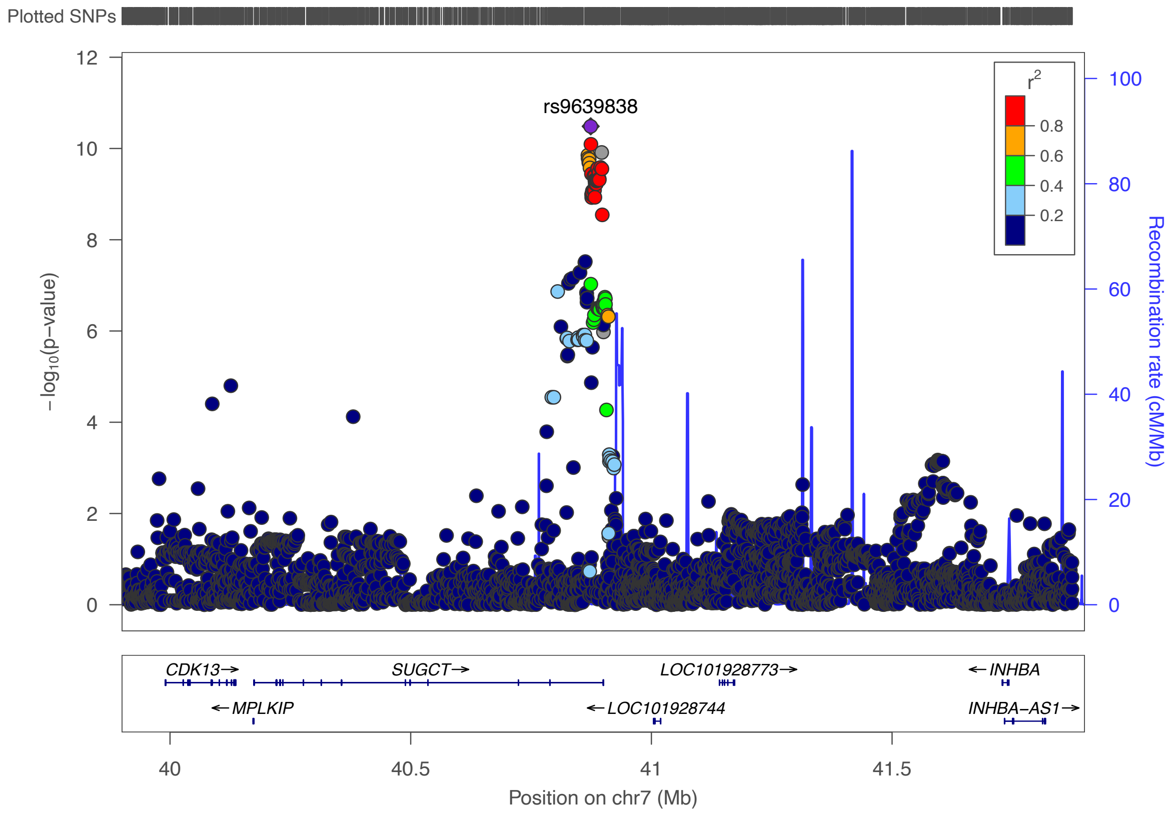

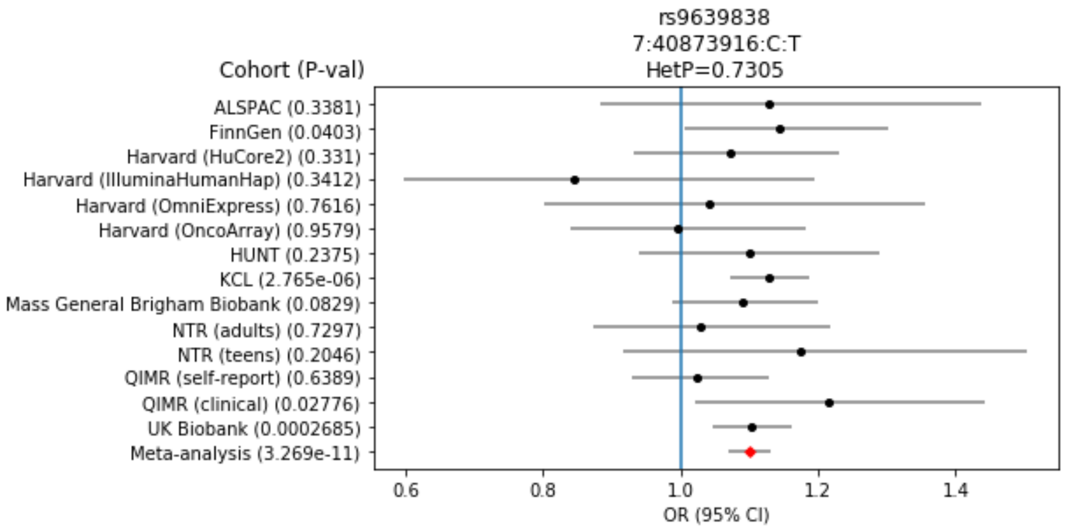

Supplementary Figure 4(xi): Upper: meta-analysis locuszoom plot for established 7p14.1 locus. Lower: forest plot for established 7p14.1 locus, presented as odds ratio +/- 95% confidence intervals. Association *P*-values annotated for meta-analysis (two-sided *Z*-test, not adjusted for multiple testing) and each cohort (calculated as detailed in Supplementary Note).

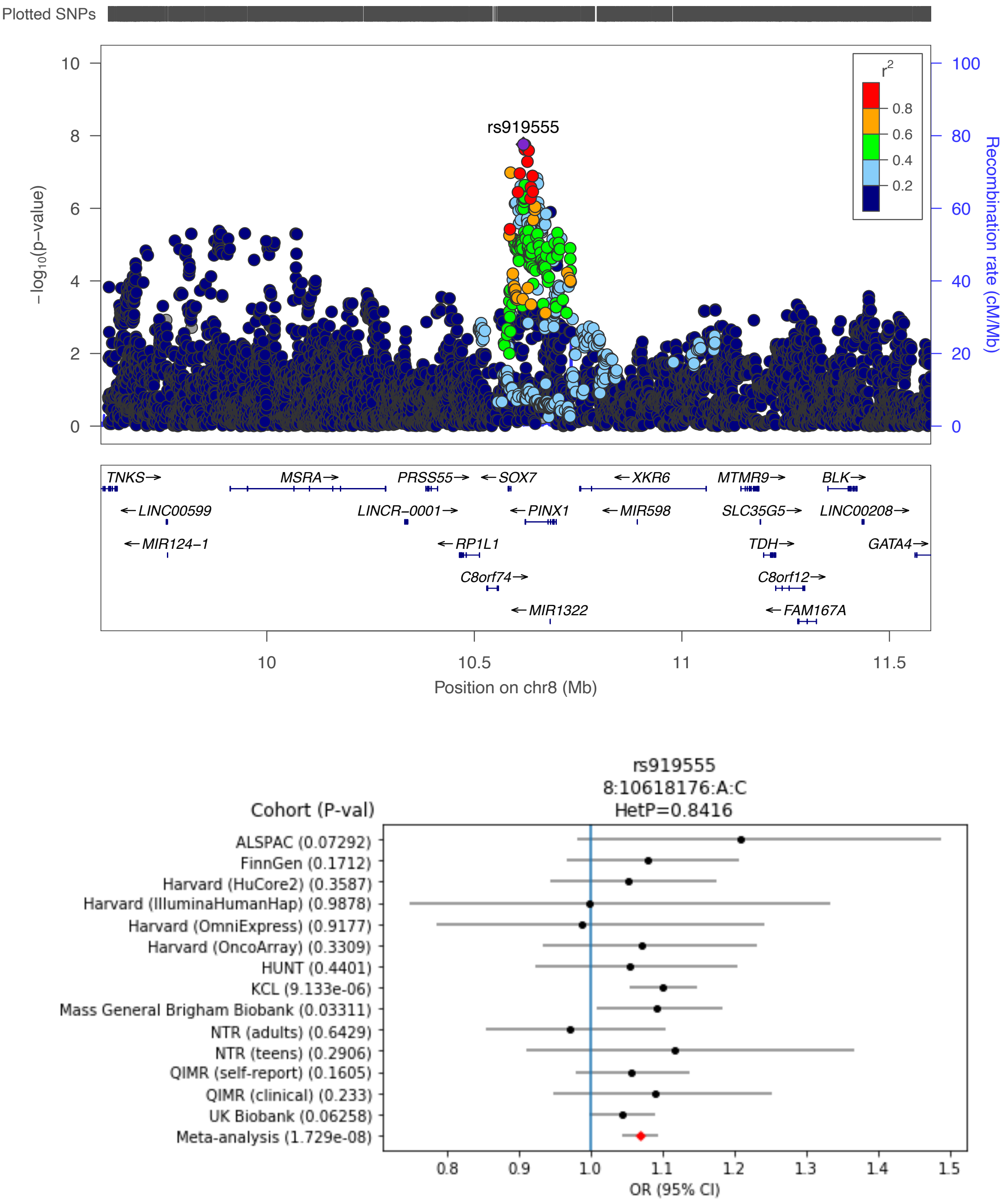

Supplementary Figure 4(xii): Upper: meta-analysis locuszoom plot for established 8p23.1 locus. Lower: forest plot for established 8p23.1 locus, presented as odds ratio +/- 95% confidence intervals. Association *P*-values annotated for meta-analysis (two-sided *Z*-test, not adjusted for multiple testing) and each cohort (calculated as detailed in Supplementary Note).

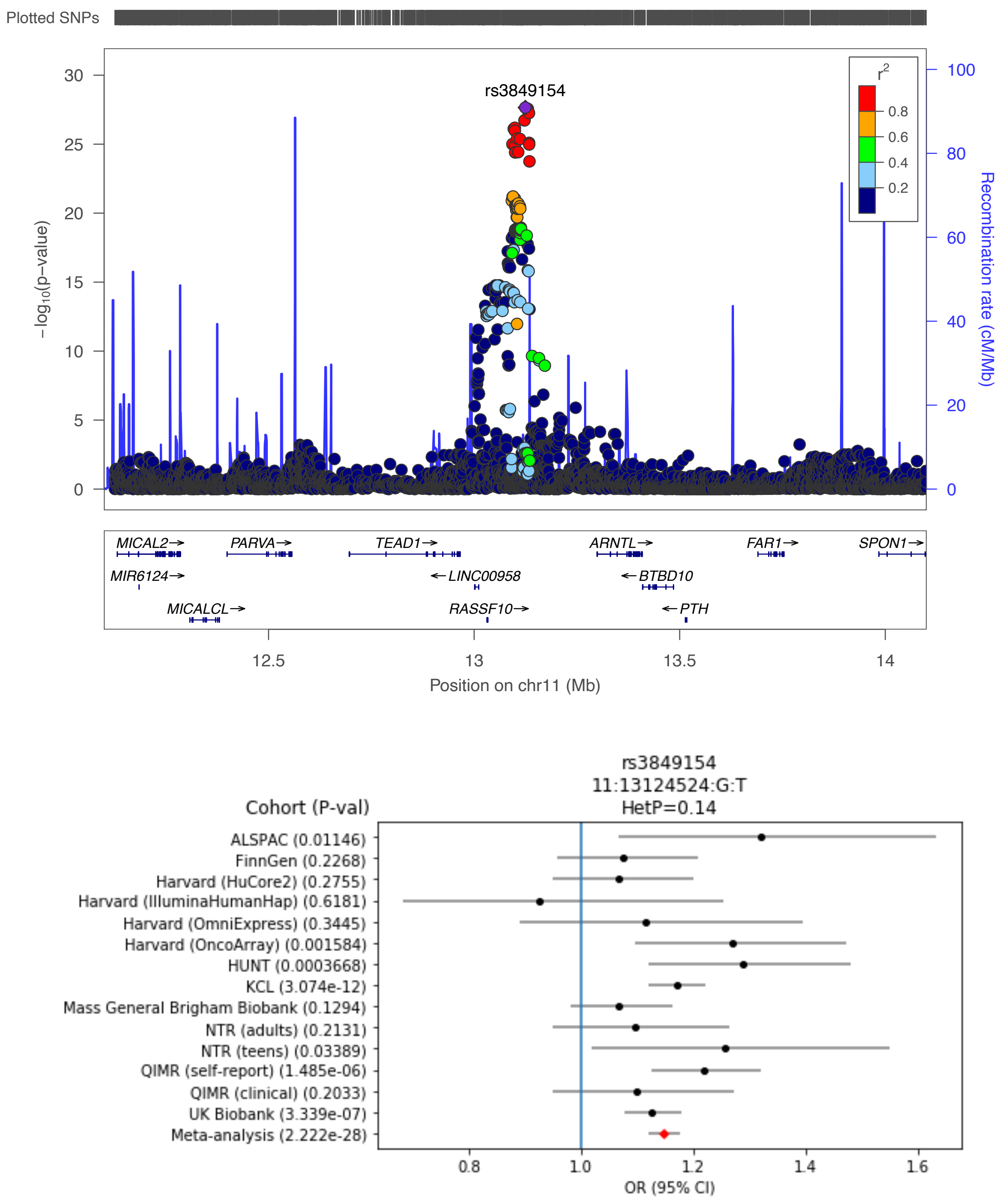

Supplementary Figure 4(xiii): Upper: meta-analysis locuszoom plot for established 11p15.2 locus. Lower: forest plot for established 11p15.2 locus, presented as odds ratio +/- 95% confidence intervals. Association *P*-values annotated for meta-analysis (two-sided *Z*-test, not adjusted for multiple testing) and each cohort (calculated as detailed in Supplementary Note).

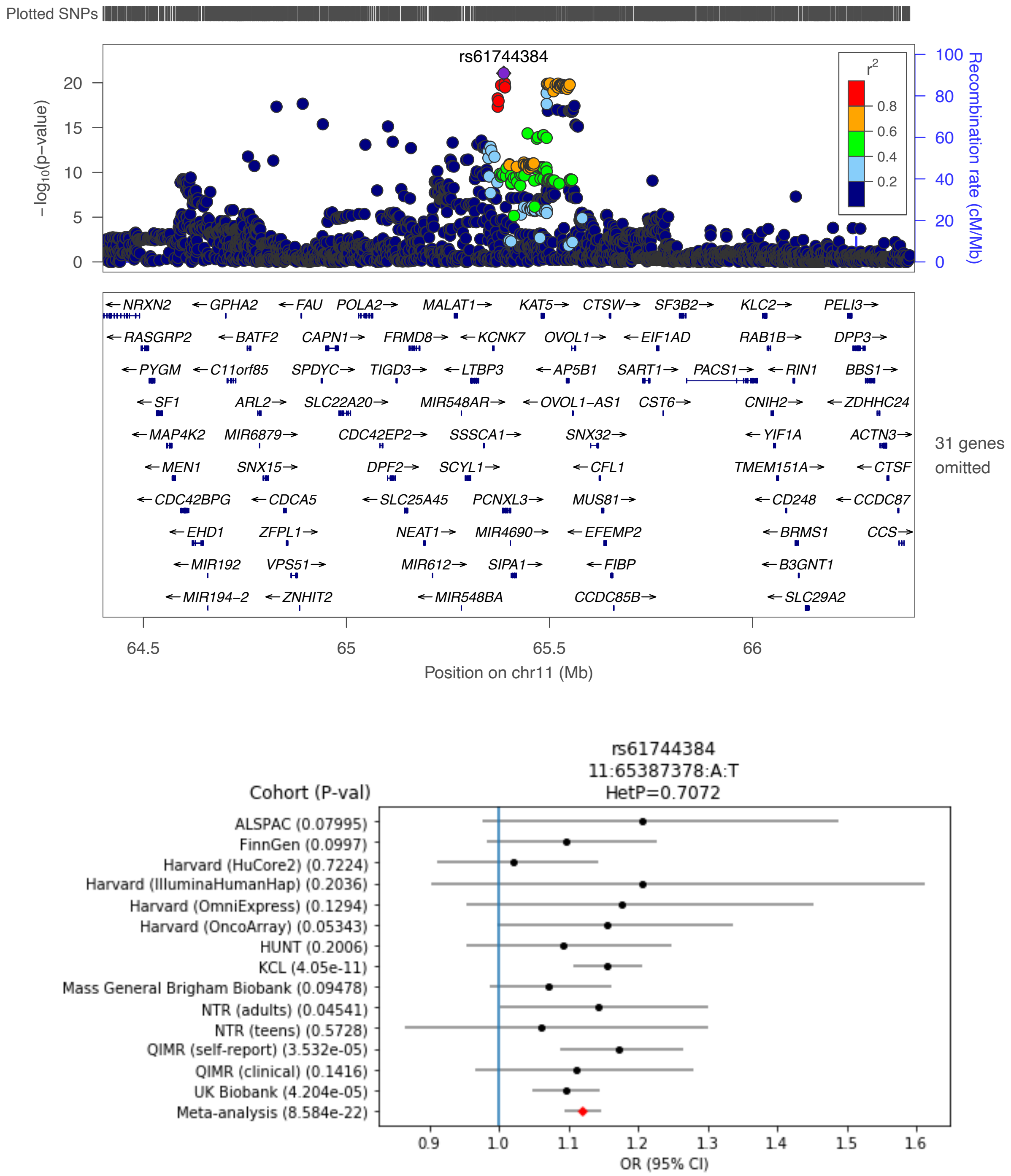

Supplementary Figure 4(xiv): Upper: meta-analysis locuszoom plot for established 11q13.1 locus. Lower: forest plot for established 11q13.1 locus, presented as odds ratio +/- 95% confidence intervals. Association *P*-values annotated for meta-analysis (two-sided *Z*-test, not adjusted for multiple testing) and each cohort (calculated as detailed in Supplementary Note).

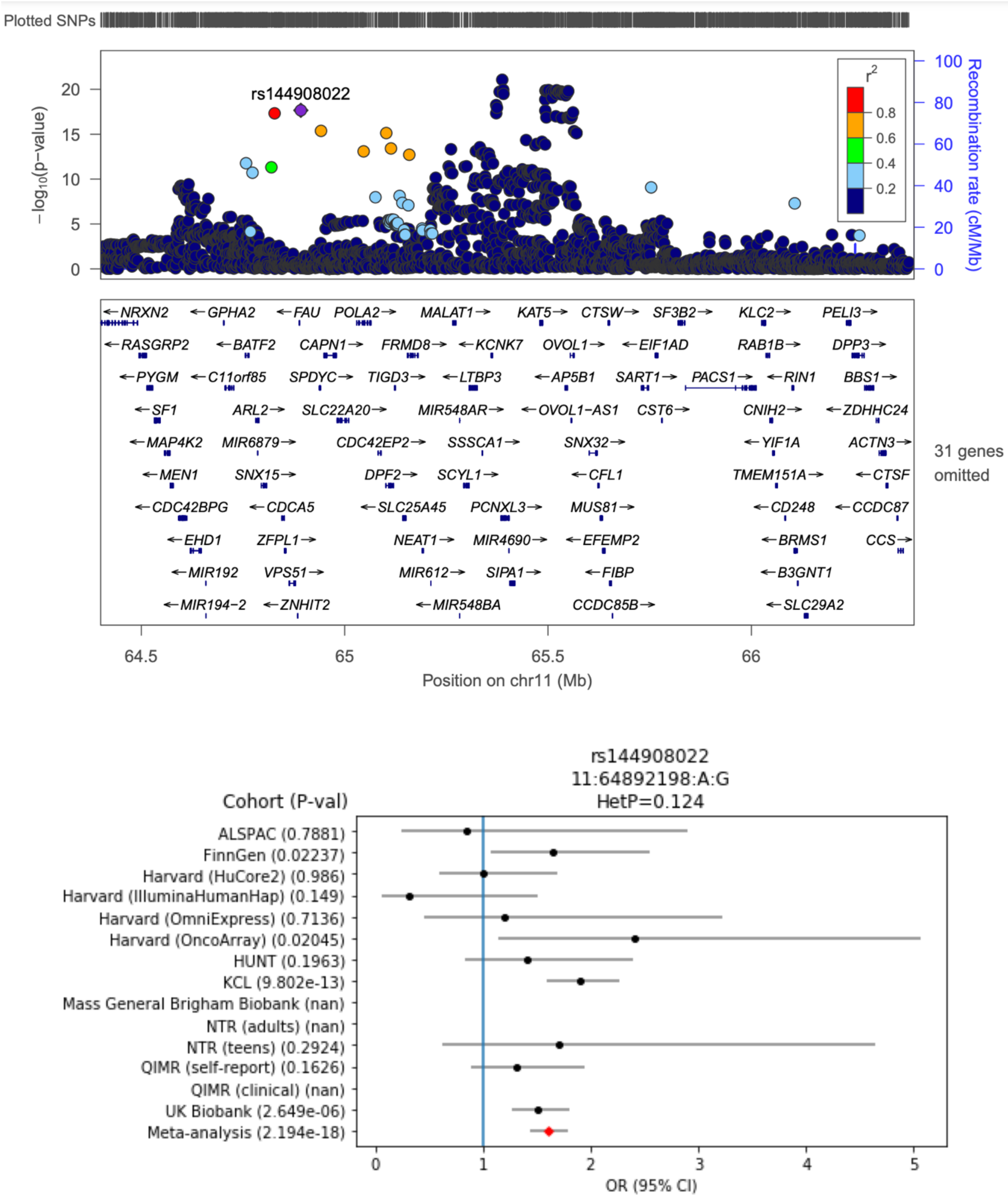

Supplementary Figure 4(xv): Upper: meta-analysis locuszoom plot for second independent signal at established 11q13.1 locus. Lower: forest plot for second independent signal at established 11q13.1 locus, presented as odds ratio +/- 95% confidence intervals. Association *P*-values annotated for meta-analysis (two-sided *Z*-test, not adjusted for multiple testing) and each cohort (calculated as detailed in Supplementary Note).

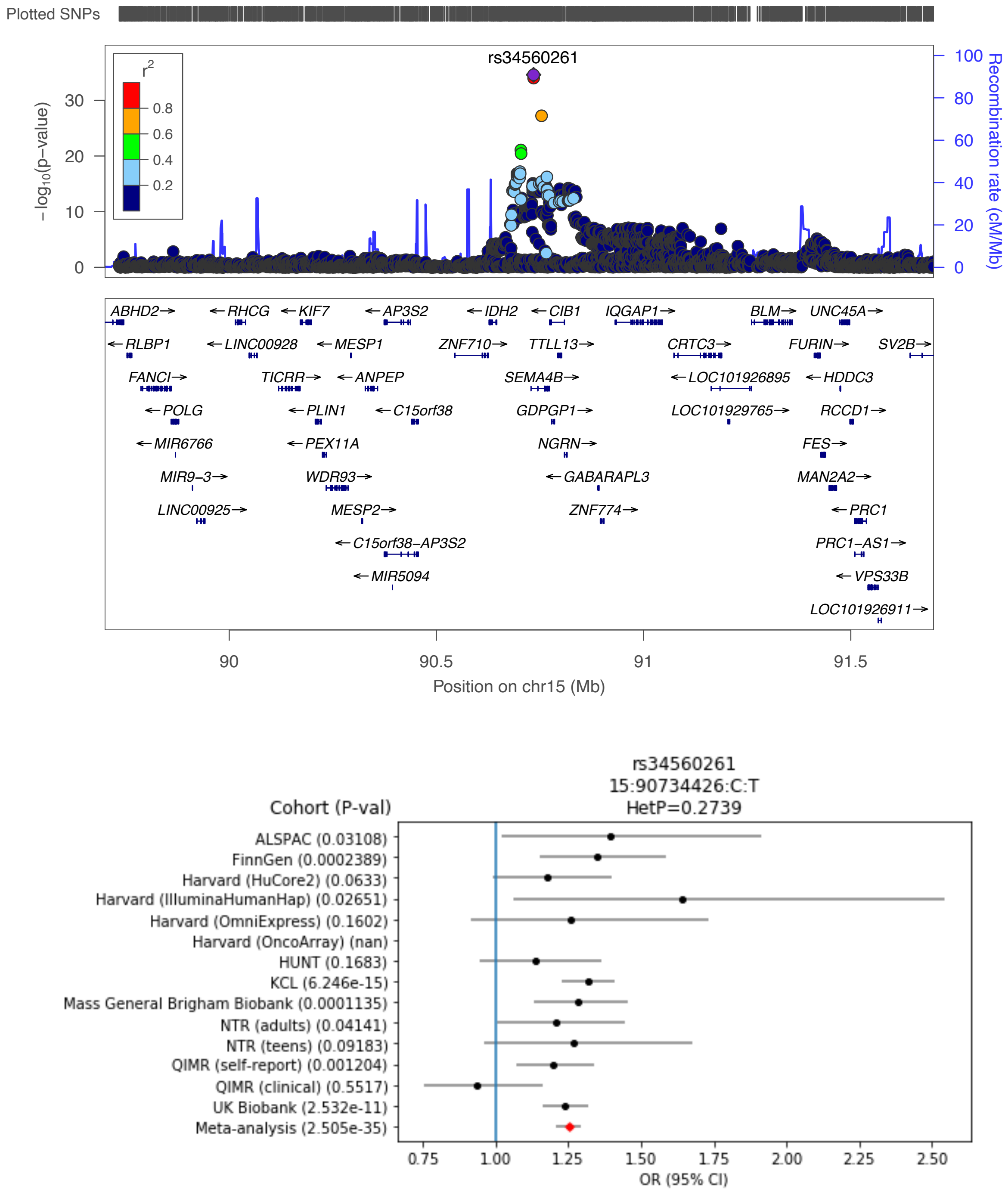

Supplementary Figure 4(xvi): Upper: meta-analysis locuszoom plot for established 15q26.1 locus. Lower: forest plot for established 15q26.1 locus, presented as odds ratio +/- 95% confidence intervals. Association *P*-values annotated for meta-analysis (two-sided *Z*-test, not adjusted for multiple testing) and each cohort (calculated as detailed in Supplementary Note).

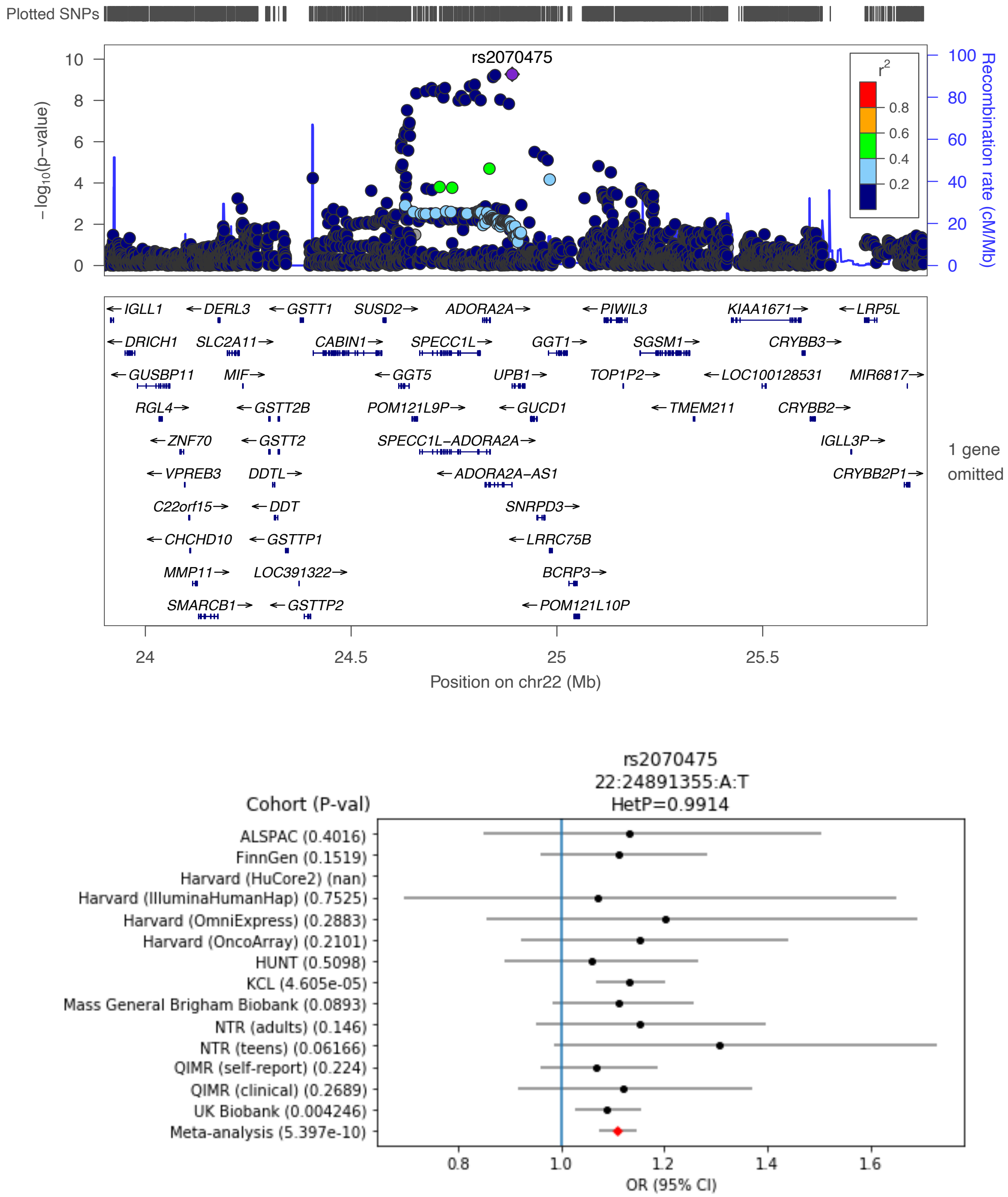

Supplementary Figure 4(xvii): Upper: meta-analysis locuszoom plot for established 22q11.23 locus. Lower: forest plot for established 22q11.23 locus, presented as odds ratio +/- 95% confidence intervals. Association *P*-values annotated for meta-analysis (two-sided *Z*-test, not adjusted for multiple testing) and each cohort (calculated as detailed in Supplementary Note).

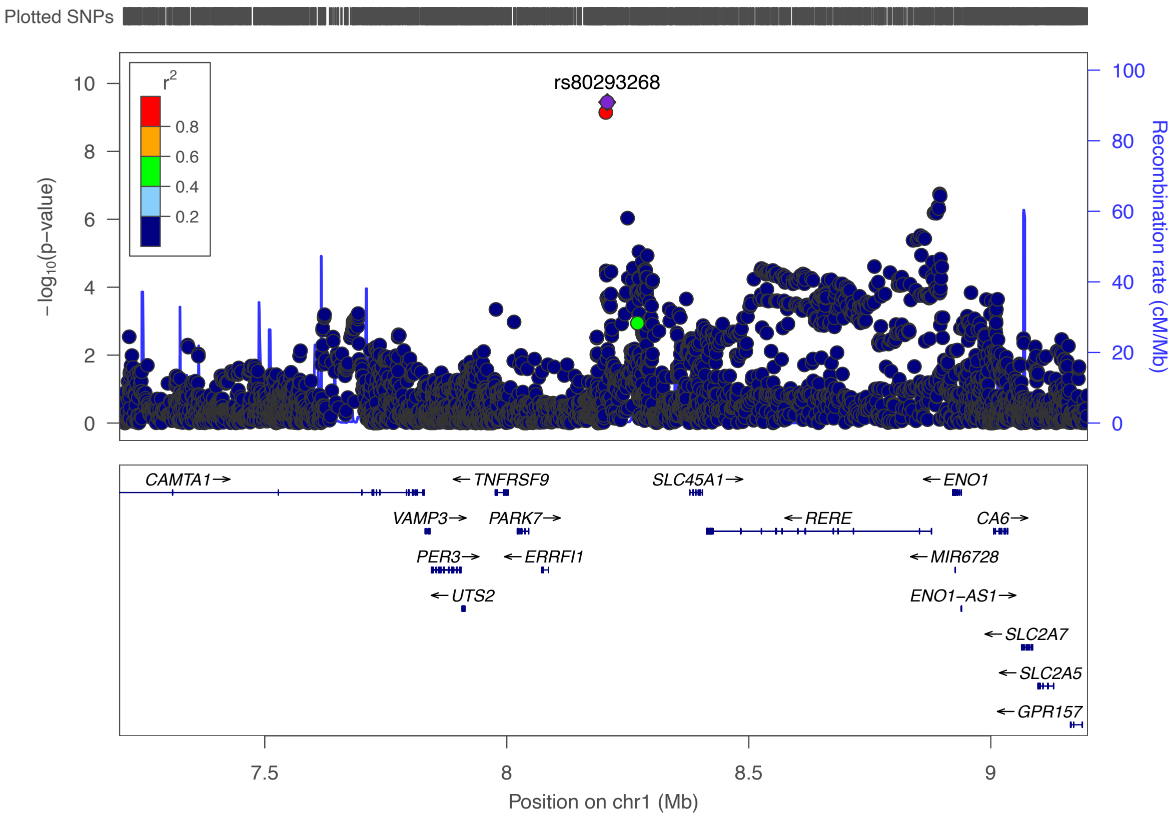

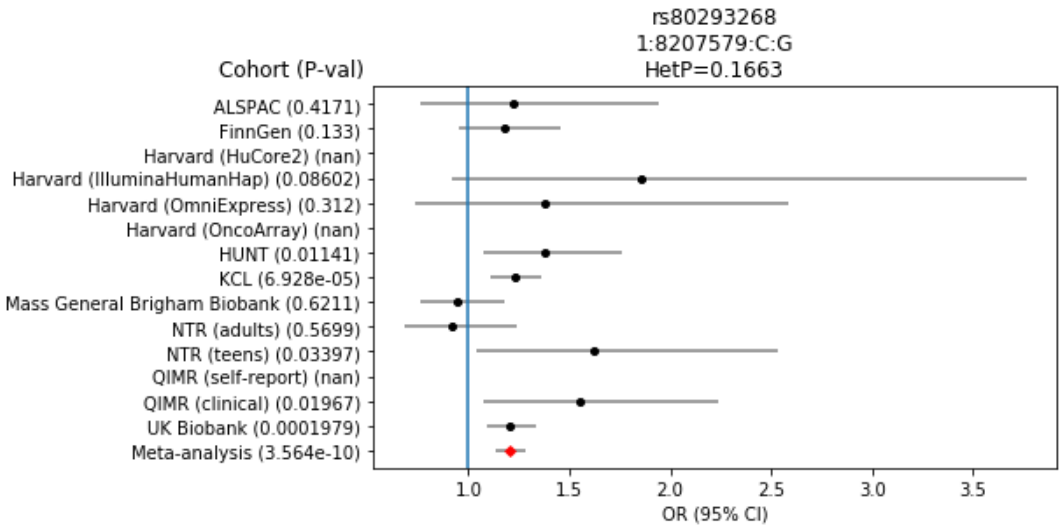

Supplementary Figure 5(i): Upper: meta-analysis locuszoom plot for novel 1p36.23 locus. Lower: forest plot for novel 1p36.23 locus, presented as odds ratio +/- 95% confidence intervals. Association *P*-values annotated for meta-analysis (two-sided *Z*-test, not adjusted for multiple testing) and each cohort (calculated as detailed in Supplementary Note).

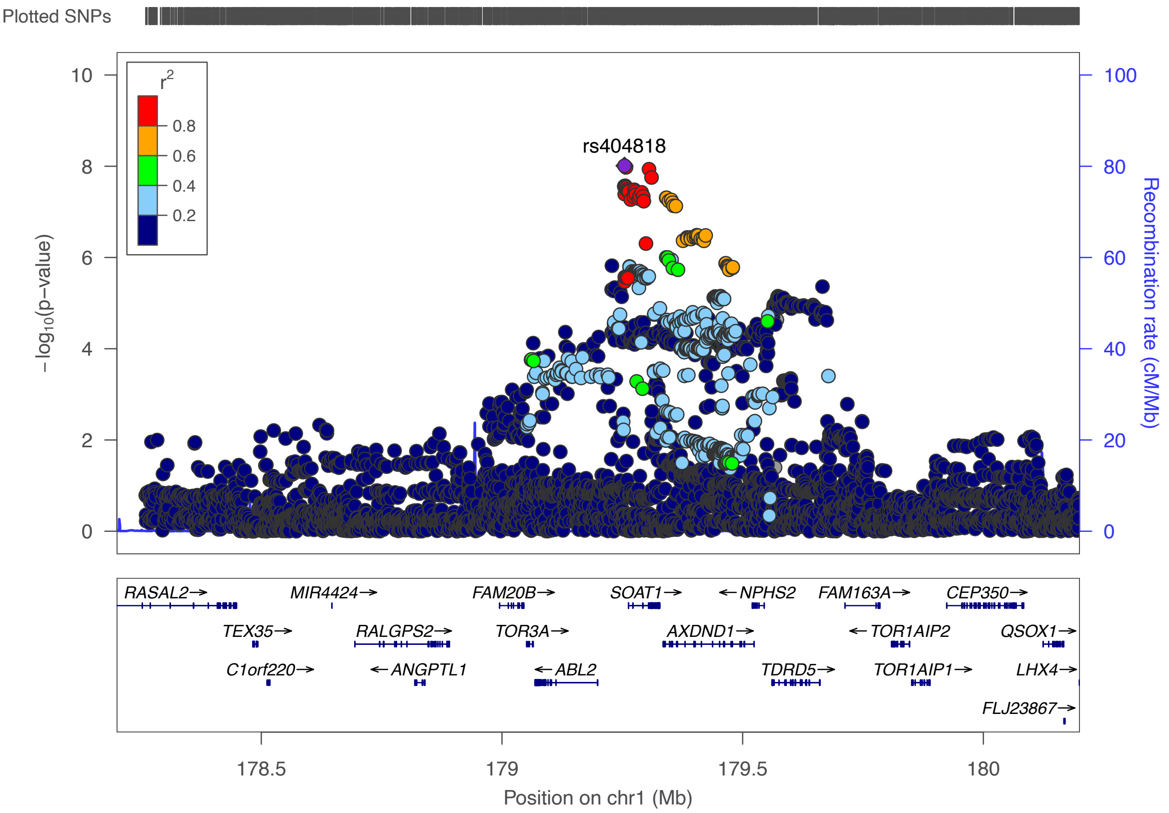

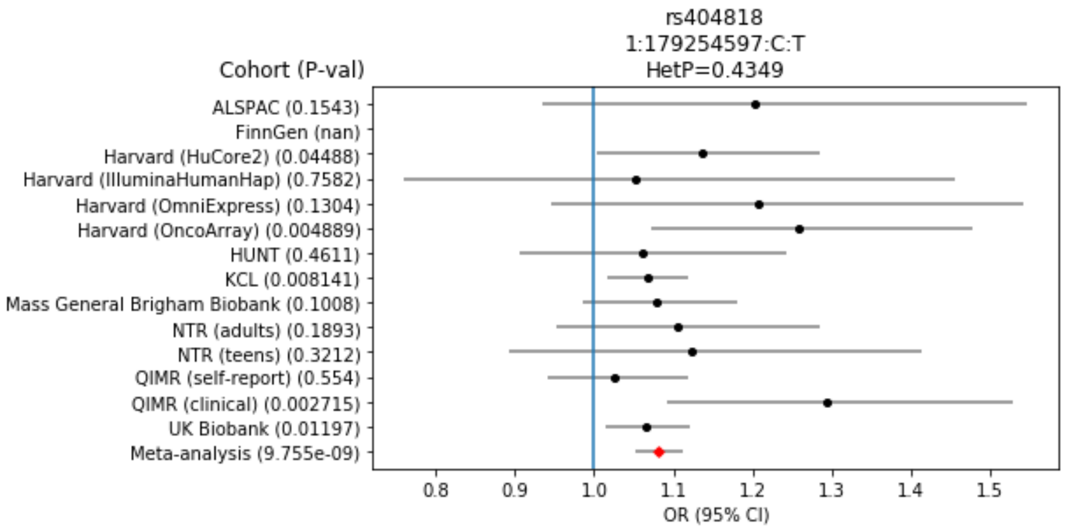

Supplementary Figure 5(ii): Upper: meta-analysis locuszoom plot for novel 1q25.2 locus. Lower: forest plot for novel 1q25.2 locus, presented as odds ratio +/- 95% confidence intervals. Association *P*-values annotated for meta-analysis (two-sided *Z*-test, not adjusted for multiple testing) and each cohort (calculated as detailed in Supplementary Note).

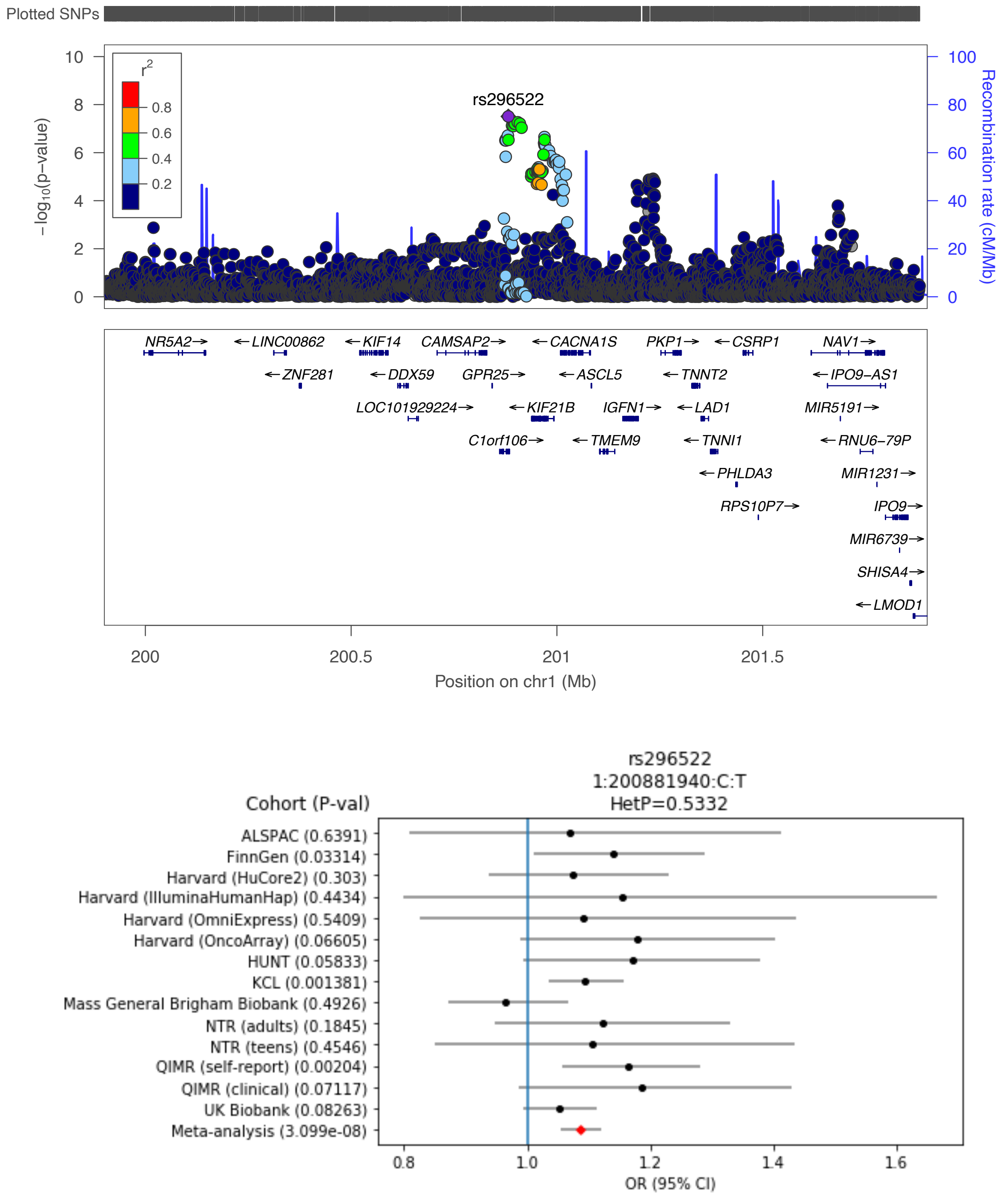

Supplementary Figure 5(iii): Upper: meta-analysis locuszoom plot for novel 1q32.1 locus. Lower: forest plot for novel 1q32.1 locus, presented as odds ratio +/- 95% confidence intervals. Association *P*-values annotated for meta-analysis (two-sided *Z*-test, not adjusted for multiple testing) and each cohort (calculated as detailed in Supplementary Note).

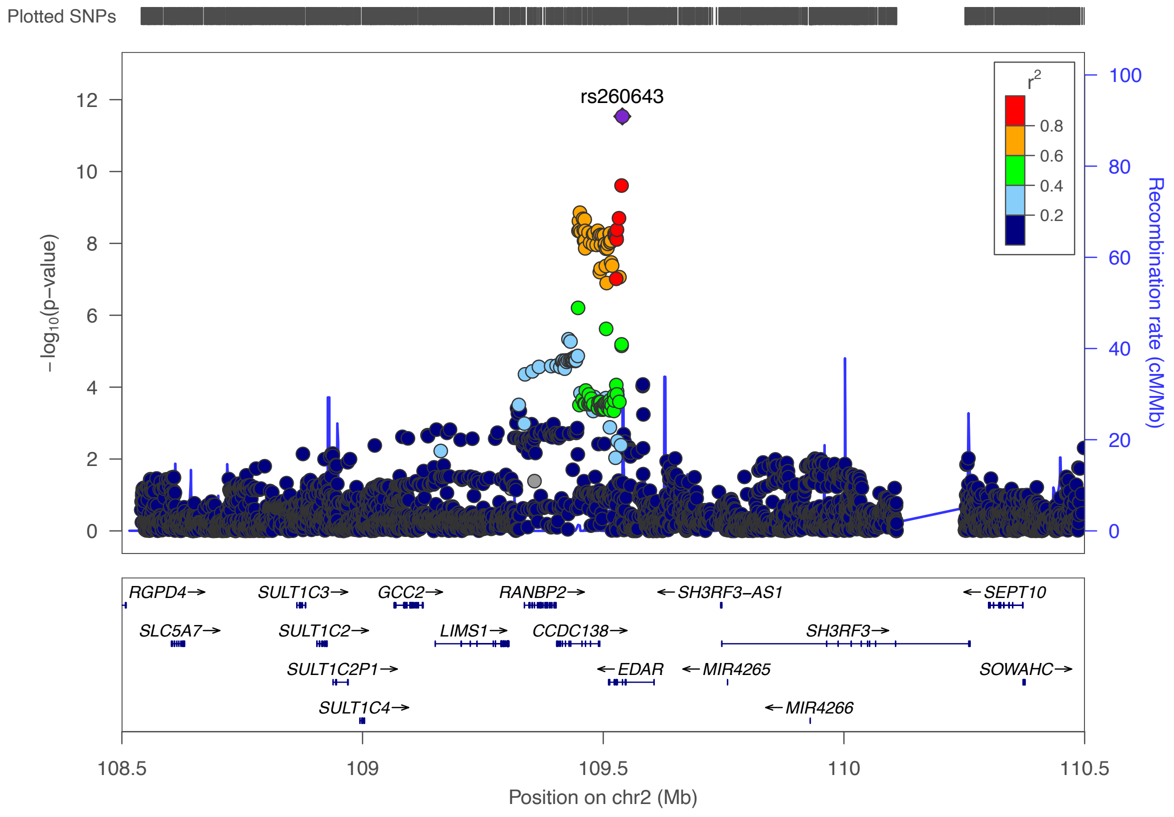

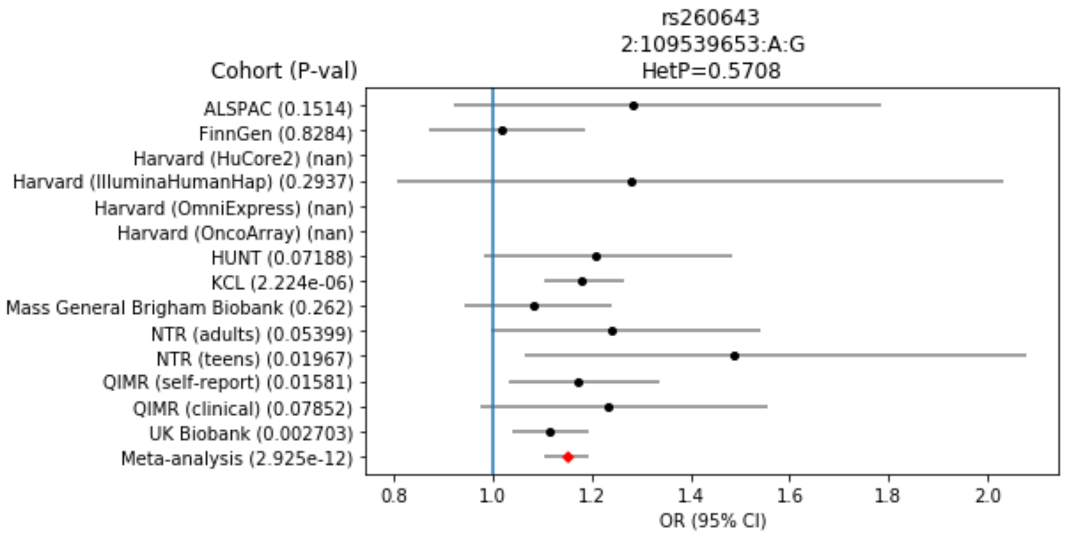

Supplementary Figure 5(iv): Upper: meta-analysis locuszoom plot for novel 2q12.3 locus. Lower: forest plot for novel 2q12.3 locus, presented as odds ratio +/- 95% confidence intervals. Association *P*-values annotated for meta-analysis (two-sided *Z*-test, not adjusted for multiple testing) and each cohort (calculated as detailed in Supplementary Note).

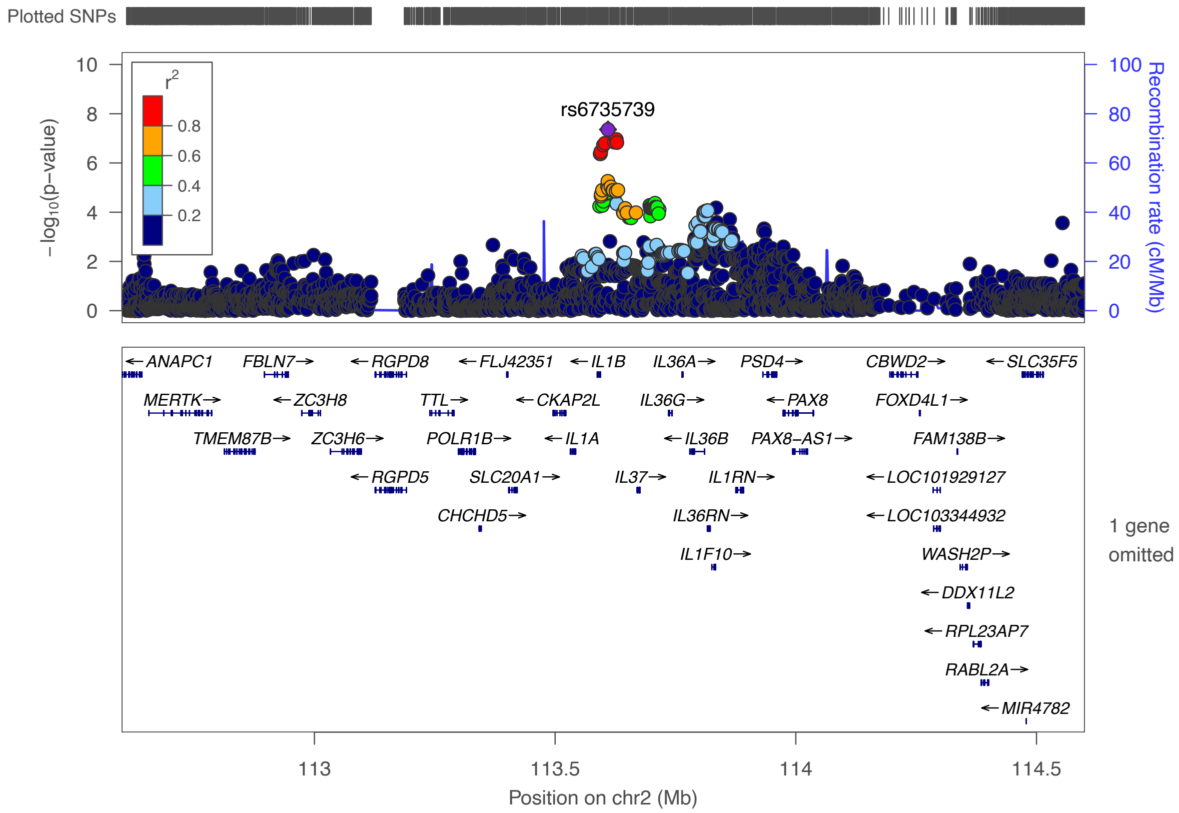

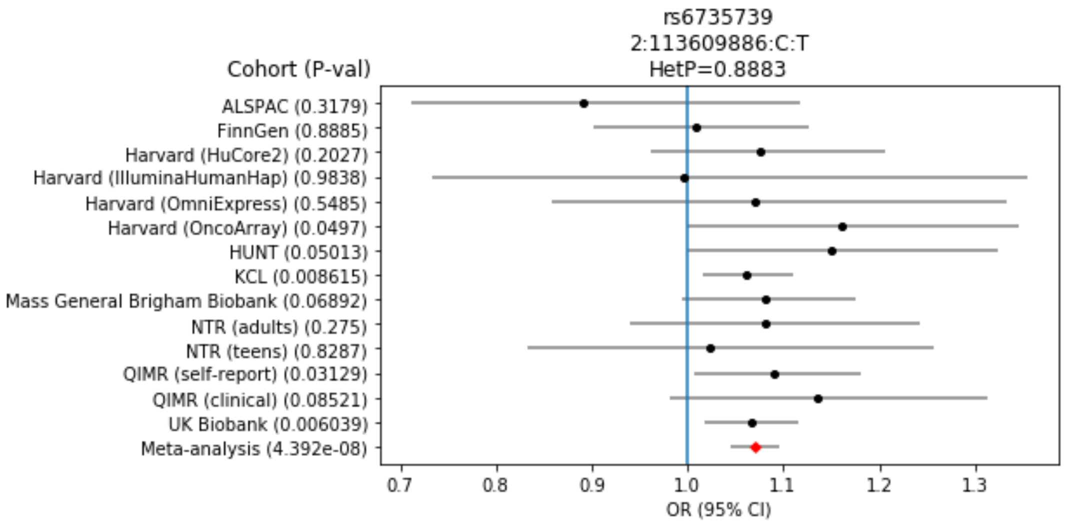

Supplementary Figure 5(v): Upper: meta-analysis locuszoom plot for novel 2q13 locus. Lower: forest plot for novel 2q13 locus, presented as odds ratio +/- 95% confidence intervals. Association *P*-values annotated for meta-analysis (two-sided *Z*-test, not adjusted for multiple testing) and each cohort (calculated as detailed in Supplementary Note).

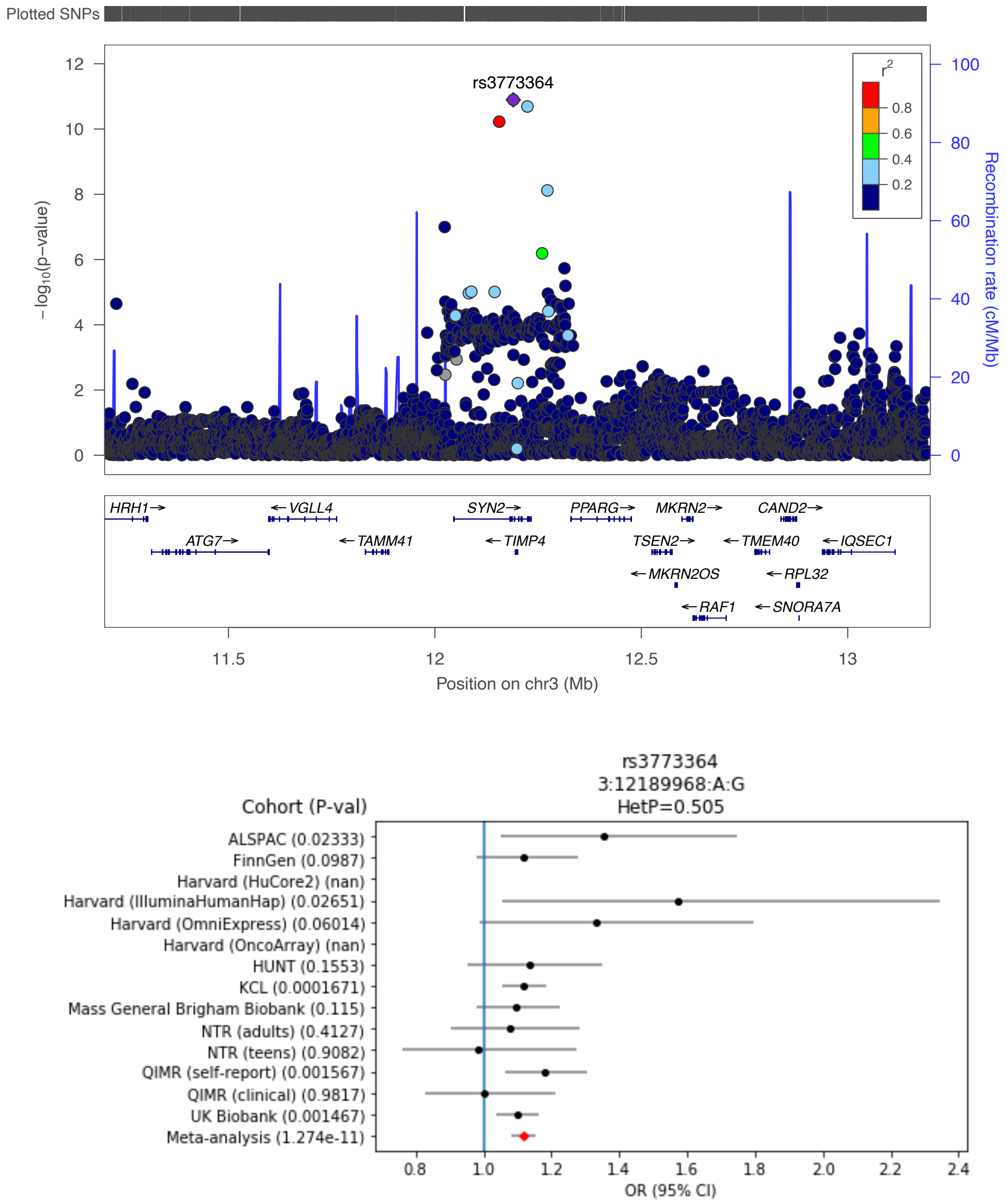

Supplementary Figure 5(vi): Upper: meta-analysis locuszoom plot for novel 3p25.2 locus. Lower: forest plot for novel 3p25.2 locus, presented as odds ratio +/- 95% confidence intervals. Association *P*-values annotated for meta-analysis (two-sided *Z*-test, not adjusted for multiple testing) and each cohort (calculated as detailed in Supplementary Note).

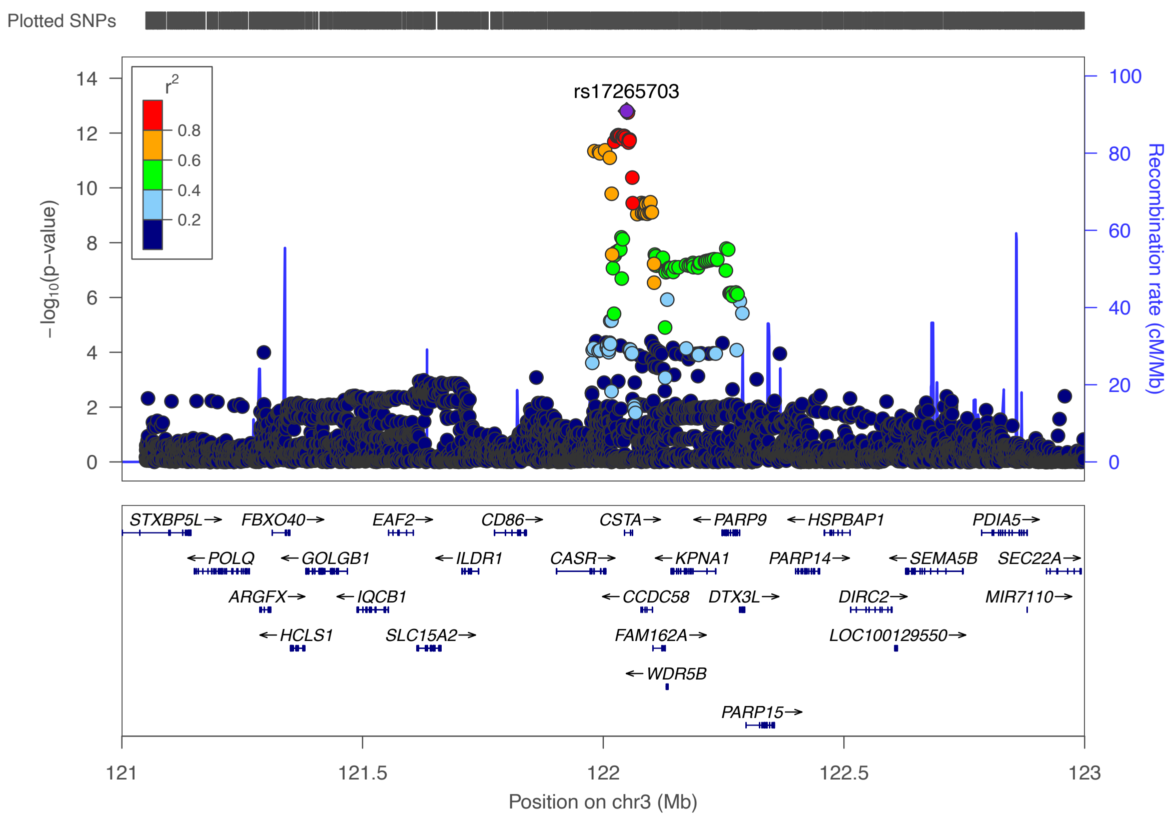

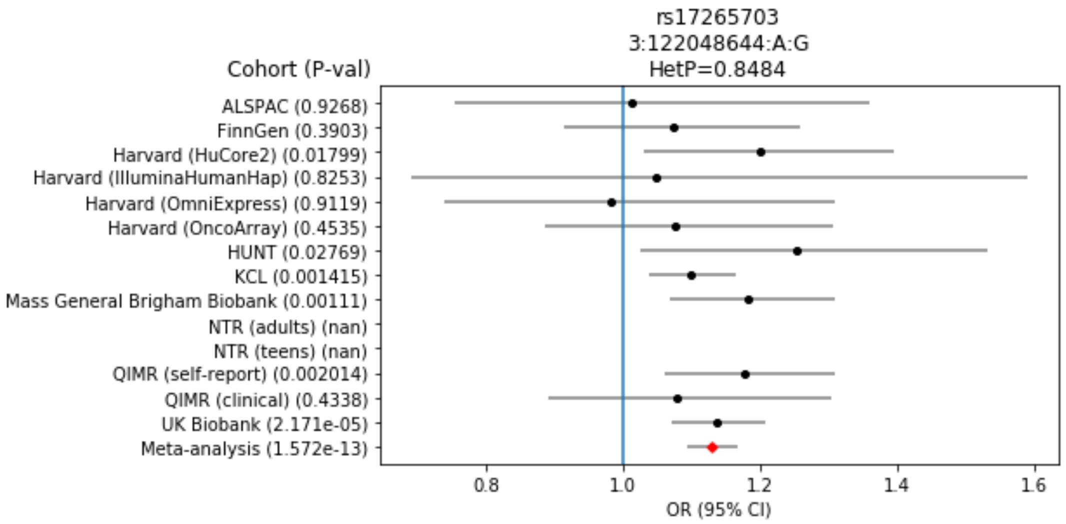

Supplementary Figure 5(vii): Upper: meta-analysis locuszoom plot for novel 3q21.1 locus. Lower: forest plot for novel 3q21.1 locus, presented as odds ratio +/- 95% confidence intervals. Association *P*-values annotated for meta-analysis (two-sided *Z*-test, not adjusted for multiple testing) and each cohort (calculated as detailed in Supplementary Note).

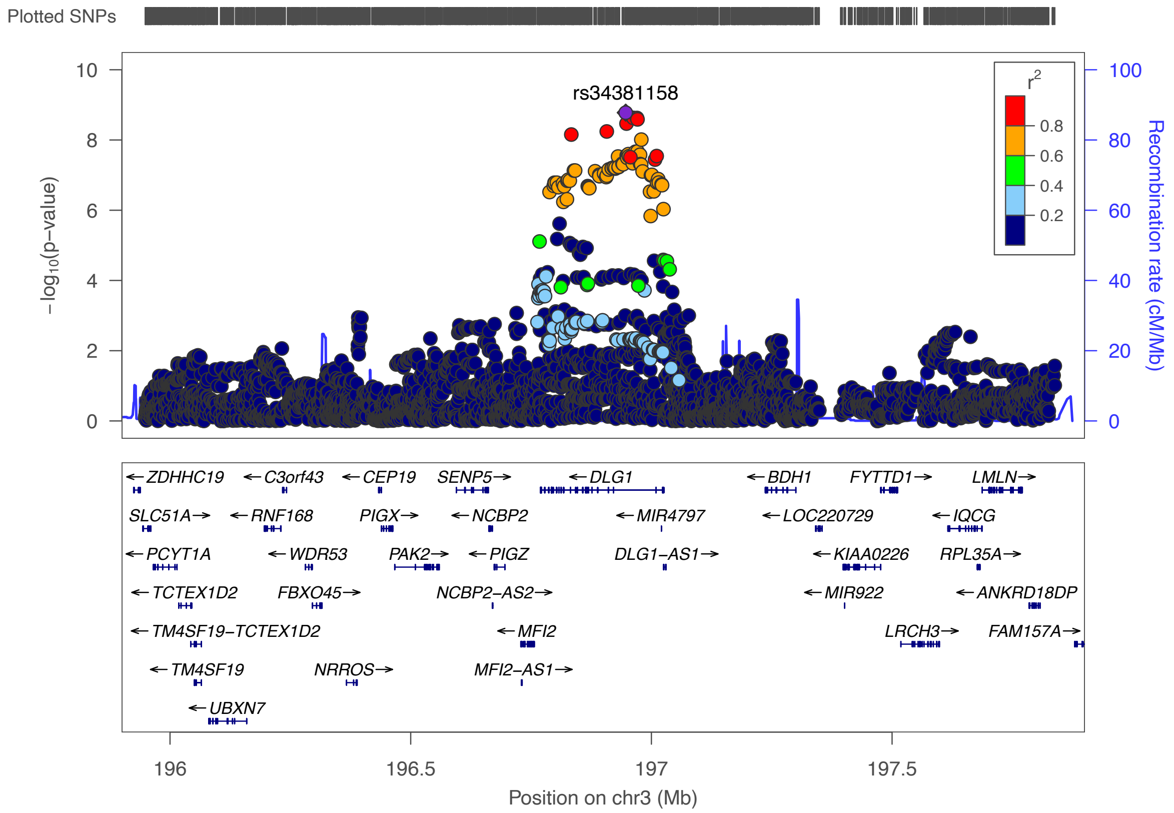

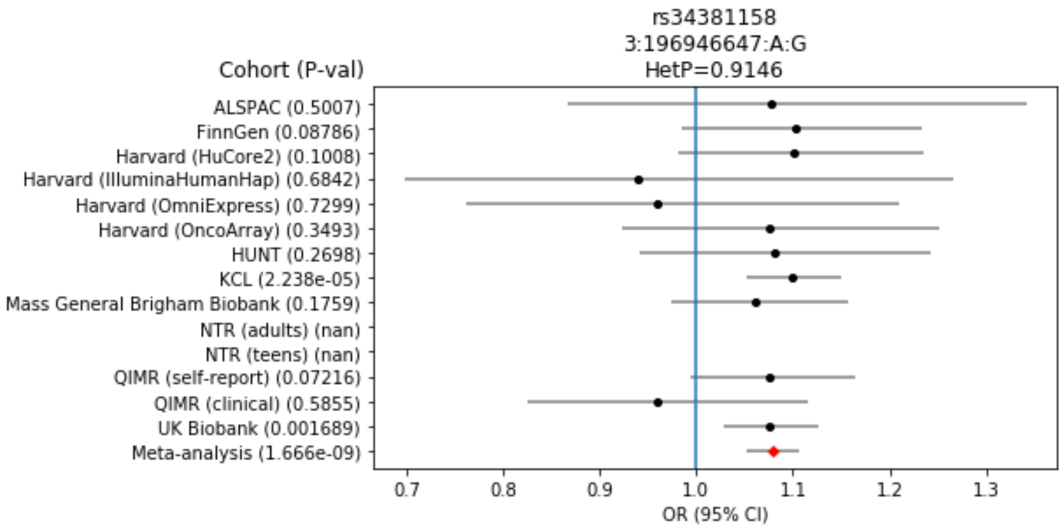

Supplementary Figure 5(viii): Upper: meta-analysis locuszoom plot for novel 3q29 locus. Lower: forest plot for novel 3q29 locus, presented as odds ratio +/- 95% confidence intervals. Association *P*-values annotated for meta-analysis (two-sided *Z*-test, not adjusted for multiple testing) and each cohort (calculated as detailed in Supplementary Note).

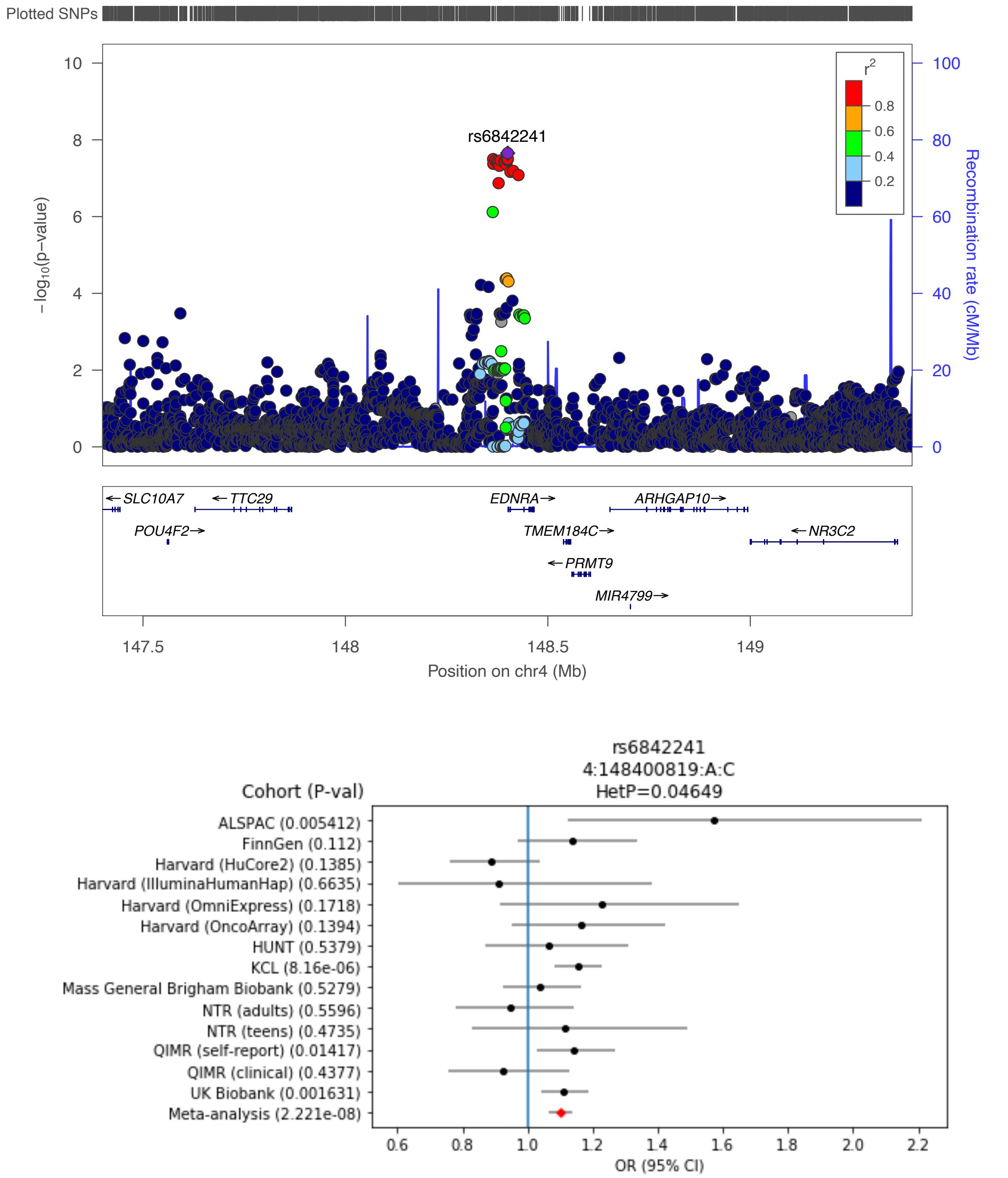

Supplementary Figure 5(ix): Upper: meta-analysis locuszoom plot for novel 4q31.22 locus. Lower: forest plot for novel 4q31.22 locus, presented as odds ratio +/- 95% confidence intervals. Association *P*-values annotated for meta-analysis (two-sided *Z*-test, not adjusted for multiple testing) and each cohort (calculated as detailed in Supplementary Note).

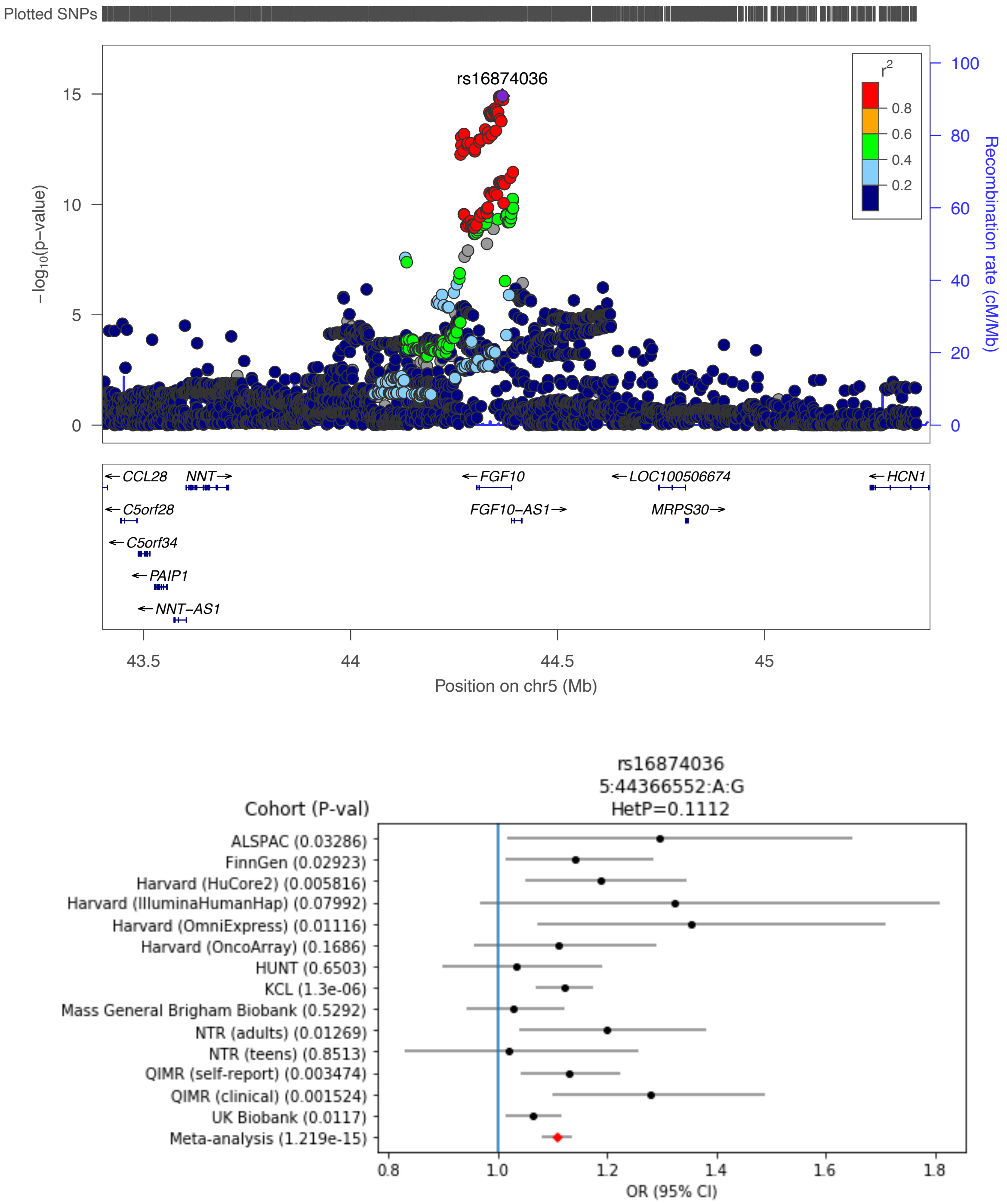

Supplementary Figure 5(x): Upper: meta-analysis locuszoom plot for novel 5p12 locus. Lower: forest plot for novel 5p12 locus, presented as odds ratio +/- 95% confidence intervals. Association *P*-values annotated for meta-analysis (two-sided *Z*-test, not adjusted for multiple testing) and each cohort (calculated as detailed in Supplementary Note).

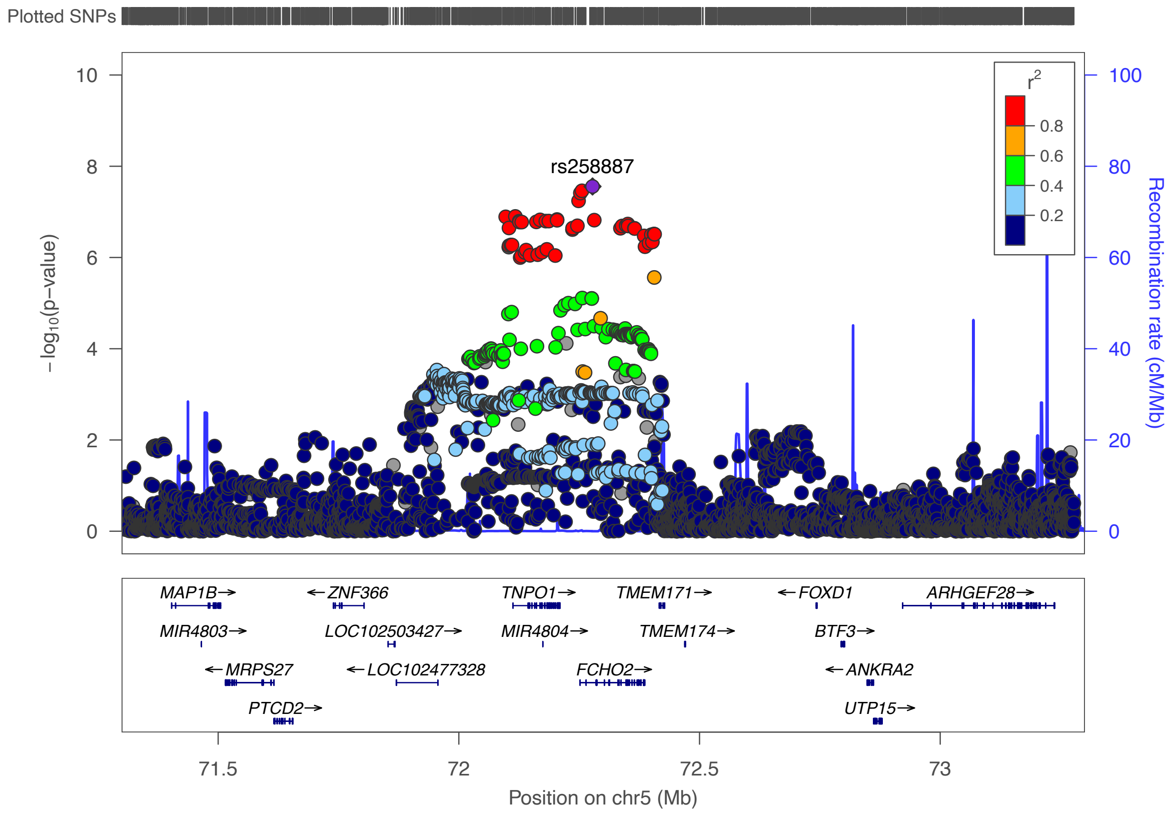

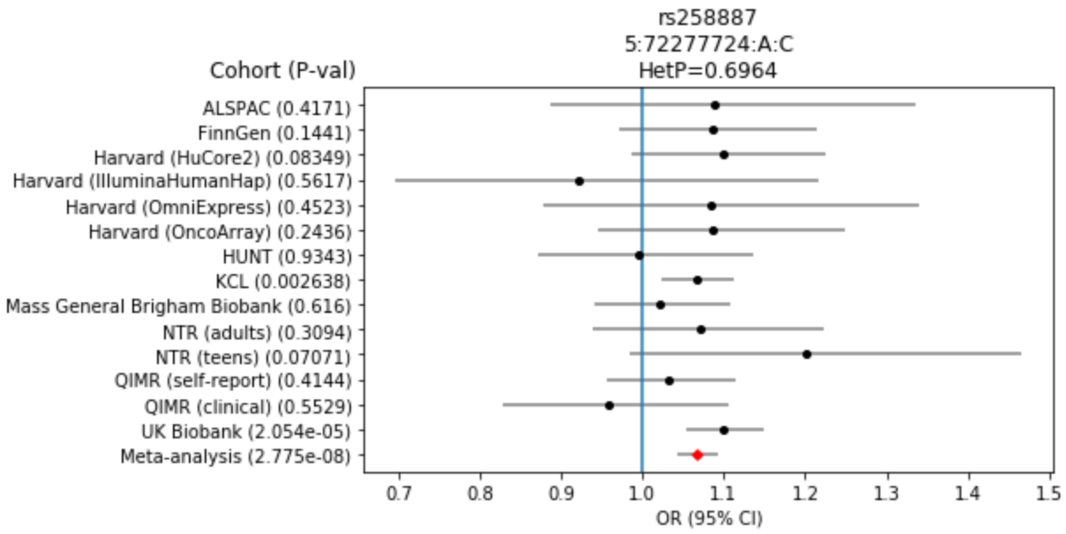

Supplementary Figure 5(xi): Upper: meta-analysis locuszoom plot for novel 5q13.2 locus. Lower: forest plot for novel 5q13.2 locus, presented as odds ratio +/- 95% confidence intervals. Association *P*-values annotated for meta-analysis (two-sided *Z*-test, not adjusted for multiple testing) and each cohort (calculated as detailed in Supplementary Note).

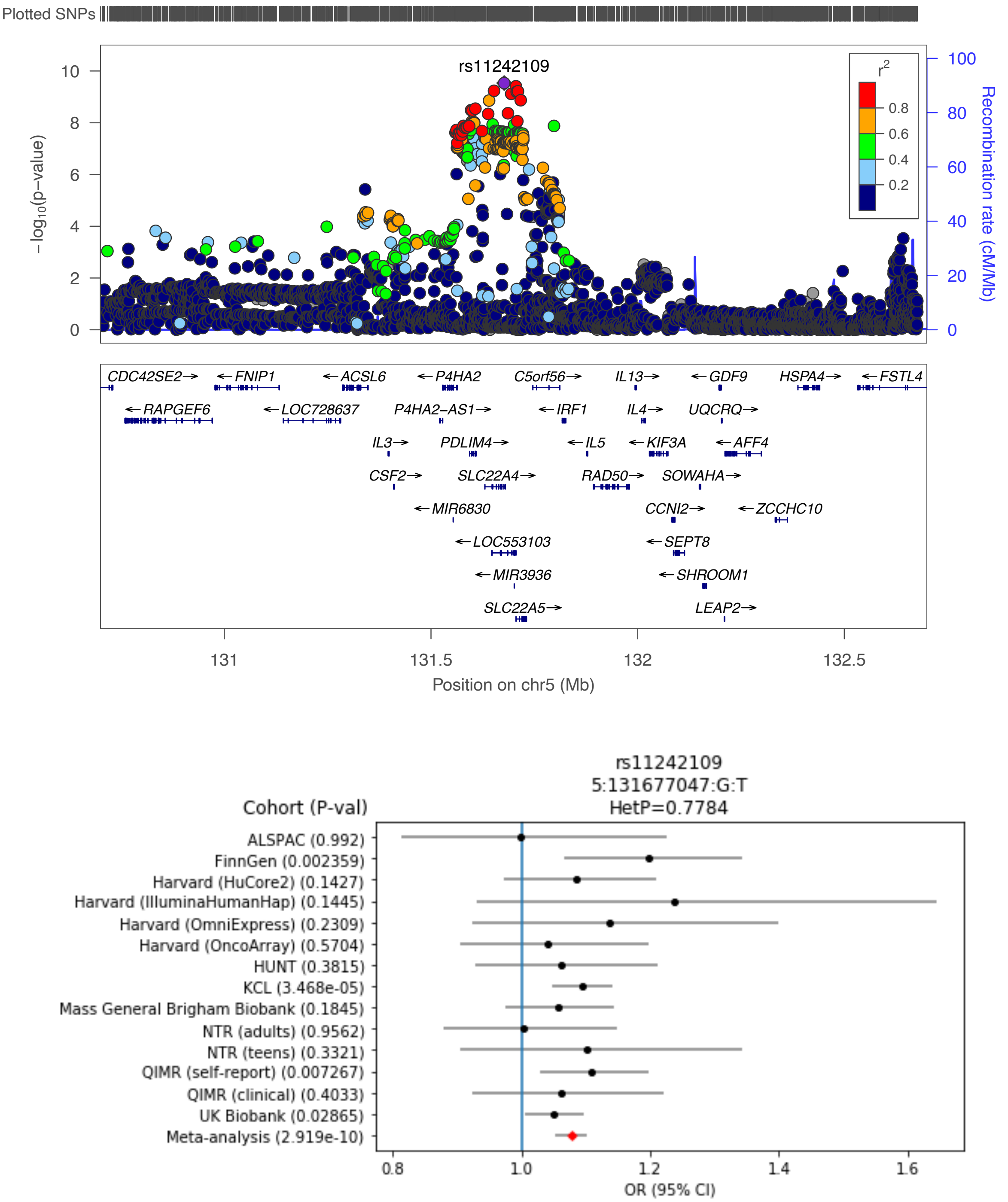

Supplementary Figure 5(xii): Upper: meta-analysis locuszoom plot for novel 5q31.1 locus. Lower: forest plot for novel 5q31.1 locus, presented as odds ratio +/- 95% confidence intervals. Association *P*-values annotated for meta-analysis (two-sided *Z*-test, not adjusted for multiple testing) and each cohort (calculated as detailed in Supplementary Note).

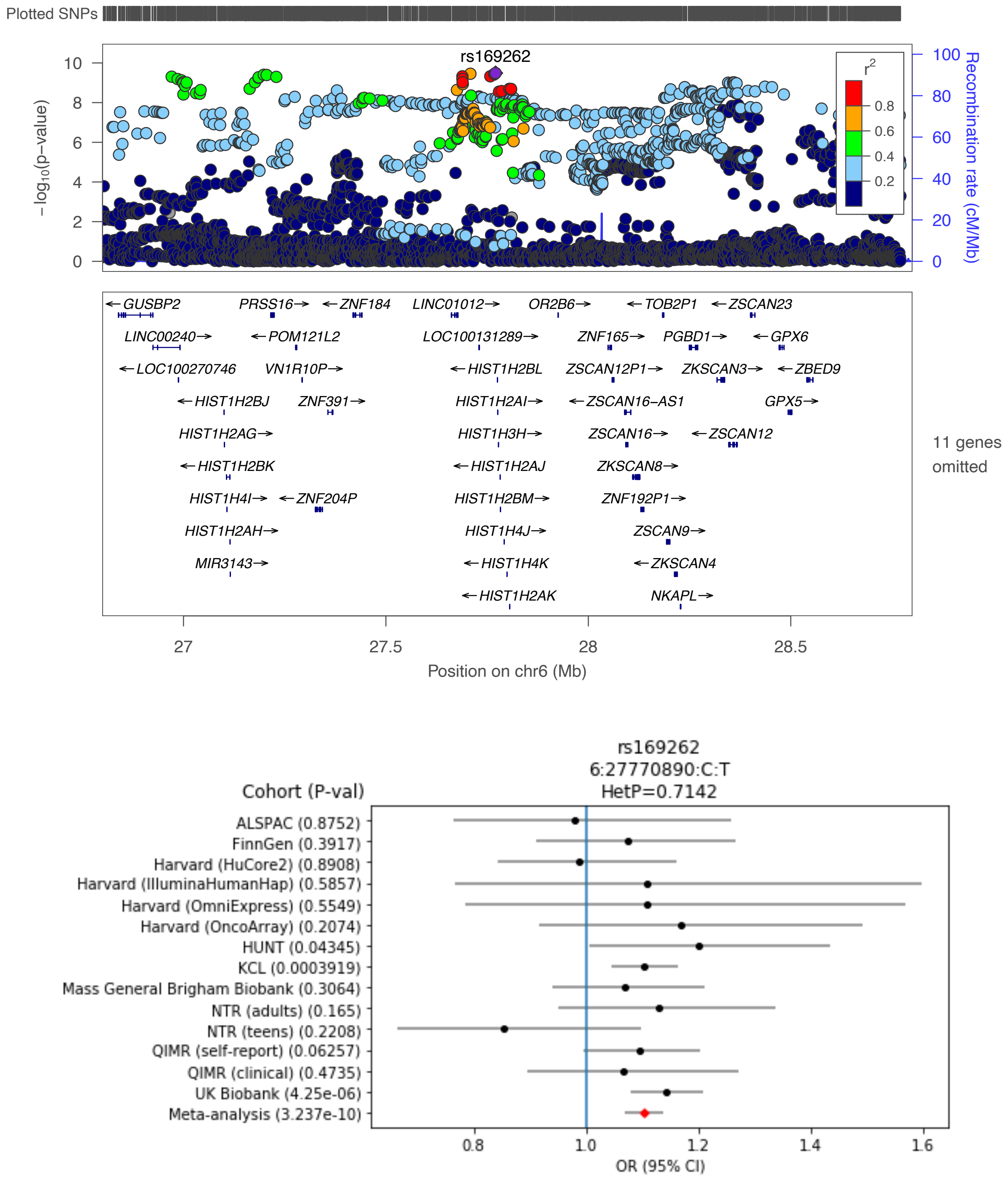

Supplementary Figure 5(xiii): Upper: meta-analysis locuszoom plot for novel 6p22.1 locus. Lower: forest plot for novel 6p22.1 locus, presented as odds ratio +/- 95% confidence intervals. Association *P*-values annotated for meta-analysis (two-sided *Z*-test, not adjusted for multiple testing) and each cohort (calculated as detailed in Supplementary Note).

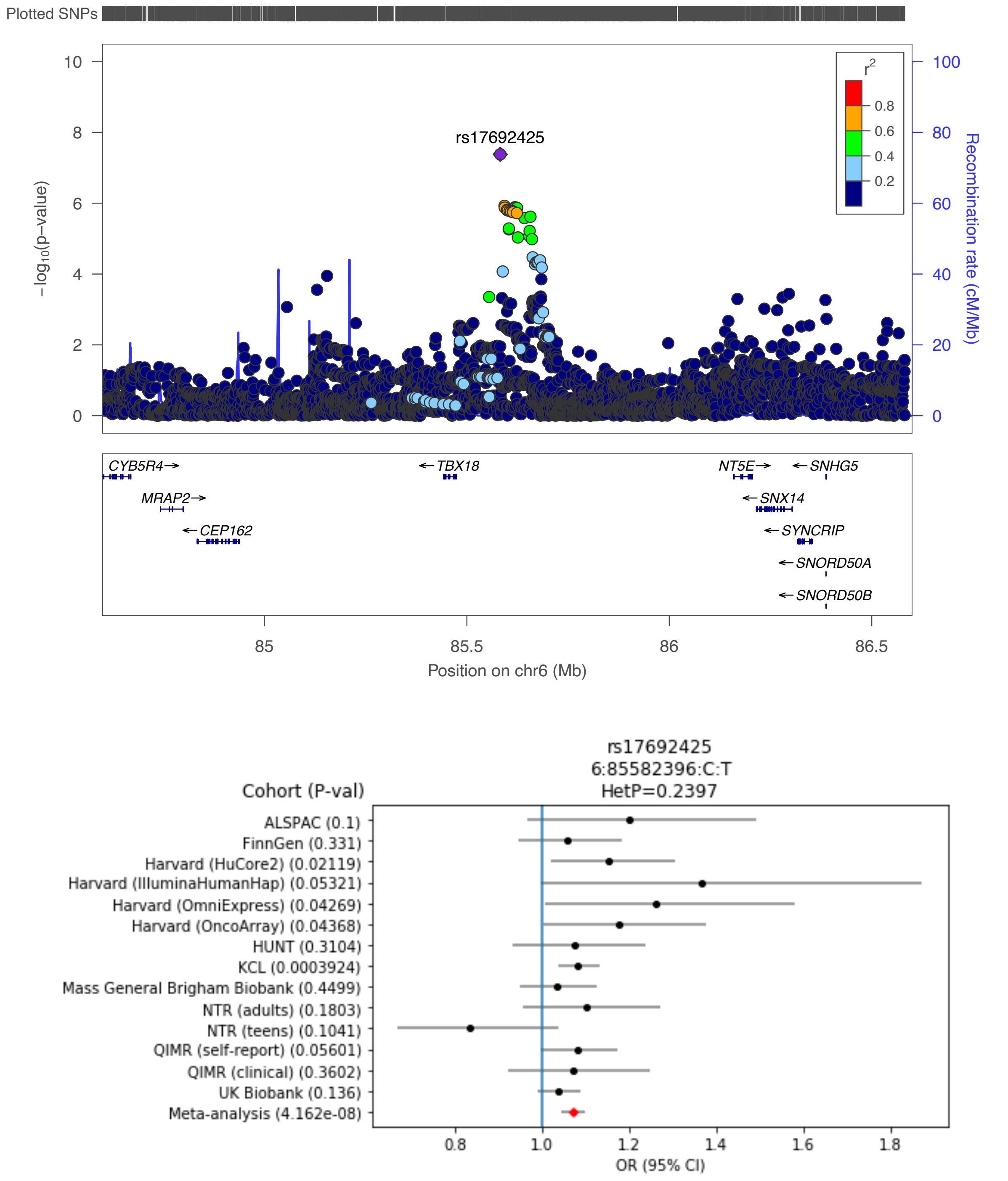

Supplementary Figure 5(xiv): Upper: meta-analysis locuszoom plot for novel 6q14.3 locus. Lower: forest plot for novel 6q14.3 locus, presented as odds ratio +/- 95% confidence intervals. Association *P*-values annotated for meta-analysis (two-sided *Z*-test, not adjusted for multiple testing) and each cohort (calculated as detailed in Supplementary Note).

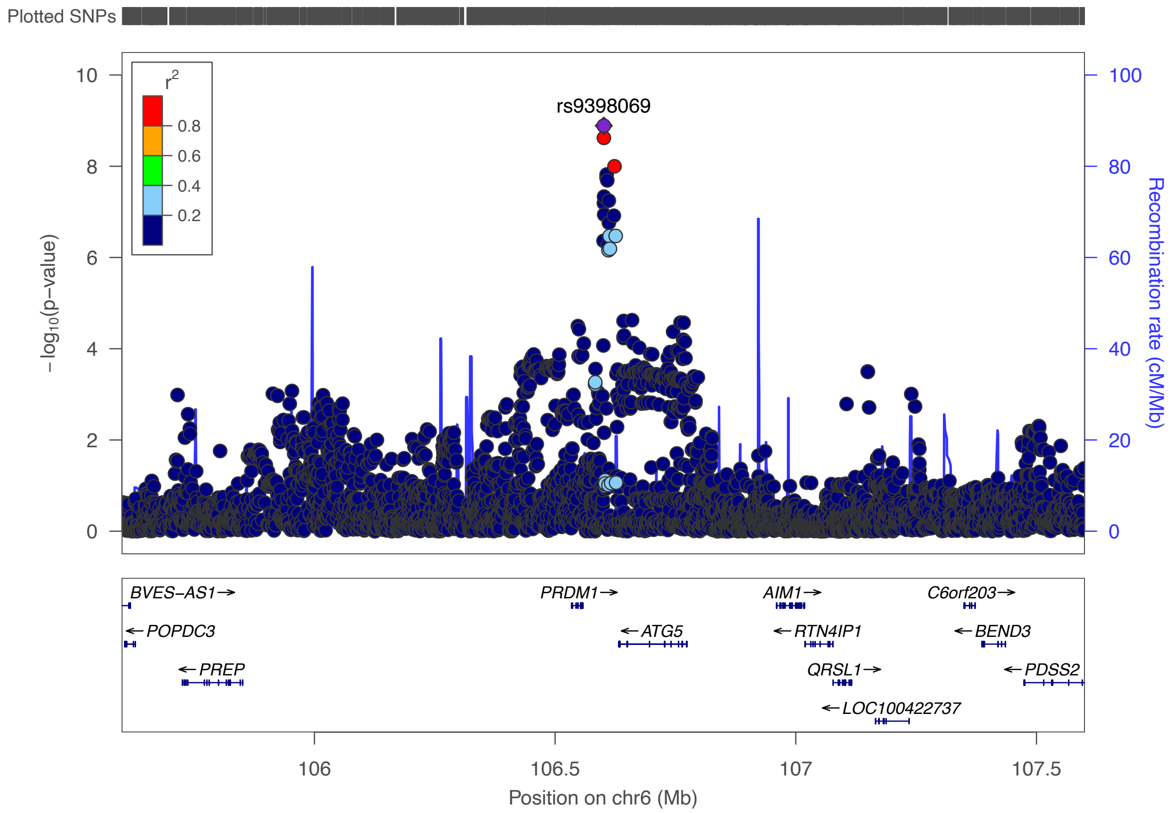

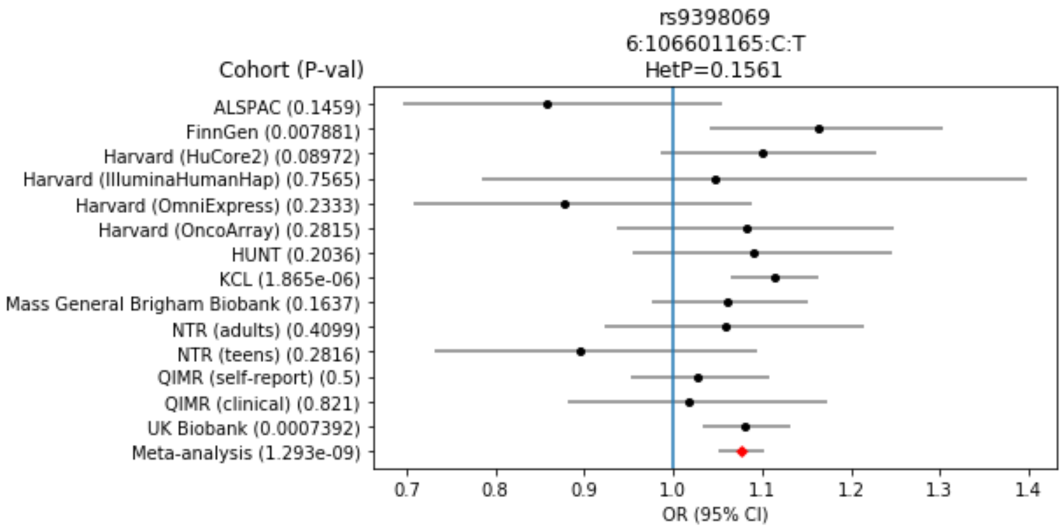

Supplementary Figure 5(xv): Upper: meta-analysis locuszoom plot for novel 6q21 locus. Lower: forest plot for novel 6q21 locus, presented as odds ratio +/- 95% confidence intervals. Association *P*-values annotated for meta-analysis (two-sided *Z*-test, not adjusted for multiple testing) and each cohort (calculated as detailed in Supplementary Note).

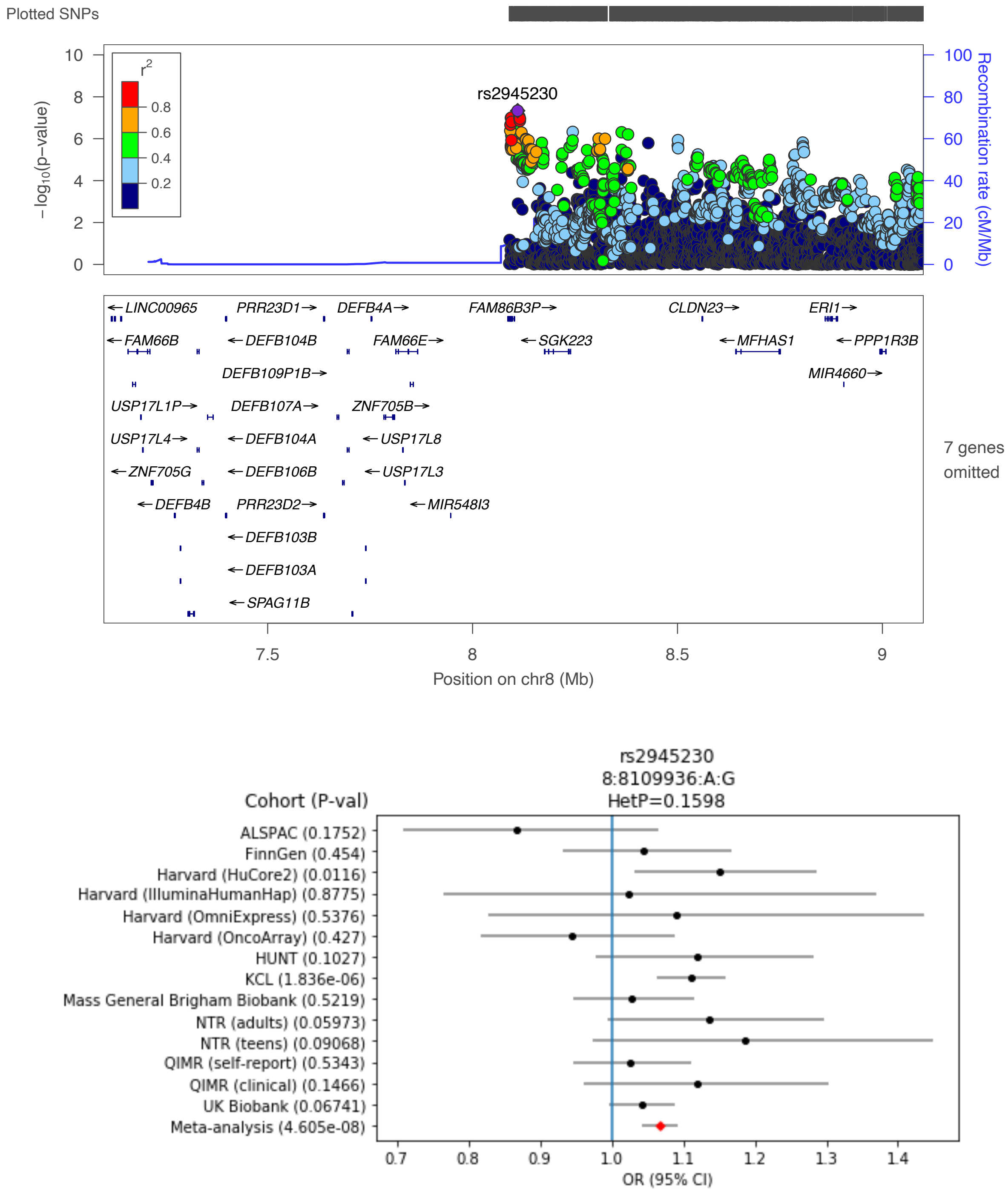

Supplementary Figure 5(xvi): Upper: meta-analysis locuszoom plot for novel 8p23.1 locus. Lower: forest plot for novel 8p23.1 locus, presented as odds ratio +/- 95% confidence intervals. Association *P*-values annotated for meta-analysis (two-sided *Z*-test, not adjusted for multiple testing) and each cohort (calculated as detailed in Supplementary Note).

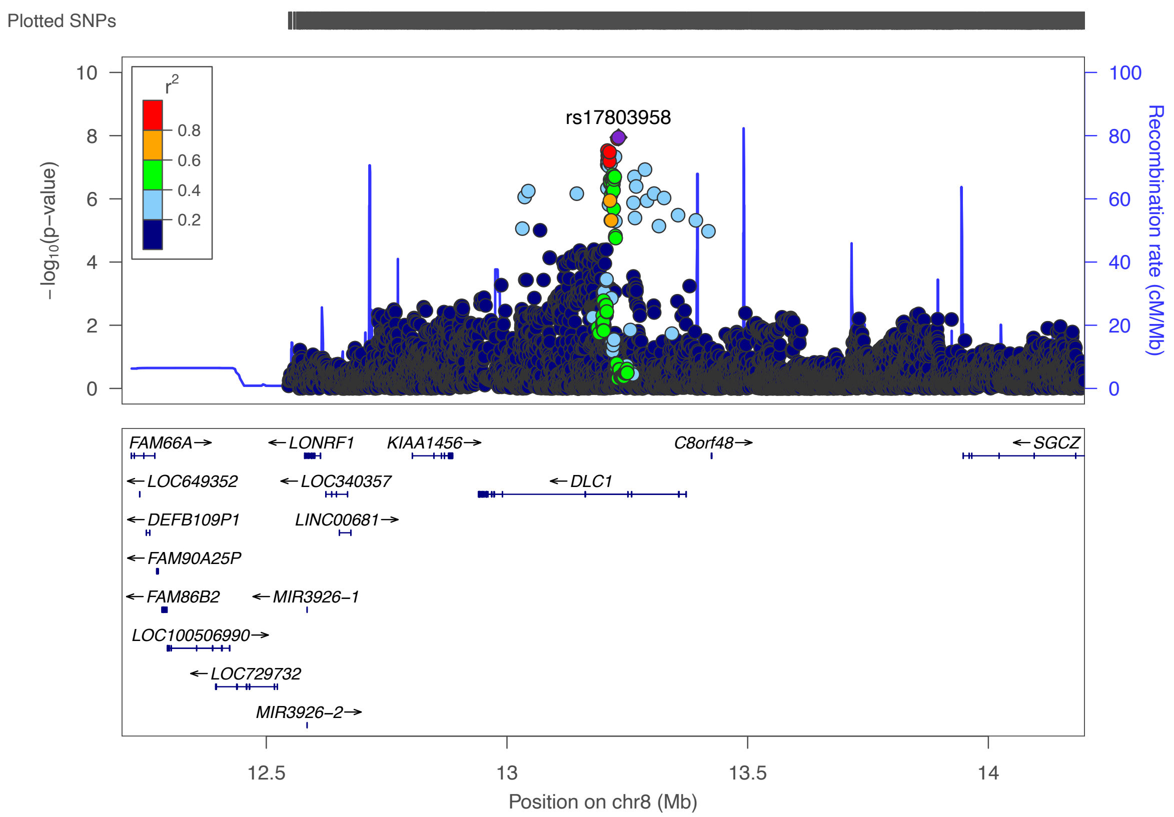

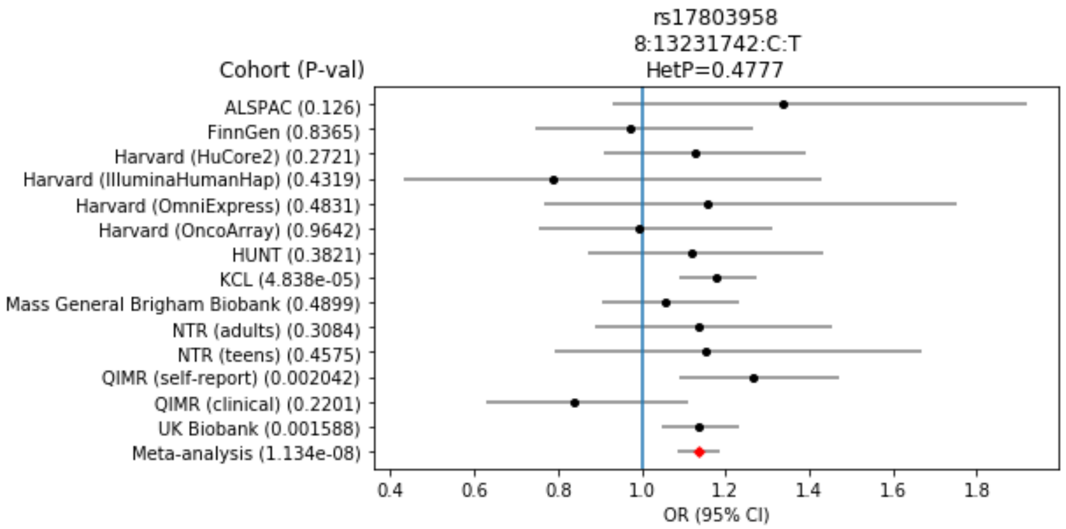

Supplementary Figure 5(xvii): Upper: meta-analysis locuszoom plot for novel 8p22 locus. Lower: forest plot for novel 8p22 locus, presented as odds ratio +/- 95% confidence intervals. Association *P*-values annotated for meta-analysis (two-sided *Z*-test, not adjusted for multiple testing) and each cohort (calculated as detailed in Supplementary Note).

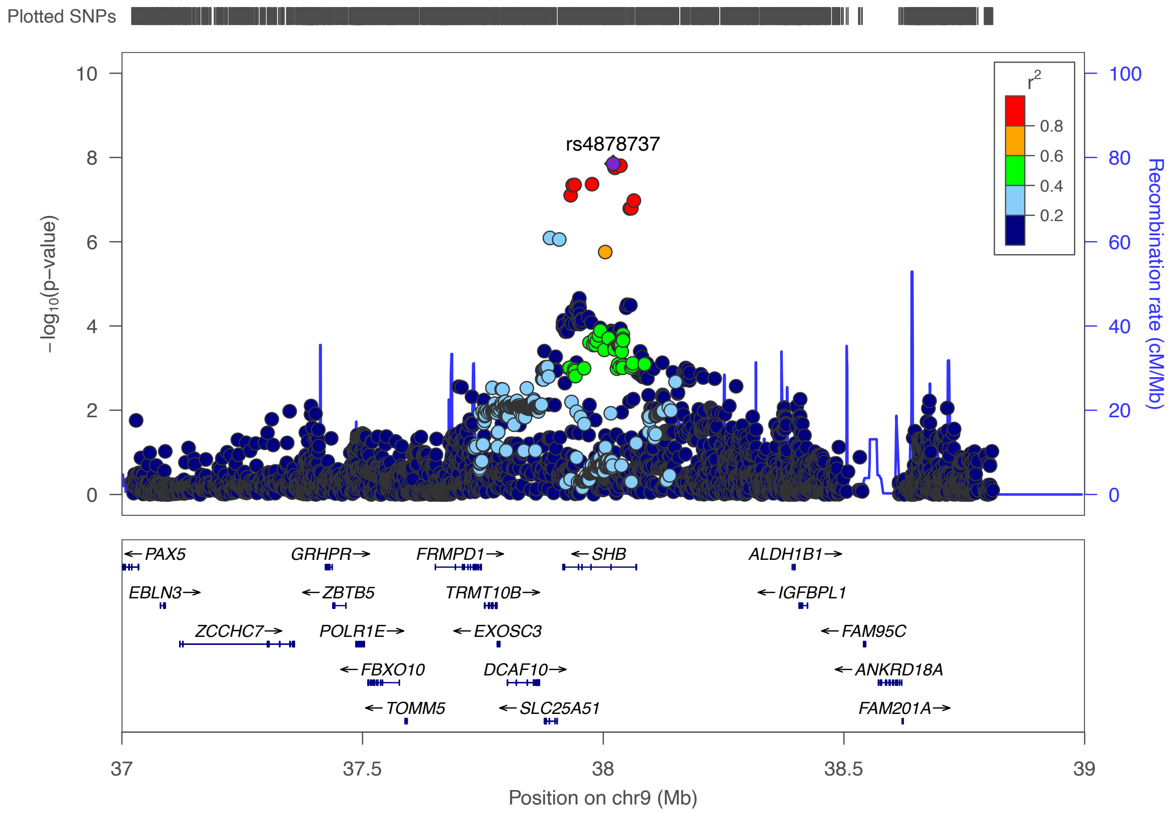

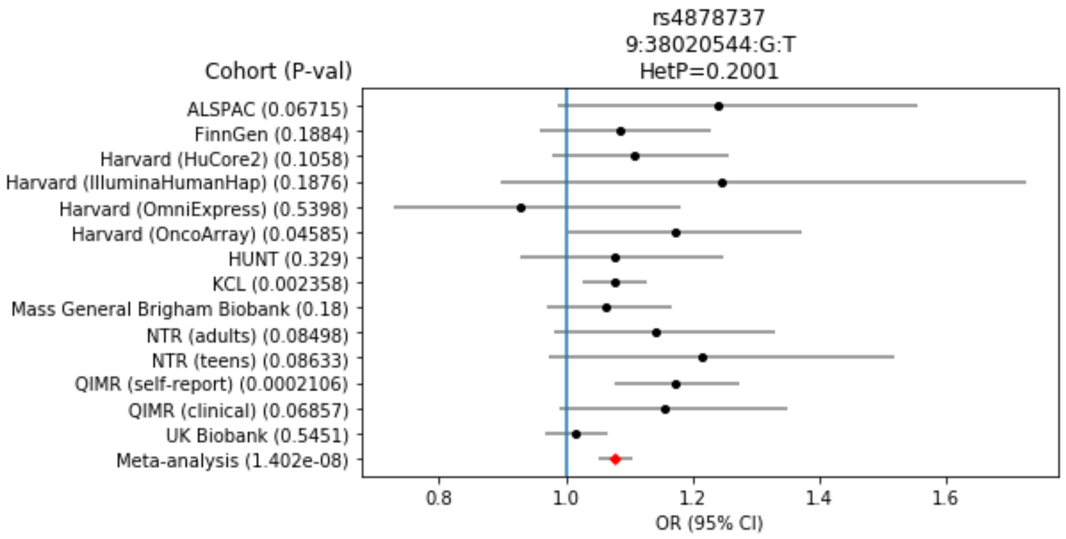

Supplementary Figure 5(xviii): Upper: meta-analysis locuszoom plot for novel 9p13.2 locus. Lower: forest plot for novel 9p13.2 locus, presented as odds ratio +/- 95% confidence intervals. Association *P*-values annotated for meta-analysis (two-sided *Z*-test, not adjusted for multiple testing) and each cohort (calculated as detailed in Supplementary Note).

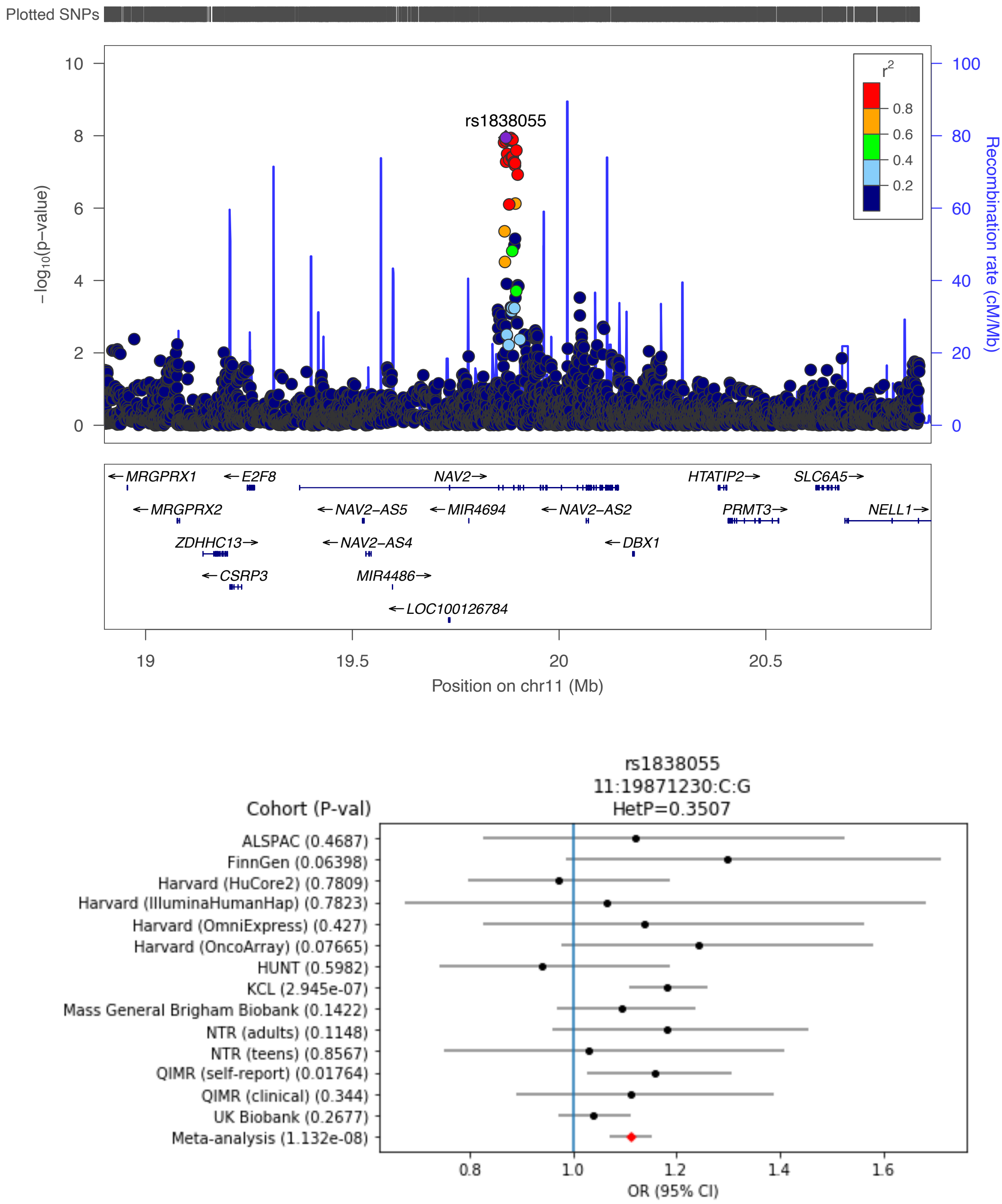

Supplementary Figure 5(xix): Upper: meta-analysis locuszoom plot for novel 11p15.1 locus. Lower: forest plot for novel 11p15.1 locus, presented as odds ratio +/- 95% confidence intervals. Association *P*-values annotated for meta-analysis (two-sided *Z*-test, not adjusted for multiple testing) and each cohort (calculated as detailed in Supplementary Note).

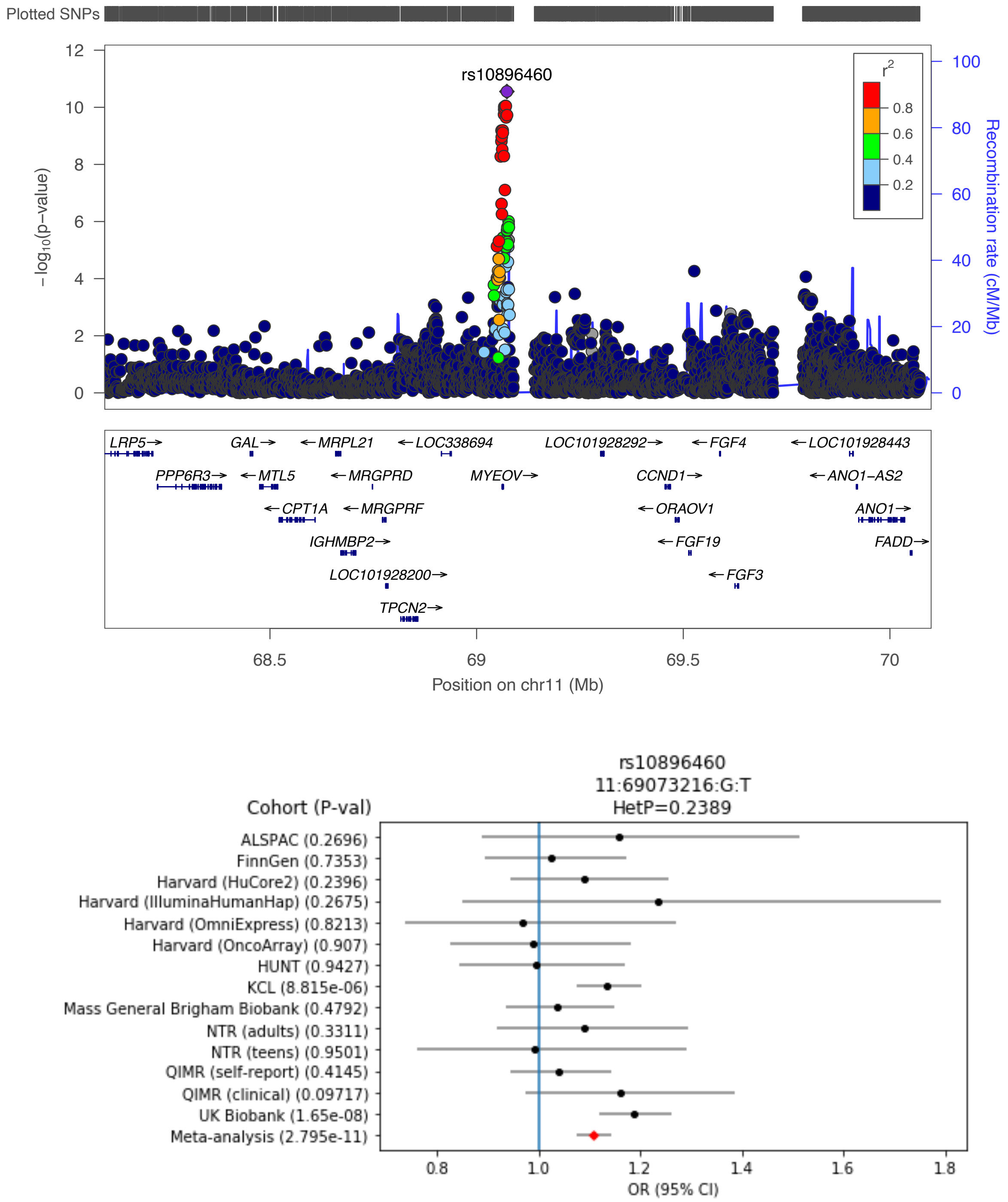

Supplementary Figure 5(xx): Upper: meta-analysis locuszoom plot for novel 11q13.3 locus. Lower: forest plot for novel 11q13.3 locus, presented as odds ratio +/- 95% confidence intervals. Association *P*-values annotated for meta-analysis (two-sided *Z*-test, not adjusted for multiple testing) and each cohort (calculated as detailed in Supplementary Note).

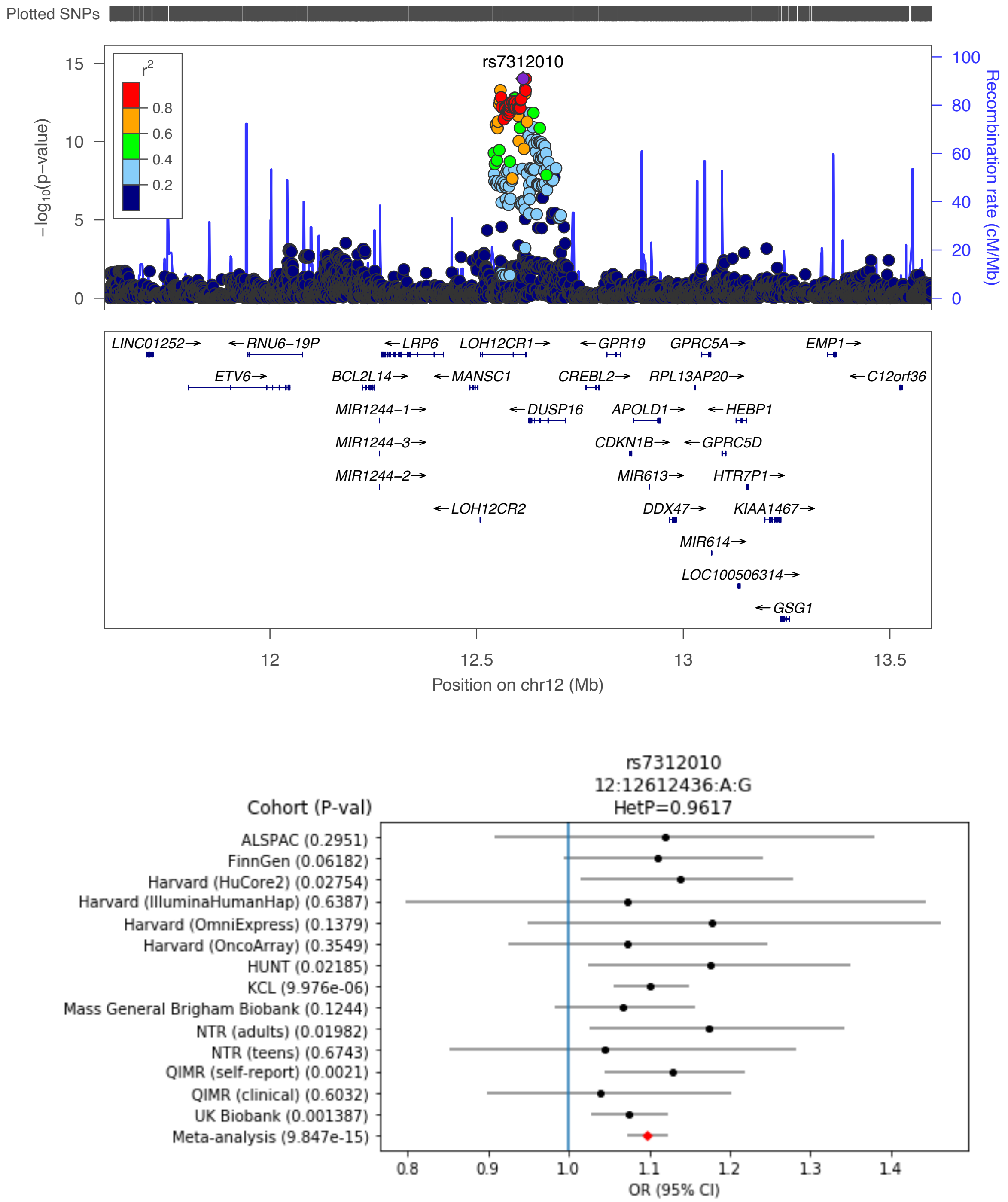

Supplementary Figure 5(xxi): Upper: meta-analysis locuszoom plot for novel 12p13.2 locus. Lower: forest plot for novel 12p13.2 locus, presented as odds ratio +/- 95% confidence intervals. Association *P*-values annotated for meta-analysis (two-sided *Z*-test, not adjusted for multiple testing) and each cohort (calculated as detailed in Supplementary Note).

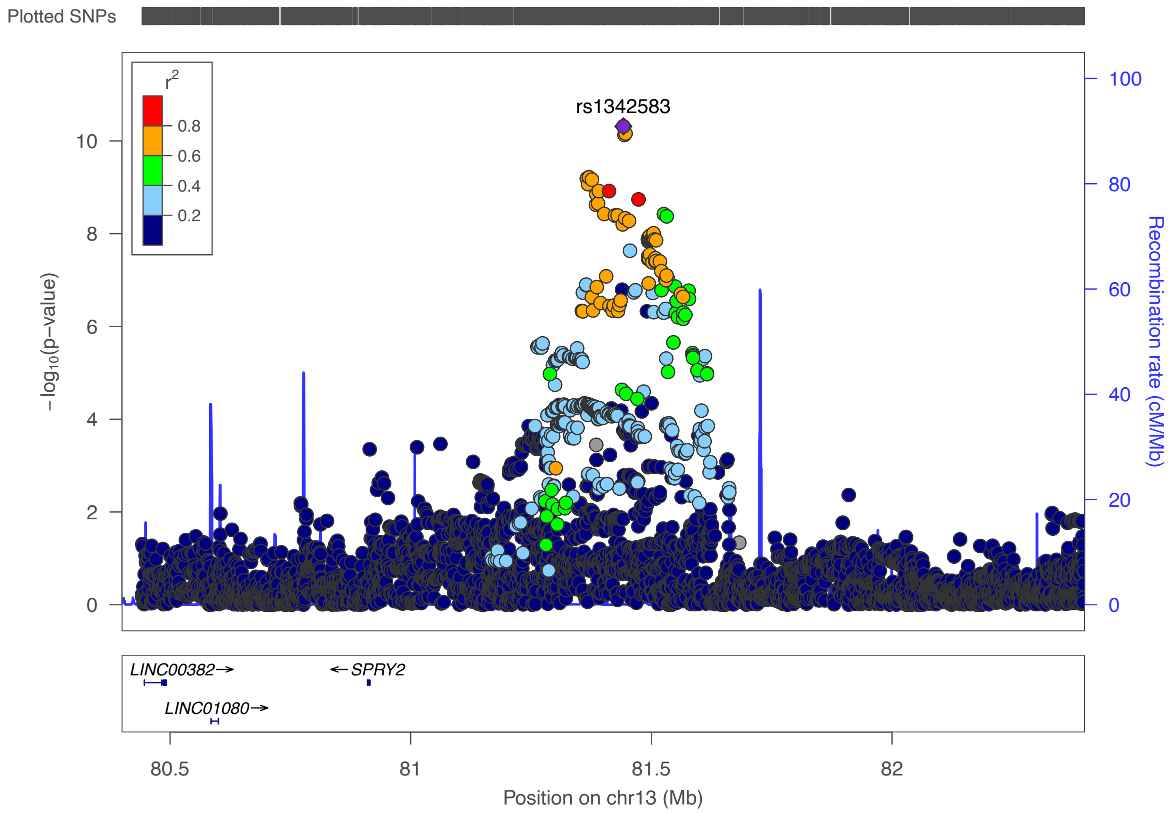

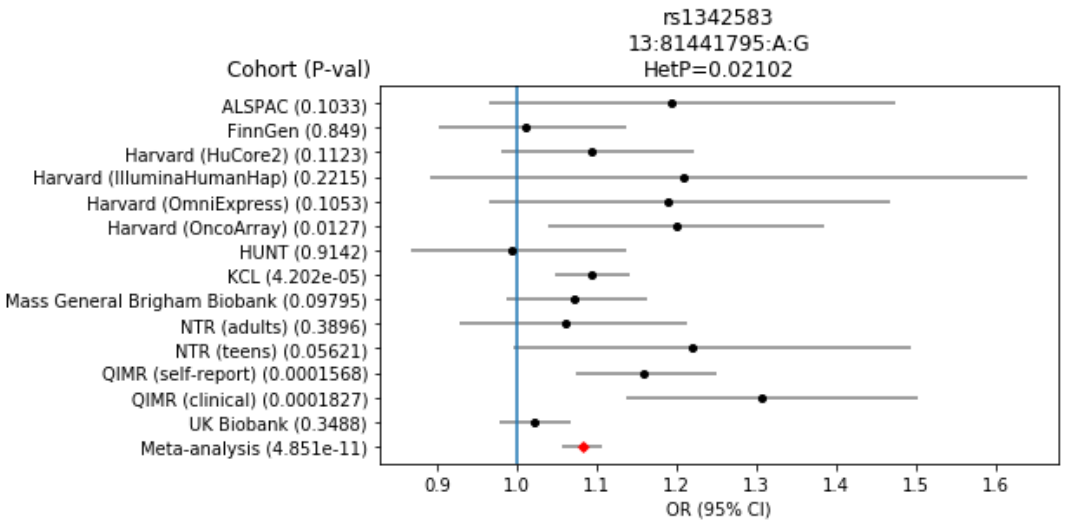

Supplementary Figure 5(xxii): Upper: meta-analysis locuszoom plot for novel 13q31.1 locus. Lower: forest plot for novel 13q31.1 locus, presented as odds ratio +/- 95% confidence intervals. Association *P*-values annotated for meta-analysis (two-sided *Z*-test, not adjusted for multiple testing) and each cohort (calculated as detailed in Supplementary Note).

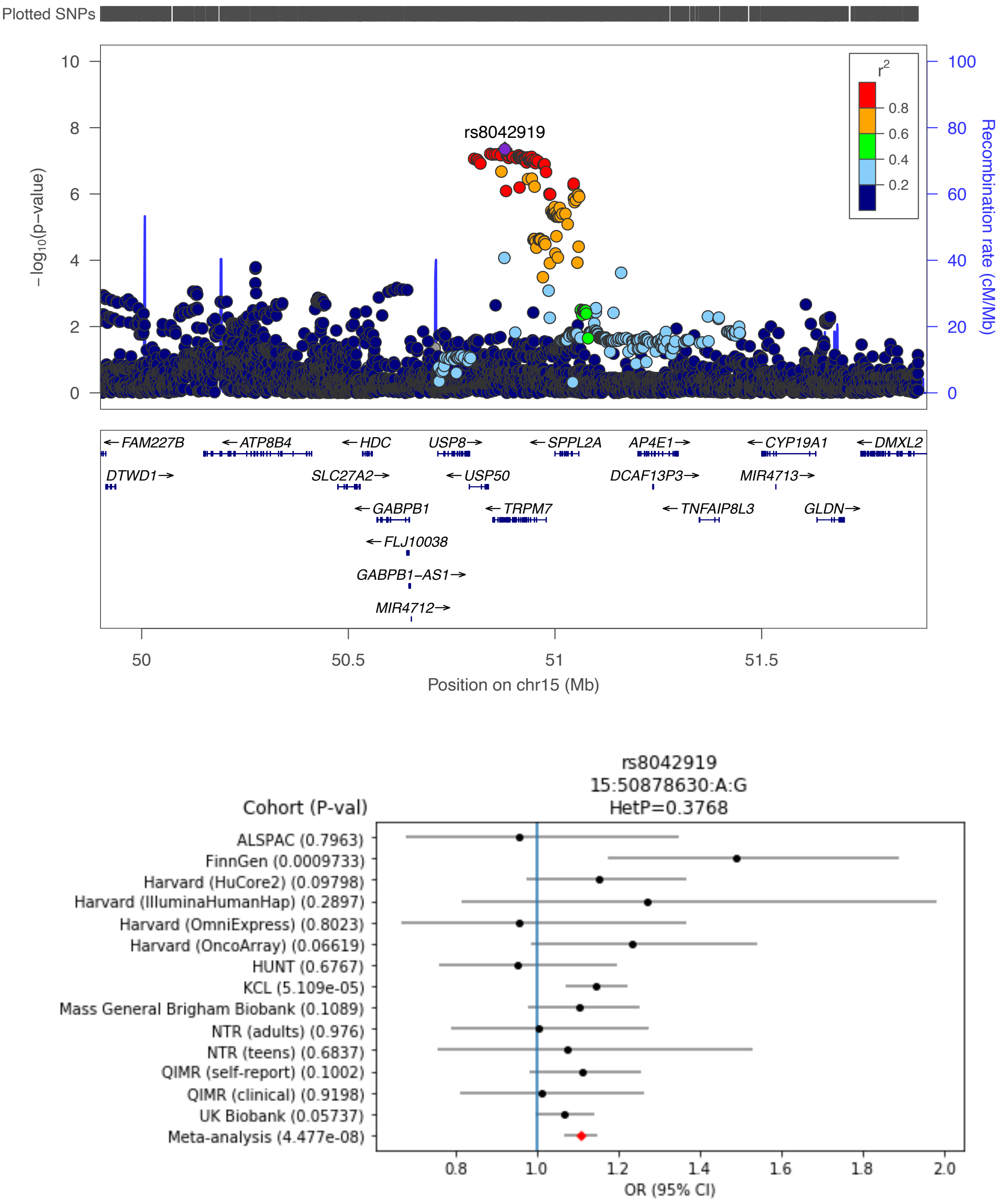

Supplementary Figure 5(xxiii): Upper: meta-analysis locuszoom plot for novel 15q21.2 locus. Lower: forest plot for novel 15q21.2 locus, presented as odds ratio +/- 95% confidence intervals. Association *P*-values annotated for meta-analysis (two-sided *Z*-test, not adjusted for multiple testing) and each cohort (calculated as detailed in Supplementary Note).

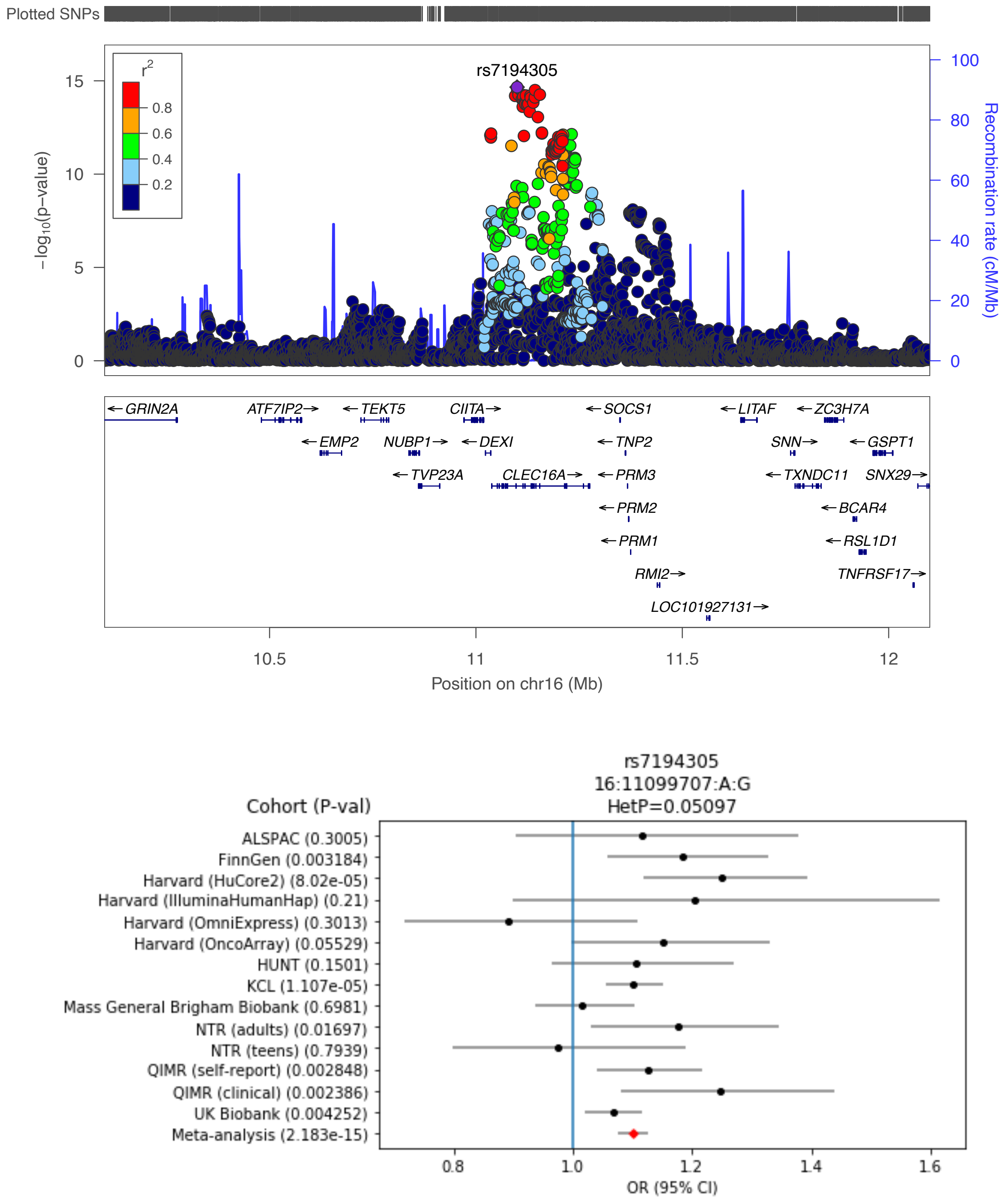

Supplementary Figure 5(xxiv): Upper: meta-analysis locuszoom plot for novel 16p13.13 locus. Lower: forest plot for novel 16p13.13 locus, presented as odds ratio +/- 95% confidence intervals. Association *P*-values annotated for meta-analysis (two-sided *Z*-test, not adjusted for multiple testing) and each cohort (calculated as detailed in Supplementary Note).

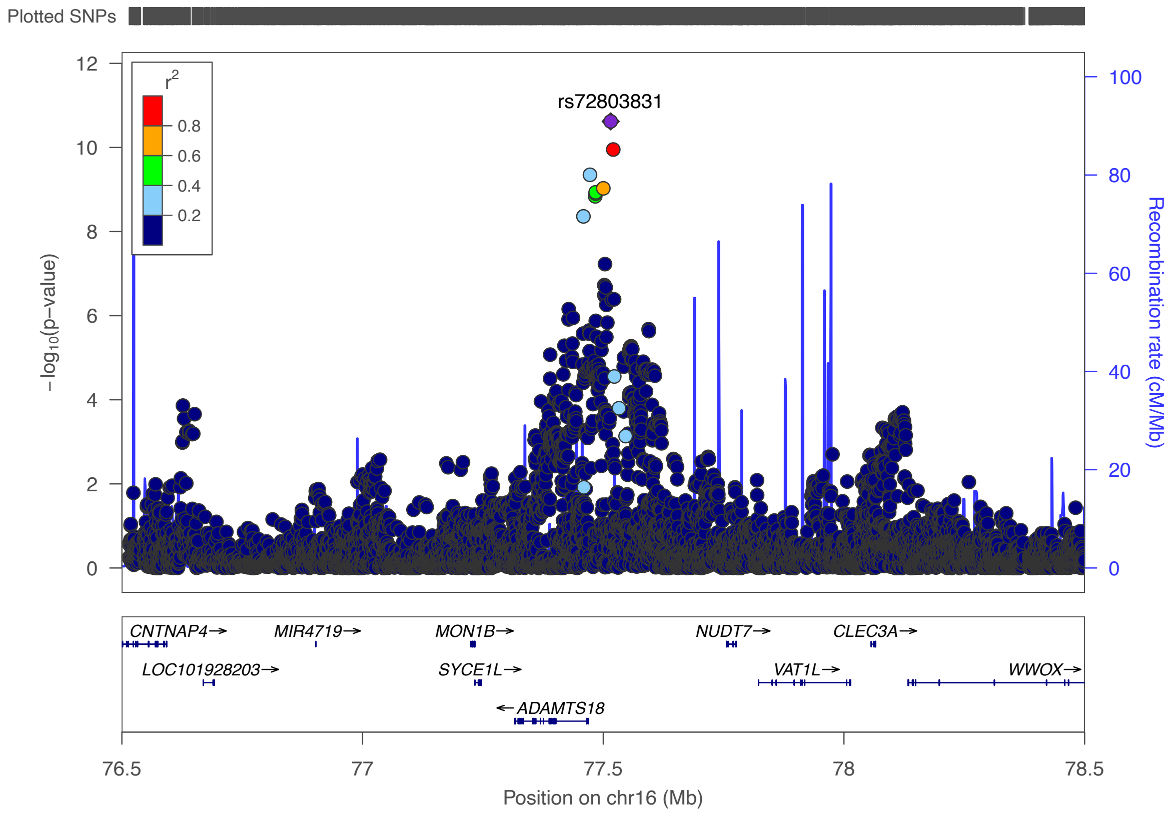

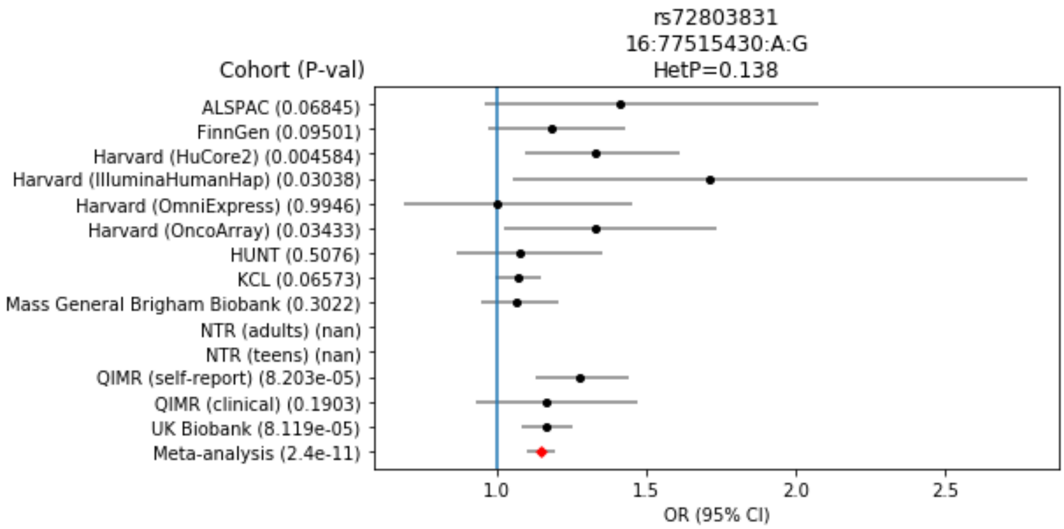

Supplementary Figure 5(xxv): Upper: meta-analysis locuszoom plot for novel 16q23.1 locus. Lower: forest plot for novel 16q23.1 locus, presented as odds ratio +/- 95% confidence intervals. Association *P*-values annotated for meta-analysis (two-sided *Z*-test, not adjusted for multiple testing) and each cohort (calculated as detailed in Supplementary Note).

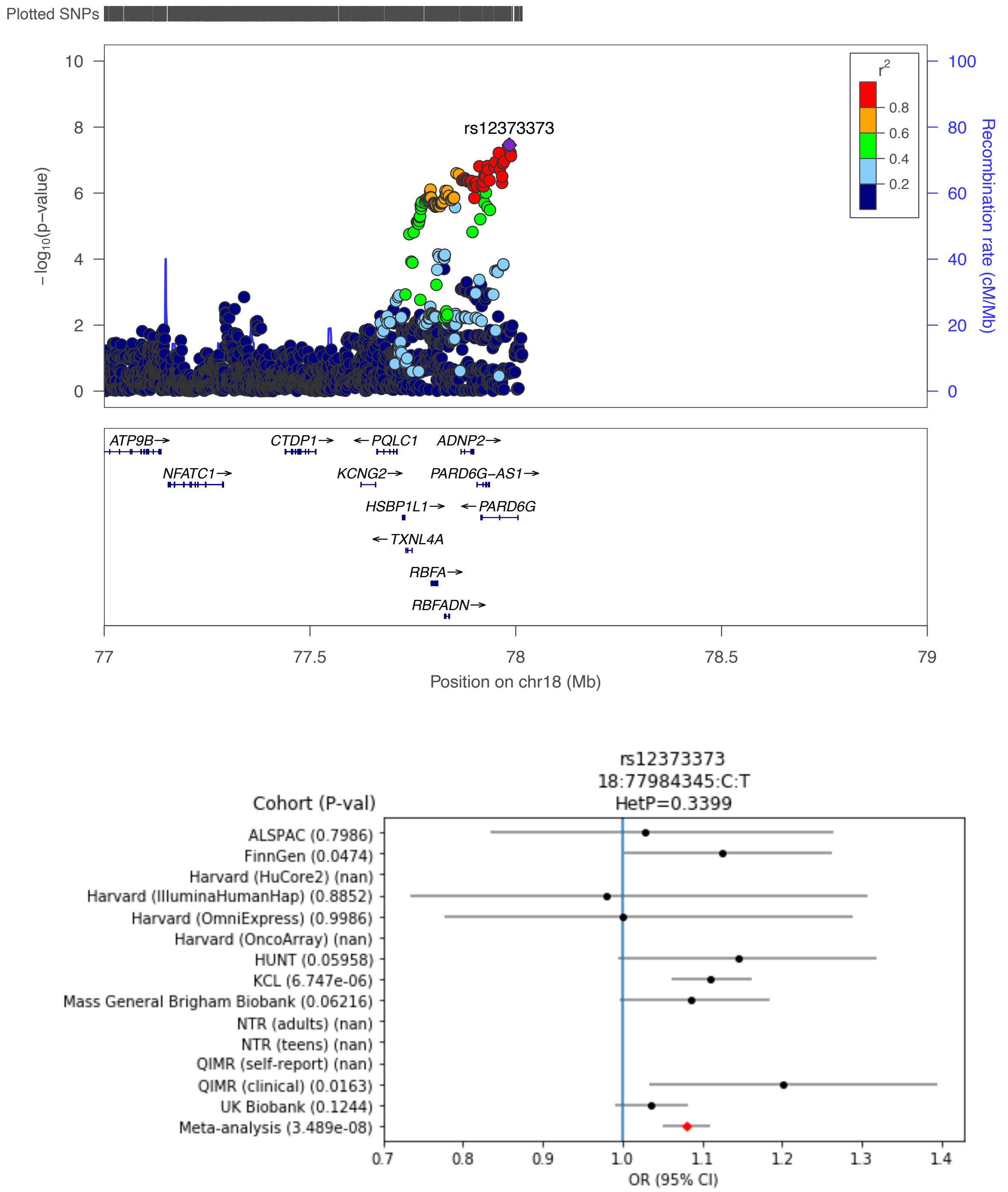

Supplementary Figure 5(xxvi): Upper: meta-analysis locuszoom plot for novel 18q23 locus. Lower: forest plot for novel 18q23 locus, presented as odds ratio +/- 95% confidence intervals. Association *P*-values annotated for meta-analysis (two-sided *Z*-test, not adjusted for multiple testing) and each cohort (calculated as detailed in Supplementary Note).

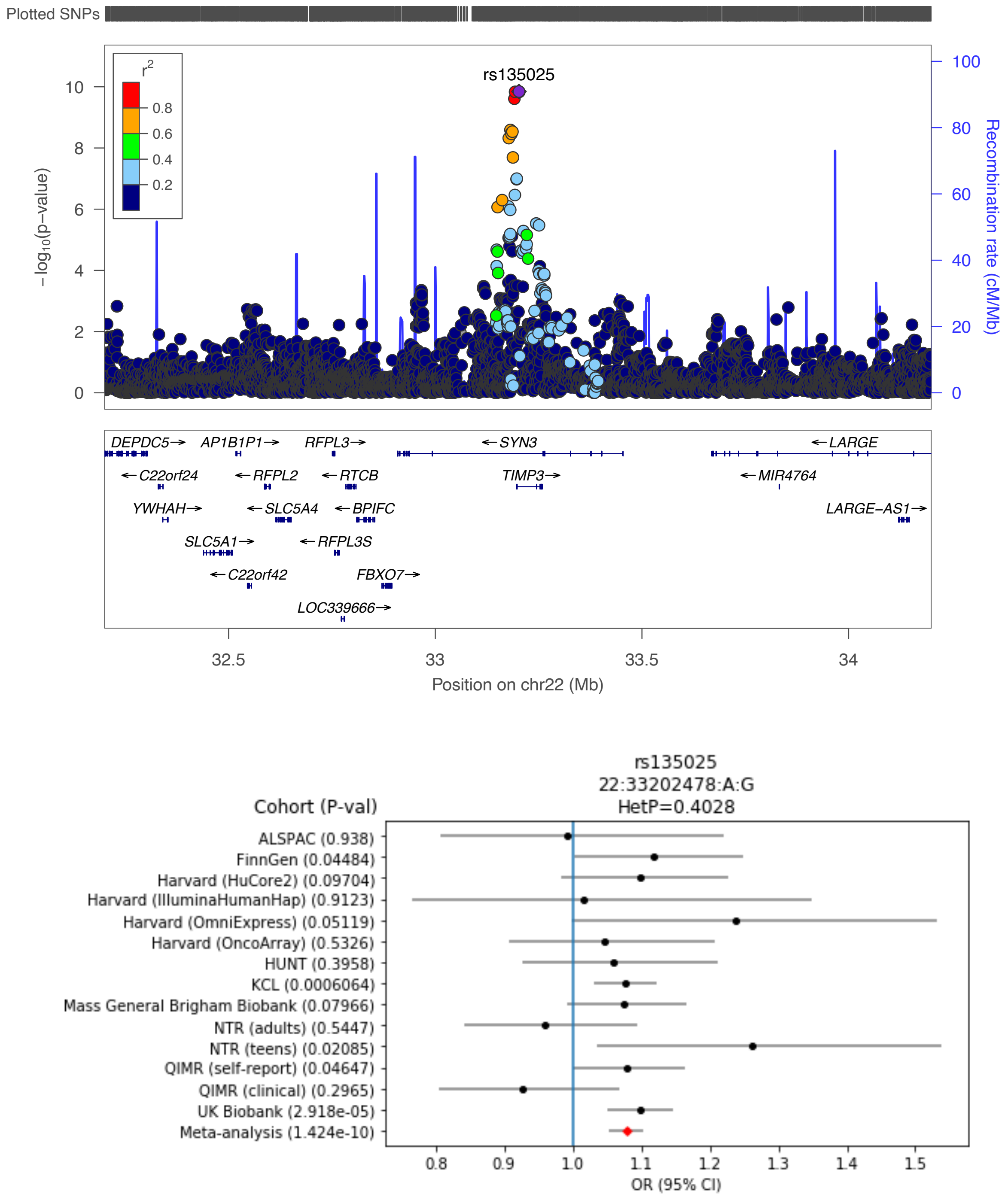

Supplementary Figure 5(xxvii): Upper: meta-analysis locuszoom plot for novel 22q12.3 locus. Lower: forest plot for novel 22q12.3 locus, presented as odds ratio +/- 95% confidence intervals. Association *P*-values annotated for meta-analysis (two-sided *Z*-test, not adjusted for multiple testing) and each cohort (calculated as detailed in Supplementary Note).

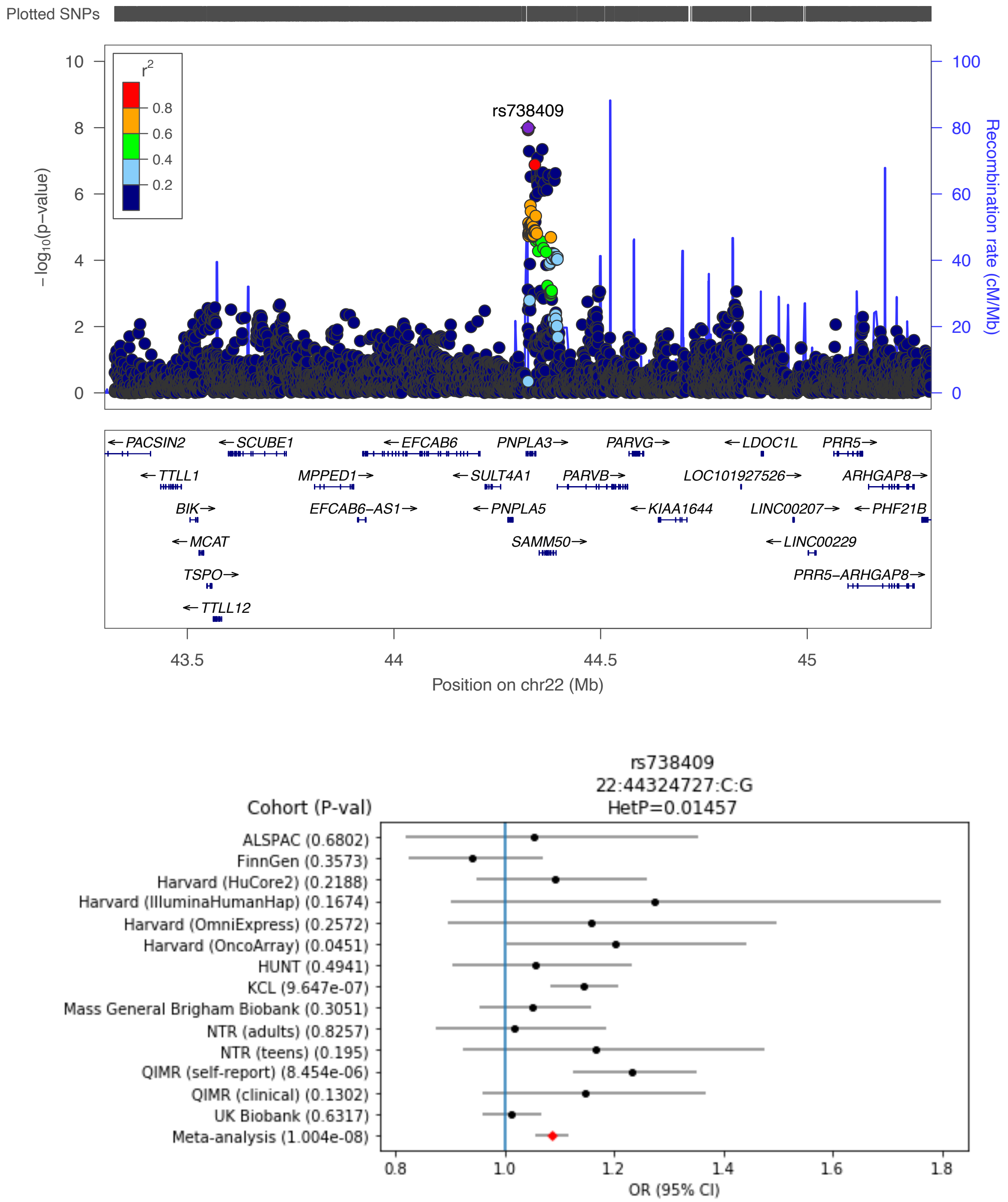

Supplementary Figure 5(xxviii): Upper: meta-analysis locuszoom plot for novel 22q13.31 locus. Lower: forest plot for novel 22q13.31 locus, presented as odds ratio +/- 95% confidence intervals. Association *P*-values annotated for meta-analysis (two-sided *Z*-test, not adjusted for multiple testing) and each cohort (calculated as detailed in Supplementary Note).

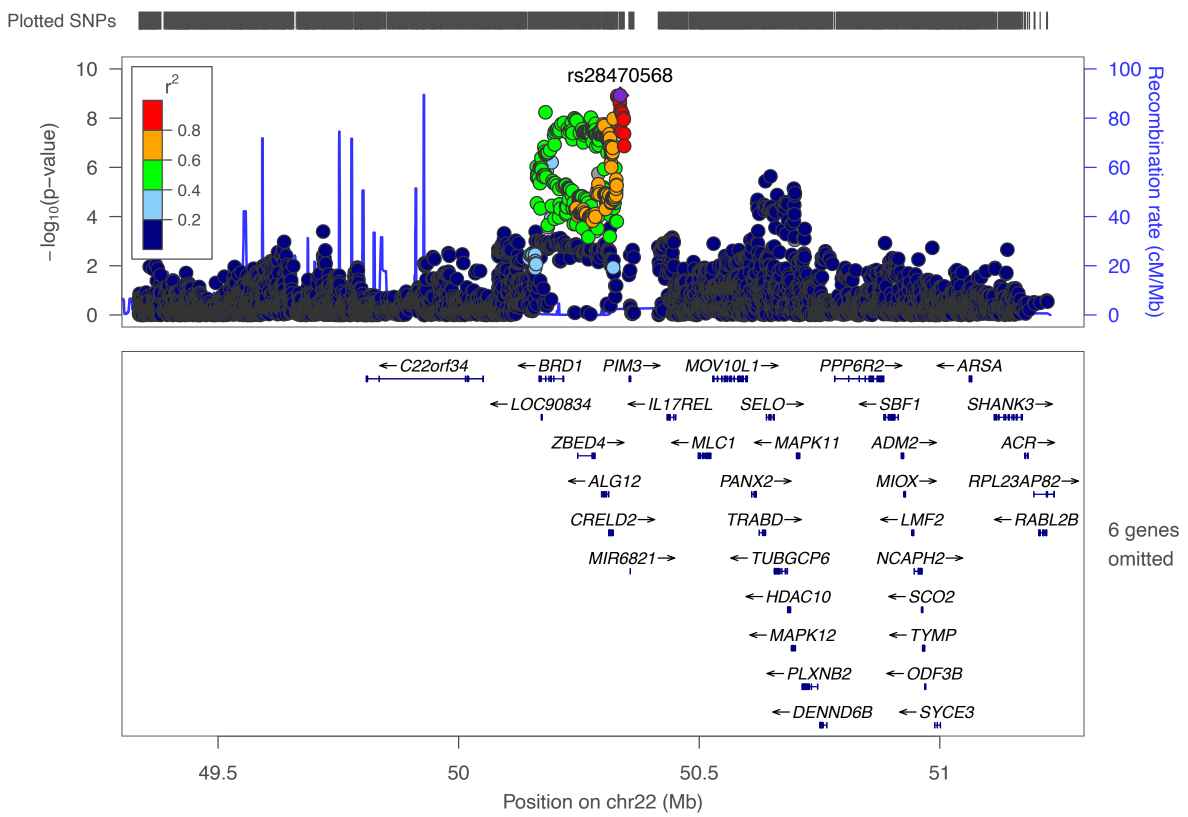

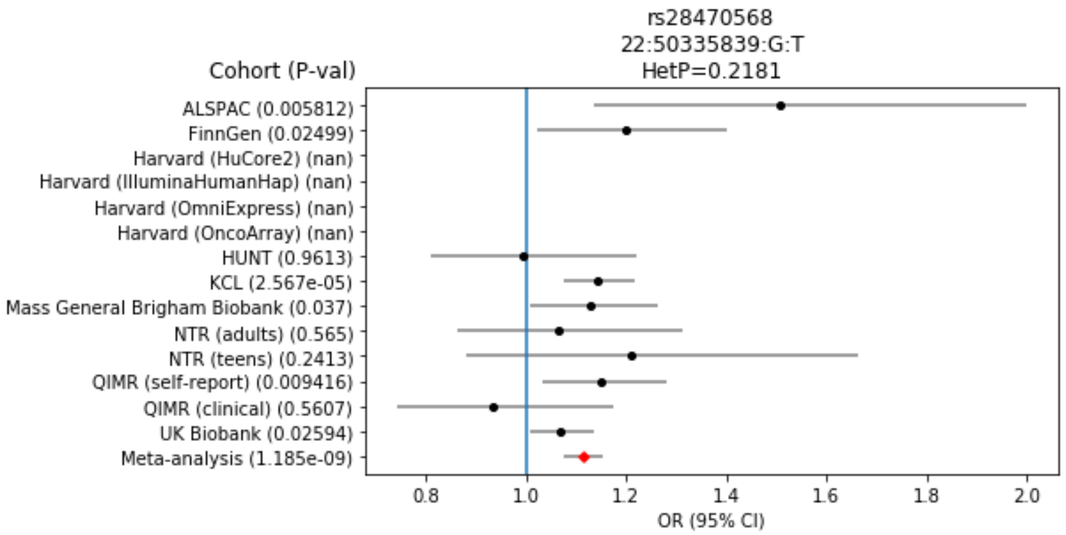

Supplementary Figure 5(xxix): Upper: meta-analysis locuszoom plot for novel 22q13.33 locus. Lower: forest plot for novel 22q13.33 locus, presented as odds ratio +/- 95% confidence intervals. Association *P*-values annotated for meta-analysis (two-sided *Z*-test, not adjusted for multiple testing) and each cohort (calculated as detailed in Supplementary Note).

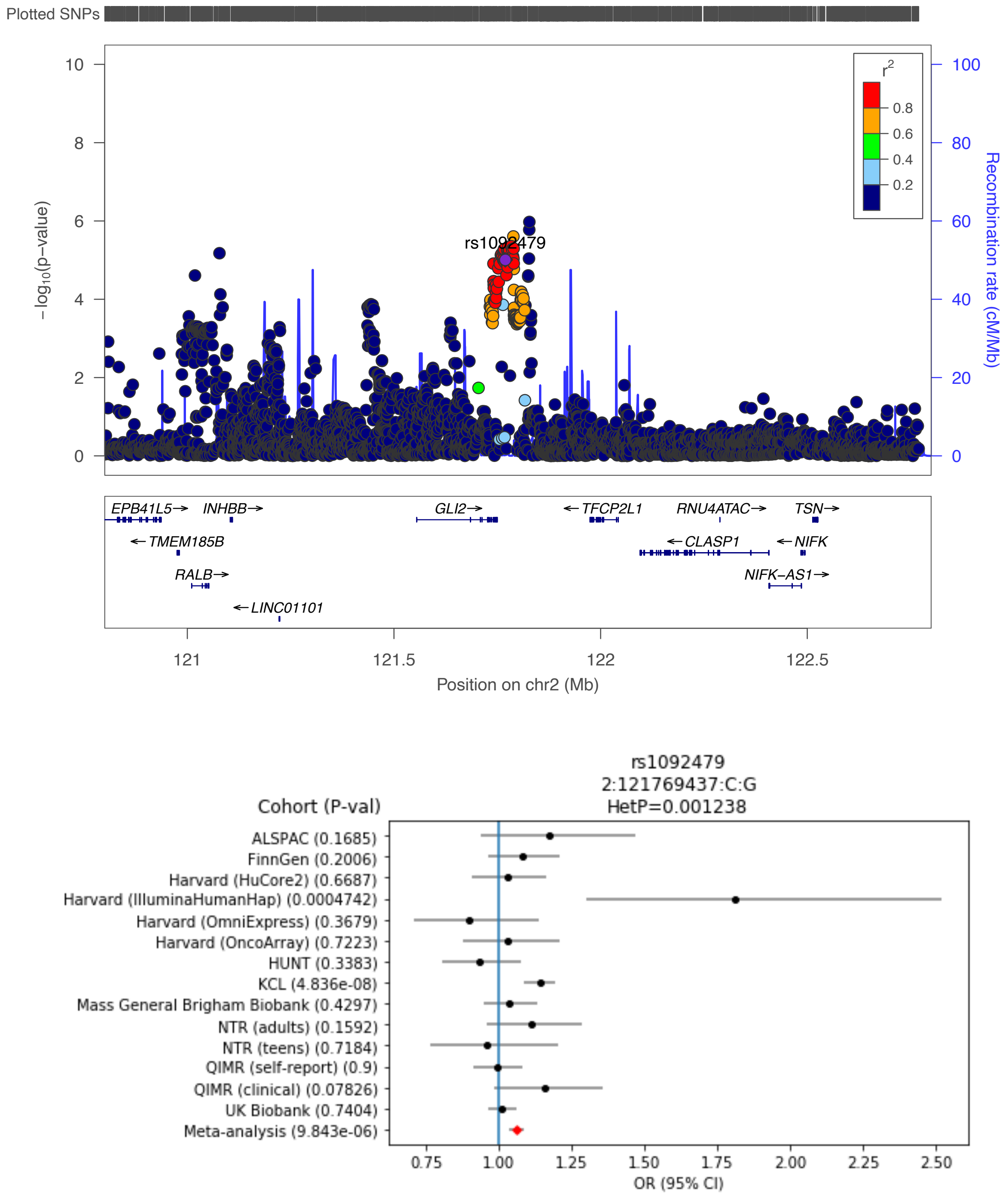

Supplementary Figure 6(i): Upper: meta-analysis locuszoom plot for Petridis et al. 2q14.2 locus. Lower: forest plot for Petridis et al. 2q14.2 locus, presented as odds ratio +/- 95% confidence intervals. Association *P*-values annotated for meta-analysis (two-sided *Z*-test, not adjusted for multiple testing) and each cohort (calculated as detailed in Supplementary Note).

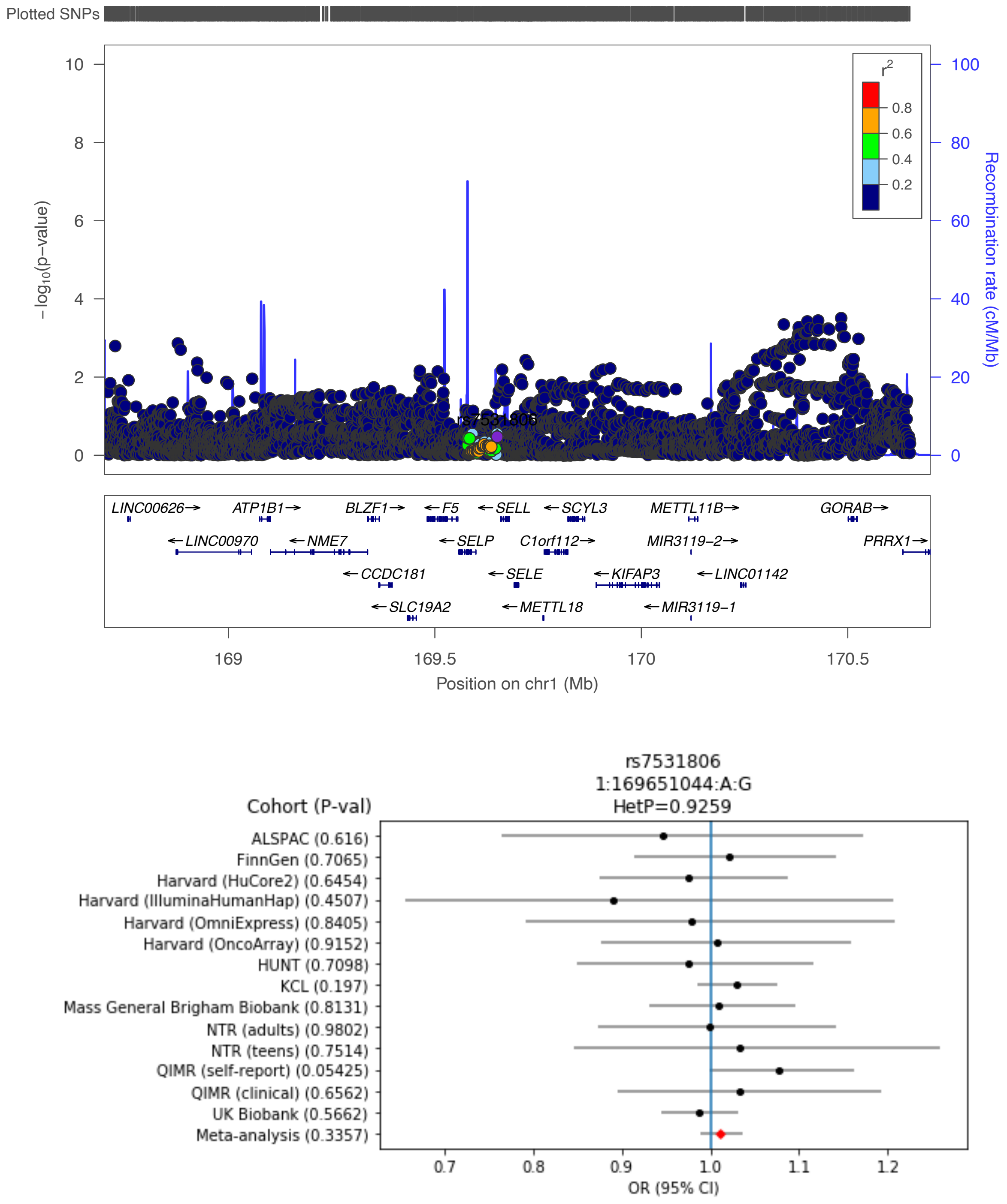

Supplementary Figure 6(ii): Upper: meta-analysis locuszoom plot for He et al. 1q24.2 locus. Lower: forest plot for He et al. 1q24.2 locus, presented as odds ratio +/- 95% confidence intervals. Association *P*-values annotated for meta-analysis (two-sided *Z*-test, not adjusted for multiple testing) and each cohort (calculated as detailed in Supplementary Note).

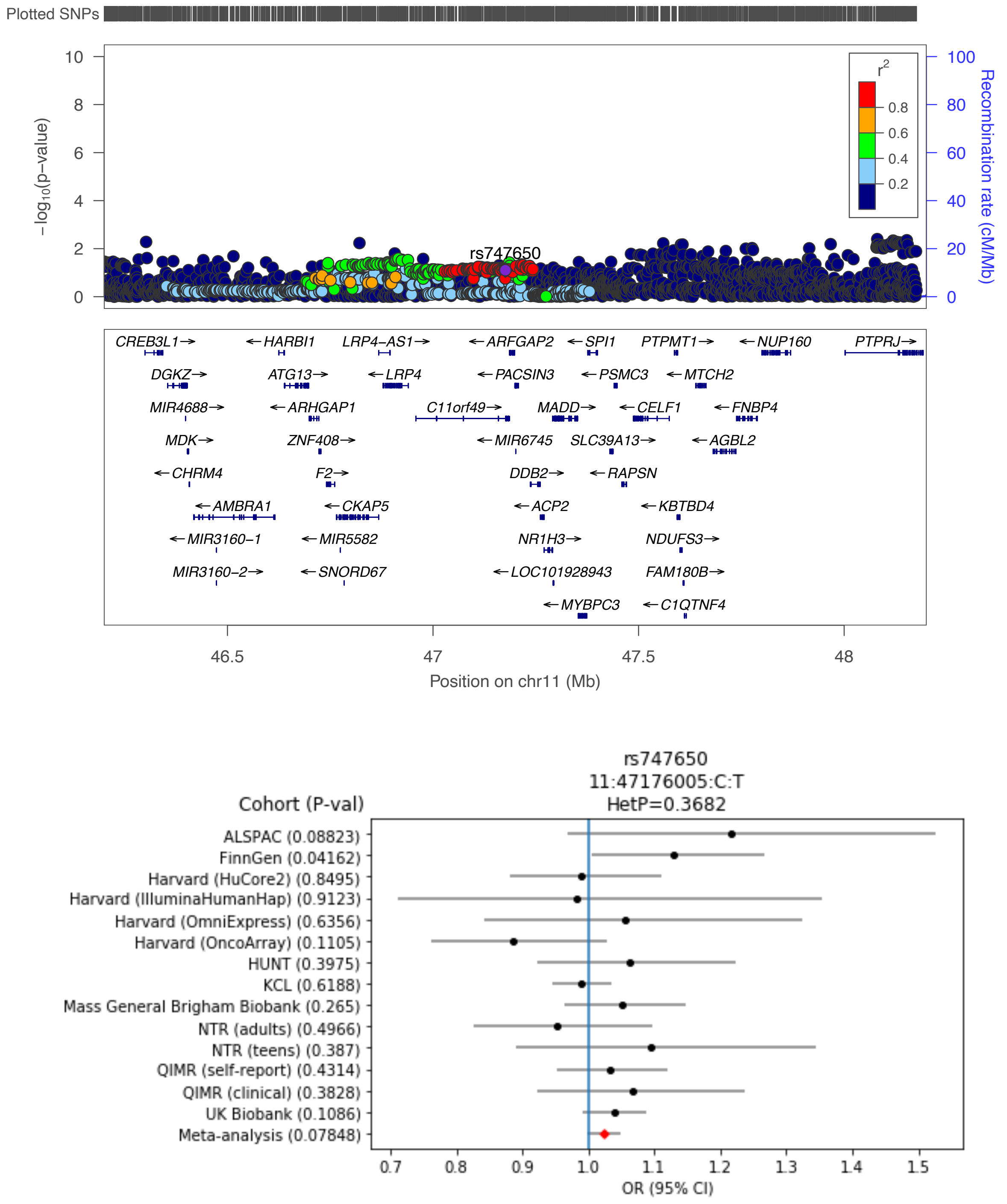

Supplementary Figure 6(iii): Upper: meta-analysis locuszoom plot for He et al. 11p11.2 locus. Lower: forest plot for He et al. 11p11.2 locus, presented as odds ratio +/- 95% confidence intervals. Association *P*-values annotated for meta-analysis (two-sided *Z*-test, not adjusted for multiple testing) and each cohort (calculated as detailed in Supplementary Note).

References

1. Fraser, A. *et al.* Cohort Profile: the Avon Longitudinal Study of Parents and Children: ALSPAC mothers cohort. *Int. J. Epidemiol.* **42**, 97–110 (2013).
2. Boyd, A. *et al.* Cohort Profile: the ‘children of the 90s’--the index offspring of the Avon Longitudinal Study of Parents and Children. *Int. J. Epidemiol.* **42**, 111–127 (2013).
3. McCarthy, S. *et al.* A reference panel of 64,976 haplotypes for genotype imputation. *Nat. Genet.* **48**, 1279–1283 (2016).
4. Das, S. *et al.* Next-generation genotype imputation service and methods. *Nat. Genet.* **48**, 1284–1287 (2016).
5. O’brien, S., Lewis, J. & Cunliffe, W. The Leeds revised acne grading system. *J. Dermatolog. Treat.* **9**, 215–220 (1998).
6. Bao, Y. *et al.* Origin, Methods, and Evolution of the Three Nurses’ Health Studies. *Am. J. Public Health* **106**, 1573–1581 (2016).
7. Lee, D. H., de Rezende, L. F. M., Hu, F. B., Jeon, J. Y. & Giovannucci, E. L. Resting heart rate and risk of type 2 diabetes: A prospective cohort study and meta-analysis. *Diabetes. Metab. Res. Rev.* **35**, e3095 (2019).
8. Zhan, X., Hu, Y., Li, B., Abecasis, G. R. & Liu, D. J. RVTESTS: an efficient and comprehensive tool for rare variant association analysis using sequence data. *Bioinformatics* **32**, 1423–1426 (2016).
9. Krokstad, S. *et al.* Cohort Profile: the HUNT Study, Norway. *Int. J. Epidemiol.* **42**, 968–977 (2013).
10. Jun, G. *et al.* Detecting and estimating contamination of human DNA samples in sequencing and array-based genotype data. *Am. J. Hum. Genet.* **91**, 839–848 (2012).

11. Zhou, W. *et al.* Efficiently controlling for case-control imbalance and sample relatedness in large-scale genetic association studies. *Nat. Genet.* **50**, 1335–1341 (2018).
12. Navarini, A. A. *et al.* Genome-wide association study identifies three novel susceptibility loci for severe Acne vulgaris. *Nat. Commun.* **5**, 4020 (2014).
13. Karlson, E. W., Boutin, N. T., Hoffnagle, A. G. & Allen, N. L. Building the partners healthcare biobank at partners personalized medicine: informed consent, return of research results, recruitment lessons and operational considerations. *Journal of personalized medicine* **6**, 2 (2016).
14. Ligthart, L. *et al.* The Netherlands Twin Register: Longitudinal Research Based on Twin and Twin-Family Designs. *Twin Res. Hum. Genet.* **22**, 623–636 (2019).
15. Scheet, P. *et al.* Twins, tissue, and time: an assessment of SNPs and CNVs. *Twin Res. Hum. Genet.* **15**, 737–745 (2012).
16. Willemsen, G. *et al.* The Netherlands Twin Register biobank: a resource for genetic epidemiological studies. *Twin Res. Hum. Genet.* **13**, 231–245 (2010).
17. Vink, J. M., Sadrzadeh, S., Lambalk, C. B. & Boomsma, D. I. Heritability of polycystic ovary syndrome in a Dutch twin-family study. *J. Clin. Endocrinol. Metab.* **91**, 2100–2104 (2006).
18. Boomsma, D. I. *et al.* The Genome of the Netherlands: design, and project goals. *Eur. J. Hum. Genet.* **22**, 221–227 (2014).
19. Purcell, S. *et al.* PLINK: a tool set for whole-genome association and population-based linkage analyses. *Am. J. Hum. Genet.* **81**, 559–575 (2007).
20. Deelen, P. *et al.* Improved imputation quality of low-frequency and rare variants in European samples using the 'Genome of The Netherlands'. *Eur. J. Hum. Genet.* **22**, 1321–1326 (2014).

21. Abdellaoui, A. *et al.* Population structure, migration, and diversifying selection in the Netherlands. *Eur. J. Hum. Genet.* **21**, 1277–1285 (2013).
22. Delaneau, O., Marchini, J. & Zagury, J.-F. A linear complexity phasing method for thousands of genomes. *Nat. Methods* **9**, 179–181 (2011).
23. Byrne, E. M. *et al.* Cohort profile: the Australian genetics of depression study. *BMJ Open* **10**, e032580 (2020).
24. Wright, M. J. & Martin, N. G. Brisbane Adolescent Twin Study: Outline of study methods and research projects. *Aust. J. Psychol.* **56**, 65–78 (2004).
25. Medland, S. E. *et al.* Common variants in the trichohyalin gene are associated with straight hair in Europeans. *Am. J. Hum. Genet.* **85**, 750–755 (2009).
26. Lupton, M. K. *et al.* A prospective cohort study of prodromal Alzheimer's disease: Prospective Imaging Study of Ageing: Genes, Brain and Behaviour (PISA). *Neuroimage Clin* **29**, 102527 (2021).
27. Sudlow, C. *et al.* UK biobank: an open access resource for identifying the causes of a wide range of complex diseases of middle and old age. *PLoS Med.* **12**, e1001779 (2015).
28. Bycroft, C. *et al.* The UK Biobank resource with deep phenotyping and genomic data. *Nature* **562**, 203–209 (2018).
