# Supplementary material for: Genome-wide association meta-analysis identifies 29 new acne susceptibility loci
Source: Nat Commun. 2022 Feb 7;13:702. doi: 10.1038/s41467-022-28252-5 (PMC8821634; doi:10.1038/s41467-022-28252-5)
Supplement: Supplementary file 3 — Description of Additional Supplementary Files [file 41467_2022_28252_MOESM3_ESM.pdf]

Description of Additional Supplementary Files

File Name: Supplementary Data 1
Description: Summary of acne cohorts in the meta-analysis.

File Name: Supplementary Data 2
Description: Summary statistics of three loci previously associated with acne that are not replicated in the meta-analysis.

File Name: Supplementary Data 3
Description: Co-localisation of GTEX sun exposed and non-exposed skin eQTLs at loci identified to be associated with acne through the meta-analysis

File Name: Supplementary Data 4
Description: Protein altering variants in the 95% credible sets of genome-wide significant loci.

File Name: Supplementary Data 5
Description: Genes prioritised to be implicated in acne identified through DEPICT analysis.

File Name: Supplementary Data 6
Description: Gene-sets prioritised to be implicated in acne identified through DEPICT analysis.

File Name: Supplementary Data 7
Description: Significant genetic correlation (rG) and corresponding genetic causal proportion (GCP) estimates for acne and 45 traits identified through ld-score regression and latent causal variable analysis.
